# Optimal Choice of the Regularization Parameter for Direct Identification of Polymers Relaxation Time and Frequency Spectra

**DOI:** 10.3390/polym17010031

**Published:** 2024-12-26

**Authors:** Anna Stankiewicz, Monika Bojanowska

**Affiliations:** 1 Department of Technology Fundamentals, Faculty of Production Engineering, University of Life Sciences in Lublin, 20-612 Lublin, Poland; anna.m.stankiewicz@gmail.com; 2 Department of Chemistry, Faculty of Food Science and Biotechnology, University of Life Sciences in Lublin, 20-950 Lublin, Poland

**Keywords:** viscoelasticity, relaxation spectra, linear relaxation modulus, direct spectrum identification, integral-empirical square index, Tikhonov regularization, regularization parameter selection

## Abstract

Recovering the relaxation spectrum, a fundamental rheological characteristic of polymers, from experiment data requires special identification methods since it is a difficult ill-posed inverse problem. Recently, a new approach relating the identification index directly with a completely unknown real relaxation spectrum has been proposed. The integral square error of the relaxation spectrum model was applied. This paper concerns regularization aspects of the linear-quadratic optimization task that arise from applying Tikhonov regularization to relaxation spectra direct identification problem. An influence of the regularization parameter on the norms of the optimal relaxation spectra models and on the fit of the related relaxation modulus model to the experimental data was investigated. The trade-off between the integral square norms of the spectra models and the mean square error of the relaxation modulus model, parameterized by varying regularization parameter, motivated the definition of two new multiplicative indices for choosing the appropriate regularization parameter. Two new problems of the regularization parameter optimal selection were formulated and solved. The first and second order optimality conditions were derived and expressed in the matrix-vector form and, alternatively, in finite series terms. A complete identification algorithm is presented. The usefulness of the new regularization parameter selection rules is demonstrated by three examples concerning the Kohlrausch–Williams–Watts spectrum with short relaxation times and uni- and double-mode Gauss-like spectra with middle and short relaxation times.

## 1. Introduction

The relaxation time and frequency spectra are essential for constitutive models and for providing insight into the mechanical properties of polymers since from the spectra other viscoelastic characteristics used to describe mechanical properties can be uniquely derived [1,2,3,4]. The spectra are applied for description, analysis, and to accomplish desirable mechanical properties of the designed polymers [2,3]. However, the spectra are not directly available by measurement. To determine them, based on relaxation stress or oscillatory shear relaxation data, an ill-posed inverse problem must be solved, the solutions to which, if there are any, are very sensitive to even slight changes in the experiment data, leading to disastrous changes in the determined relaxation spectrum. Therefore, typical methods for identifying dynamic systems cannot be applied here. Special stable and noise robust algorithms are required to determine the relaxation spectrum models.

The first papers concerning recovery of the relaxation time and frequency spectra from the measurements of the relaxation modulus obtained in the stress relaxation test are dated to the turn of the 1940s and 1950s [5,6,7]. Since then, many theoretical [8,9,10,11,12,13,14] and experimental [15,16,17,18,19] studies have been conducted regarding relaxation spectra identification. Miscellaneous analytical and numerical tools have been applied. As a result, several classes of relaxation spectra models and appropriate identification algorithms have been investigated for both stress relaxation and dynamic oscillatory experiments data. Other references corresponding to the relaxation spectrum determination, together with various classifications and reviews of these models, methods, and algorithms can be found, for example, in [20,21,22] and, most recently, in [23,24,25,26].

Most of the known methods use the least squares identification concept, applying the mean sum of square errors between the relaxation modulus models and the modulus measurements obtained in the stress relaxation test [25,26,27,28,29] and the mean sum of square errors between the storage and/or loss moduli models and the dynamic moduli measurements resulted from the oscillatory shear experiment [8,10,11,12,14,15,16,17,18,19,20,30,31]. Both the pure least-squares identification, e.g., [15,27,32], and the regularized least-squares, for example [10,11,20,25,26], were applied. In these methods, the model quality criteria—mean sums of the model errors—were related to the process variables available by measurements, i.e., the relaxation or dynamic moduli, depending on the type of rheological experiment. Therefore, the quality of the relaxation spectra models was estimated only in an indirect manner, by the mean square errors of the relaxation modulus or dynamic moduli models, the minimization of which was an essential step of the identification algorithms. A different approach was used in [23], where the best smoothed spectrum model, which reproduces the relaxation modulus measurements with a small error of the relaxation modulus model, was determined by minimizing the integral square norm of the spectrum model. However, this means that the identification criterion refers, as in previously known paper, to the spectrum model and not to the unknown real spectrum. The model error is assessed with respect to the relaxation modulus model, i.e., to the model of variable being measurement-available.

Recently, in [24], a new approach was proposed based on direct approximation of the real completely unknown relaxation time spectrum by a series of appropriately selected basis functions. It was assumed that only discrete-time noise-corrupted measurements of a relaxation modulus obtained in the stress relaxation experiment are available for identification. The modified relaxation frequency spectrum, being defined as a quotient of the real relaxation spectrum and relaxation frequency [33], was expanded into a series of exponential functions (i.e., the kernel of the Laplace transformation), forming a basis of the space of square-integrable functions [34], which is equivalent to the expansion of the relaxation time spectrum into a series of the basis functions, being the product of the relaxation frequency and exponential function of it. The spectra models, described by finite series of this basis functions, were adopted. As a measure of the model quality, an integral square error between the real unknown spectrum and the spectrum model was assumed. This index was proved to be expressed in terms of the measurable relaxation modulus at uniquely defined sampling instants. It was also proved that the integral-square indices for the relaxation time spectrum and the modified frequency spectrum are equivalent. By replacing the real relaxation modulus by their noise measurements, an integral-empirical identification index was obtained, the minimization of which is the essence of direct relaxation spectrum identification. The direct identification problem turned out to be a linear-quadratic optimization task for which Tikhonov regularization was used to guarantee the model smoothness and noise robustness. The use of appropriately selected basis functions of the relaxation spectrum model allowed for linking the model quality index, related directly to the unknown spectrum, with the relaxation modulus measurements. In consequence, the identification index being minimized, although expressed in terms of the relaxation modulus measurements, refers directly to unknown relaxation spectrum, not to the measured relaxation modulus. This new approach was proposed and used in [24] for the first time.

It is generally accepted that the choice of the respective regularization parameter is important to identify the best model. Both the spectra model’s smoothness and the errors of the spectra and modulus models depend on the value of the regularization parameter. Therefore, the aim of the present paper is to develop a regularization parameter selection rule that can be applied for relaxation spectra direct identification.

In [24], a simple rule based on the value of the spectral conditional number of regularized basic matrix of the linear-quadratic direct spectrum identification task was applied. However, there are well-studied techniques for computing a good regularization parameter, such as generalized cross-validation (GCV) [35,36] and the L-curve technique [35,37]. These methods have been developed for classical least-squares task and, as such, they cannot be applied here in a direct manner. Therefore, the minimization problem of direct spectrum identification, formulated and solved in [24], required reformulation into the classical form of the linear least-squares problem. Next, the applicability of the generalized cross-validation and the L-curve method was examined and it was shown that these methods fail here. Attempts to transfer the ideas of GCV and L-curve methods to spectrum direct identification have also proved unsuccessful. Therefore, these commonly accepted methods of selecting the regularization parameter and their modifications are not suitable for direct spectrum identification. These studies are presented in Supplementary Material, ‘Section S.1. Applicability of GCV and L-Curve Methods to Direct Relaxation Spectrum Identification’.

Consequently, it was necessary to look for a new method of selecting the regularization parameter, specifically aimed at the problem of direct spectrum identification, which is the subject of this paper.

Two new multiplicative regularization indices are introduced as the products of the norm of the optimal spectra/the square of this norm and the square error of the relaxation modulus model. Based on the monotonicity of these indices, two problems of the regularization parameter optimal selection were stated and solved. The first and second order optimality conditions were derived and expressed by equations and inequalities in the matrix-vector form and, alternatively, in terms of finite series. A complete computational identification algorithm is presented. The results of simulation studies for short, middle, and long relaxation times polymers, modeled by Kohlrausch–Williams–Watts, and Gaussian-like spectra are presented.

In the previous article [24], the applicability of the direct identification method for different classes of real relaxation spectra was analyzed and the applicability limitations were pointed out. It was also demonstrated that the concept of direct relaxation spectrum identification can be applied both for viscoelastic fluids and viscoelastic solids; however, for solids, the respective modification described in [24] is necessary. Being aware of these issues, we do not discuss them in this article, instead referring the reader to [24].

In Supplementary Materials, in addition to the studies concerning the application of GCV and L-Curve methods, some detailed figures are presented. In Appendix A, derivations of some mathematical formulas are given. The main symbols and abbreviations are summarized in Appendix B.

## 2. Materials and Methods

In this section, we first briefly describe direct identification method introduced in [24] for relaxation spectra determination. We refer the reader to the source paper [24] for a more detailed description. All our considerations are illustrated on the examples of three simulated polymers, the rheological properties of which were described by the Gauss-like and Kohlrausch–Williams–Watts (KWW) relaxation spectra presented below, together with a description of the assumptions made for the simulated stress relaxation experiments.

### 2.1. Relaxation Spectra

In rheology [1,2,38], it is assumed that the linear relaxation modulus $G\left(t\right)$ (i.e., the stress per unit strain) has a relaxation spectrum integral representation given by:

(1) $$G\left(t\right)=\int_{0}^{\infty }\frac{H\left(\tau \right)}{\tau }e^{-t/\tau }d\tau ,$$

or equivalently by:

(2) $$G\left(t\right)=\int_{0}^{\infty }\frac{H\left(v\right)}{v}e^{-tv}dv,$$

where the continuous relaxation time $H\left(\tau \right)$ and frequency $H\left(v\right)$ spectra, related by equations:

(3) $$H\left(v\right)=H\left(\frac{1}{v}\right),\;\;H\left(\tau \right)=H\left(\frac{1}{\tau }\right),$$

characterize distributions of relaxation times τ and frequencies v. Although other definitions of the relaxation spectrum are known in the literature, for example, in [39,40,41], the definitions introduced by Equations (1) and (2) are dominant.

In the previous paper [24], following [33], the modified spectrum is introduced:

(4) $$H^{M}\left(v\right)=\frac{H\left(v\right)}{v},$$

where the upper index in $H^{M}\left(v\right)$ means ‘modified’. Therefore, Equation (2) can be rewritten as:

(5) $$G\left(t\right)=\int_{0}^{\infty }H^{M}\left(v\right)e^{-tv}dv,$$

which means that the relaxation modulus $G\left(t\right)$ is directly the Laplace transform of the spectrum $H^{M}\left(v\right)$.

### 2.2. Relaxation Spectra and Modulus Models

Assuming that $H^{M}\left(v\right)\in L^{2}\left(0,\right.\left.\;\infty \right)$, where $L^{2}\left(0,\right.\left.\;\infty \right)$ is the space of real-valued square-integrable functions on the interval $\left(0,\right.\left.\;\infty \right)$, the modified relaxation spectrum can be expressed as:

(6) $$H^{M}\left(v\right)=\sum_{k=0}^{\infty }g_{k}h_{k}\left(v\right),$$

where the basis functions are defined as follows:

(7) $$h_{k}\left(v\right)=e^{-\alpha kv},$$

with positive parameter α being a time-scaling factor expressed in seconds, while $g_{k}$ are constant model parameters. This is because the set of the linearly independent exponential functions $\left\{e^{-\alpha kv}\right\}$, k=0, 1,…, where α>0, i.e., the kernel of the Laplace transformation, form a basis of the space $L^{2}\left(0,\right.\left.\;\infty \right)$ [34]. Since the modified spectrum $H^{M}\left(v\right)\in L^{2}\left(0,\right.\left.\infty \right)$, therefore $H^{M}\left(v\right)\to 0$ as v→∞ and the first constant basis function can be neglected. Replacing the infinite summation in Equation (6) with a finite one of K first terms, the approximation of the modified spectrum $H^{M}\left(v\right)$ by a model of the form:

(8) $$H_{K}^{M}\left(v\right)=\sum_{k=1}^{K}g_{k}h_{k}\left(v\right),$$

is introduced, where the lower index of $H_{K}^{M}\left(v\right)$ is the number of model summands. Both the spectrum $H^{M}\left(v\right)$ (4) and model parameters $g_{k}$ are expressed in Pa·s.

By (4), the model of the original spectrum $H\left(v\right)$ related to (8) takes the form:

(9) $$H_{K}\left(v\right)=vH_{K}^{M}\left(v\right)=\sum_{k=1}^{K}g_{k}h_{k}\left(v\right)v.$$

By (5) and (8), the related relaxation modulus model is described by:

(10) $$G_{K}\left(t\right)=\int_{0}^{\infty }H_{K}^{M}\left(v\right)e^{-tv}dv=\sum_{k=1}^{K}g_{k}\frac{1}{t+\alpha k}=\sum_{k=1}^{K}g_{k}\phi_{k}\left(t\right),$$

where the basis functions, expressed in $s^{-1}$, are given by simple hyperbolic functions of time as follows [24]:

(11) $$\phi_{k}\left(t\right)=\frac{1}{t+\alpha k}.$$

**Figure 1:**
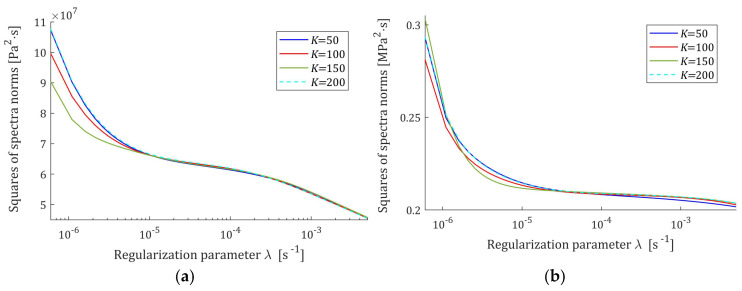
The norms $\|{\bar{H}}_{K}\left(\tau \right){\|}_{2}^{2}=\|{\bar{H}}_{K}^{M}\left(v\right){\|}_{2}^{2}$ of the optimal spectra models ${\bar{H}}_{K}^{M}\left(v\right)$ (28) and ${\bar{H}}_{K}\left(\tau \right)$ (30) as the functions of the regularization parameter λ for K=50, 100, 150, 200 relaxation modulus measurements disturbed by independent additive noises selected according uniform distribution on the interval $\left[-\xi ,\;\;\xi \right]$: (**a**) uni-mode Gauss spectrum $H\left(\tau \right)$ (32), ξ=10 Pa; (**b**) Kohlrausch–Williams–Watts (KWW) spectrum $H\left(\tau \right)$ (39) and ξ=0.5 kPa.

By the second equality in (3), the following model of the relaxation time spectrum corresponds to (9):

(12) $$H_{K}\left(\tau \right)=\sum_{k=1}^{K}g_{k}h_{k}\left(\tau \right),$$

where the basis functions:

(13) $$h_{k}\left(\tau \right)=e^{-\frac{\alpha k}{\tau }}\frac{1}{\tau },$$

and model parameters $g_{k}$ are identical with that of model (9). By (1) and (12), the relaxation modulus model is as follows:

(14) $$G_{K}\left(t\right)=\int_{0}^{\infty }\frac{H_{K}\left(\tau \right)}{\tau }e^{-t/\tau }d\tau =\sum_{k=1}^{K}g_{k}\int_{0}^{\infty }\frac{1}{\tau^{2}}e^{-\frac{t+\alpha k}{\tau }}d\tau =\sum_{k=1}^{K}g_{k}\phi_{k}\left(t\right),$$

where the basis functions $\phi_{k}\left(t\right)$ are given by (11), i.e., is identical with the model described by (10). The courses of the basis functions $h_{k}\left(v\right)$ (7), $h_{k}\left(v\right)v$, $h_{k}\left(\tau \right)$ (13) and $\phi_{k}\left(t\right)$ (11) are illustrated by Figures 1–4 in [24].

### 2.3. Identification

Model identification consists of selecting, within the given class of models, such a model, which ensures the best fit to the measurement results. For the relaxation spectra identification based on the time-domain experiment data, the mean square identification index related to the relaxation modulus measurements $\bar{G}\left(t_{k}\right)$ is usually used (compare [25,26,27,28]). In [24], an integral-square error between the real unknown modified spectrum and the spectrum model was taken as a measure of the model quality. This index was proved to be expressed in terms of the measurable relaxation modulus at uniquely defined sampling instants. Consequently, a new integral-empirical identification index was introduced in which the values of the real relaxation modulus are replaced by their noisy measurements. This index, basic for direct relaxation spectra identification, is considered in this paper.

Suppose that a stress relaxation experiment [2,42], performed on the specimen of the material under investigation, resulted in a set of measurements of the relaxation modulus $\left\{\bar{G}\left(t_{k}\right)=G\left(t_{k}\right)+z\left(t_{k}\right)\right\}$ at the sampling instants $t_{k}=\alpha k$, k=1,…,K; here, $z\left(t_{k}\right)$ is additive measurement noise.

The following integral square approximation index, equivalent for the relaxation time and modified frequency spectra, is introduced [24]:

(15) $$J\left(g_{K}\right)=\int_{0}^{\infty }{\left[H\left(\tau \right)-H_{K}\left(\tau \right)\right]}^{2}d\tau =\int_{0}^{\infty }{\left[H^{M}\left(v\right)-H_{K}^{M}\left(v\right)\right]}^{2}dv,$$

where $g_{K}={\left[\begin{array}{ccc} g_{1} & \cdots & g_{K} \end{array}\right]}^{T}$ is an K-element vector of the models (8)–(10) and (12) parameters; superscript “T” indicates transpose. Index $J\left(g_{K}\right)$ can be expressed as [24]:

(16) $$J\left(g_{K}\right)=\int_{0}^{\infty }H^{M}{\left(v\right)}^{2}dv-2\sum_{k=1}^{K}g_{k}G\left(\alpha k\right)+\sum_{k=1}^{K}\sum_{m=1}^{K}g_{k}g_{m}\;\varphi_{km},$$

where the coefficients:

(17) $$\varphi_{km}=\int_{0}^{\infty }h_{k}\left(v\right)h_{m}\left(v\right)dv=\int_{0}^{\infty }h_{k}\left(\tau \right)h_{m}\left(\tau \right)d\tau =\frac{1}{\alpha \left(k+m\right)},$$

are identical for the basis functions $h_{k}\left(v\right)$ and $h_{k}\left(\tau \right)$ of the models $H_{K}^{M}\left(v\right)$ (8) and $H_{K}\left(\tau \right)$ (12), respectively. The first summand of $J\left(g_{K}\right)$ is determined by the unknown relaxation spectrum, the second is affected by model parameters $g_{k}$ and the values of the relaxation modulus being measurable at the times $t_{k}=\alpha k$, and the last summand depends only on the times $t_{k}$ and model parameters.

By setting in (16) the measurements $\bar{G}\left(t_{k}\right)$ instead of the modulus $G\left(t_{k}\right)=G\left(\alpha k\right)$, the following integral-empirical index is introduced [24]:

(18) $${\bar{J}}_{K}\left(g_{K}\right)=\int_{0}^{\infty }H^{M}{\left(v\right)}^{2}dv-2\sum_{k=1}^{K}g_{k}\bar{G}\left(\alpha k\right)+\sum_{k=1}^{K}\sum_{m=1}^{K}g_{k}g_{m}\;\varphi_{km},$$

as a measure of the relaxation spectrum model error being linear-quadratic function of the models parameter $g_{K}$, which introducing vector-matrix notation:

(19) $$\Phi_{K}=\left[\begin{array}{ccc} \frac{1}{2} & \cdots & \frac{1}{K+1}\\ \vdots & \ddots & \vdots \\ \frac{1}{K+1} & \cdots & \frac{1}{2K} \end{array}\right],\;{\bar{G}}_{K}=\left[\begin{array}{c} \bar{G}\left(t_{1}\right)\\ \vdots \\ \bar{G}\left(t_{K}\right) \end{array}\right],$$

can be expressed in the form more useful for analysis and optimization purposes as follows:

(20) $${\bar{J}}_{K}\left(g_{K}\right)=\int_{0}^{\infty }H^{M}{\left(v\right)}^{2}dv-2{\bar{G}}_{K}^{T}\;g_{K}+\frac{1}{\alpha }g_{K}^{T}\;\Phi_{K}g_{K}.$$

According to Lemma 1 in [24], symmetric matrix $\Phi_{K}$ is positive definite for an arbitrary K≥1, therefore there exists a unique model parameter $g_{K}^{*}=\alpha \Phi_{K}^{-1}{\bar{G}}_{K}$ minimizing the empirical index ${\bar{J}}_{K}\left(g_{K}\right)$. Matrix $\Phi_{K}$, although of full-rank, is extremely ill-conditioned (for details, see [24]) and, when the data are noisy, even small changes in ${\bar{G}}_{K}$ would lead to an arbitrarily large artefact in the optimal $g_{K}^{*}$. Therefore, in [24], Tikhonov regularization [43] is used, which, when applied to ${\bar{J}}_{K}\left(g_{K}\right)$ (20), strives to minimize a modified linear-quadratic function of the form:

(21) $$\underset{g_{K}\in R^{K}}{min}\frac{1}{\alpha }g_{K}^{T}\;\Phi_{K}g_{K}-2{\bar{G}}_{K}^{T}\;g_{K}+\lambda g_{K}^{T}\;g_{K},$$

where λ>0 is a regularization parameter expressed in $s^{-1}$. The first summand of (20), being independent of $g_{K}$, does not have to be taken into account here, just as it did not affect the minimization result of the original index (20). The problem (21) is well-posed; that is the solution exists, is unique, and is given by the formula:

(22) $${\bar{g}}_{K}\left(\lambda \right)=\alpha {\left(\Phi_{K}+\alpha \lambda I_{K}\right)}^{-1}{\bar{G}}_{K},$$

where $I_{K}$ is K×K identity matrix. The optimal regularized model parameter vector ${\bar{g}}_{K}\left(\lambda \right)$ continuously depends on both the matrix $\Phi_{K}$ and the measurements ${\bar{G}}_{K}$. Here and hereafter, the notations of those variables that depend on the measurements $\bar{G}\left(t_{k}\right)$ are highlighted with the bars, cf., ${\bar{J}}_{K}\left(g_{K}\right)$ (18) and ${\bar{g}}_{K}\left(\lambda \right)$ above.

### 2.4. Algebraic Details

The Formula (22), however elegant and compact, is of little use for computational purposes. Therefore, in [24], the technique of singular decomposition was used, one of the greatest results of linear algebra, the applications of which are discussed by many authors, e.g., [44]. Generally, the singular value decomposition (SVD) of a matrix is the factorization of the matrix into the product of three matrices: two matrices where the columns are orthonormal and a diagonal matrix with nonnegative real matrix singular values in descending order on the main diagonal [44,45].

The singular value decomposition of symmetric matrix $\Phi_{K}$ (19) is the factorization of $\Phi_{K}$ into the product [24]:

(23) $$\Phi_{K}=U_{K}\Sigma_{K}U_{K}^{T},$$

where the diagonal K×K matrix:

(24) $$\Sigma_{K}=diag\left(\sigma_{1},\ldots ,\sigma_{K}\right),$$

is composed of the singular values $\sigma_{1}\geq \ldots \geq \sigma_{k}\geq \ldots \geq \sigma_{K}$ of $\Phi_{K}$ being positive (cf., Lemma 1 in [24]) and matrix $U_{K}\in R^{K,K}$ is orthogonal.

This phenomenal algebraic tool is applied here because, thanks to the diagonalization of $\Phi_{K}$ and the orthogonality of $U_{K}$, it simplifies the calculation of the algorithm and facilitates its analytical analysis. The diagonality of $\Sigma_{K}$, and mainly the orthogonality of $U_{K}$, will be repeatedly used.

Taking advantage of the diagonal structure of $\Sigma_{K}$ and orthogonality of $U_{K}$, we can express ${\bar{g}}_{K}\left(\lambda \right)$ (22) as:

(25) $${\bar{g}}_{K}\left(\lambda \right)=\alpha U_{K}{\left(\Sigma_{K}+\alpha \lambda I_{K}\right)}^{-1}Y_{K}=\alpha U_{K}\Omega_{K}\left(\lambda \right)Y_{K},$$

where diagonal K×K matrix function of the regularization parameter:

(26) $$\Omega_{K}\left(\lambda \right)={\left(\Sigma_{K}+\alpha \lambda I_{K}\right)}^{-1}=diag\left(\frac{1}{\sigma_{1}+\alpha \lambda },\ldots ,\frac{1}{\sigma_{K}+\alpha \lambda }\right),$$

and K dimensional vector:

(27) $$Y_{K}=U_{K}^{T}{\bar{G}}_{K}.$$

### 2.5. Optimal Models and Their Properties

According to (8), (9), and (12), the resulting best relaxation spectra models are as follows:

(28) $${\bar{H}}_{K}^{M}\left(v\right)=\sum_{k=1}^{K}{\bar{g}}_{k}\left(\lambda \right)h_{k}\left(v\right),$$

(29) $${\bar{H}}_{K}\left(v\right)=\sum_{k=1}^{K}{\bar{g}}_{k}\left(\lambda \right)h_{k}\left(v\right)v,$$

and:

(30) $${\bar{H}}_{K}\left(\tau \right)=\sum_{k=1}^{K}{\bar{g}}_{k}\left(\lambda \right)h_{k}\left(\tau \right),$$

where ${\bar{g}}_{k}\left(\lambda \right)$ are elements of the vector ${\bar{g}}_{K}\left(\lambda \right)$ (22); equivalently, (25).

The optimal approximation of the spectrum $H\left(\tau \right)$ by model ${\bar{H}}_{K}\left(\tau \right)$ and, equivalently, optimal approximation of the modified spectrum $H^{M}\left(v\right)$ (4) by the series of functions ${\bar{H}}_{K}^{M}\left(v\right)$ (28), in view of (14) and (10), means that the corresponding relaxation modulus model is described by:

(31) $${\bar{G}}_{K}\left(t\right)=\sum_{k=1}^{K}{\bar{g}}_{k}\left(\lambda \right)\phi_{k}\left(t\right),$$

with the basis functions $\phi_{k}\left(t\right)$ (11).

In [24], the model smoothness and its accuracy for noisy measurements of the relaxation modulus have been analyzed. It was proved that both the norms of the spectra models and the accuracy of the relaxation modulus approximation depend on the real relaxation modulus $G\left(t_{k}\right)$ and measurement noises $z\left(t_{k}\right)$ affecting the vector $Y_{K}$ (27), the scaling factor α, singular values of the matrix $\Phi_{K}$ (19), and is affected by the regularization parameter λ; for details see Propositions 1 and 2 in [24]. The monotonicity of the square norms of the optimal models ${\bar{H}}_{K}^{M}\left(v\right)$ and ${\bar{H}}_{K}\left(\tau \right)$ and other quality indices as a functions of the regularization parameter is examined below.

### 2.6. Short, Midlle and Long Relaxation Times Spectra

We begin with three examples of the relaxation spectra used in polymers rheology—short relaxation time Kohlrausch–Williams–Watts (KWW) spectrum, and the uni- and double-mode Gauss-like spectra having middle and long relaxation times, respectively.

The stretched exponential relaxation of the KWW model has been found to be more appropriate than standard exponentials of the classic Maxwell model to describe viscoelastic properties of many polymers, e.g., the segmental dynamics and the glass transition behavior of poly(2-vinylpyridine) [46], polymer melts [47], relaxation of bone and bone collagen [48], and alginate films while considering glycerol concentration [49].

**Figure 2:**
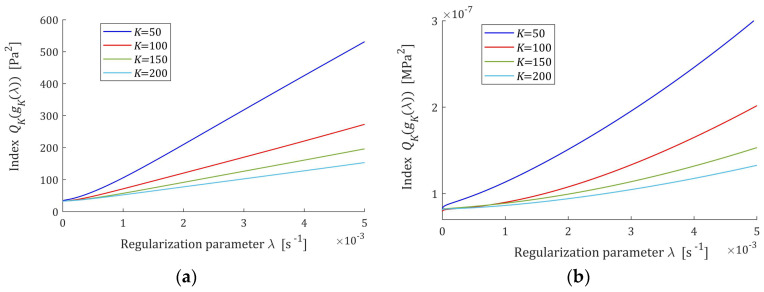
The mean square error $Q_{K}\left({\bar{g}}_{K}\left(\lambda \right)\right)$ (45) of the relaxation modulus model ${\bar{G}}_{K}\left(t\right)$ (31) as the function of the regularization parameter λ for K=50, 100, 150, 200 relaxation modulus measurements disturbed by independent additive noises selected according uniform distribution on the interval $\left[-\xi ,\;\;\xi \right]$: (**a**) uni-mode Gauss spectrum $H\left(\tau \right)$ (32), ξ=10 Pa; (**b**) KWW spectrum ξ=10 Pa (39) and ξ=10 kPa In the description of the vertical axes, here and in the following figures, the line (bar) above the optimal parameter ${\bar{g}}_{K}\left(\lambda \right)$ has been omitted to make the description of the figures more readable.

**Figure 3:**
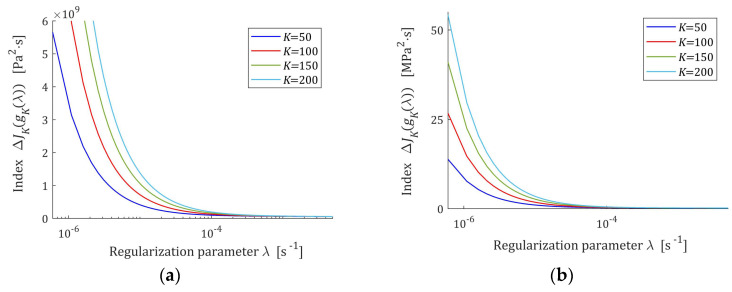
The increment $∆{\bar{J}}_{K}\left({\bar{g}}_{K}\left(\lambda \right)\right)$ (49) of the integral-empirical index ${\bar{J}}_{K}\left({\bar{g}}_{K}\left(\lambda \right)\right)$ (48) as the function of the regularization parameter λ for K=50, 100, 150, 200 relaxation modulus measurements disturbed by independent additive noises selected according uniform distribution on the interval $\left[-\xi ,\;\;\xi \right]$: (**a**) uni-mode Gauss spectrum $H\left(\tau \right)$ (32), ξ=10 Pa; (**b**) KWW spectrum $H\left(\tau \right)$ (39) and ξ=0.5 kPa.

Distributions of the Gauss-like relaxation spectra were studied while investigating new identification methods, for example in [14] (Figures 9, 11 and 17), [20] (Figure 2), and [50] (Figures 2, 3, 6–11 and 14). The Gaussian-like relaxation spectra were used for modelling the viscoelastic properties of several real polymers, e.g., polyacrylamide gels [51] (Figure A4) with exemplary applications in food industry [52], poly(methyl methacrylate) [53], and also polyethylene [54] and carboxymethylcellulose (CMC) [55], which are used in food technology [56,57,58]. Similarly, the spectra of various biopolymers important for food technology are Gaussian in nature, for example, fresh egg white-hydrocolloids [55], xanthan gum water solution [52], cold gel-like emulsions stabilized with bovine gelatin [59], and some (potato, corn, wheat, and banana) native starch gels [60].

The stress relaxation experiment conditions are also characterized.

#### 2.6.1. Uni-Mode Gauss-like Spectrum

Consider the uni-modal Gauss-like distribution:

(32) $$H\left(\tau \right)=\vartheta e^{-{\left(\frac{1}{\tau }-m\right)}^{2}/q}/\tau ,$$

where the parameters are as follows [23,24,25]: ϑ=31.52 kPa·s, $m=0.0912{\;s}^{-1}$, and $q=3.25\times 10^{-3}{\;s}^{-2}$. The respective relaxation modulus is as follows [23,24]:

(33) $$G\left(t\right)=\frac{\sqrt{\pi q}}{2}\vartheta \;e^{\frac{1}{4}t^{2}q-mt}erfc\left(\frac{\frac{1}{2}tq-m}{\sqrt{q}}\right),$$

with the complementary error function $erfc\left(x\right)$ defined by [61] (Equation (8.250.4)):

$$erfc\left(x\right)=\frac{2}{\sqrt{\pi \;}\;}\int_{x}^{\infty }e^{-z^{2}}dz.$$

By (3), the corresponding relaxation frequency spectrum is given by:

(34) $$H\left(v\right)=\vartheta ve^{-{\left(v-m\right)}^{2}/q},$$

and, in view of (4), the modified spectrum is described by:

(35) $$H^{M}\left(v\right)=\vartheta e^{-{\left(v-m\right)}^{2}/q}.$$

In [24], analytical formulas were derived describing the square norms of the spectra $H\left(\tau \right)$ (32), $H\left(v\right)$ (34) and $H^{M}\left(v\right)$ (35), which are as follows: $\|H^{M}\left(v\right){\|}_{2}=\|H\left(\tau \right){\|}_{2}=8.422432\;\mathrm{kPa}\cdot s^{1/2}$ and $\|H\left(v\right){\|}_{2}=0.805043\;\mathrm{kPa}\cdot s^{-1/2}$. Here $\|\cdot {\|}_{2}$ denotes the square norm in the space $L^{2}\left(0,\right.\left.\infty \right)$ and, simultaneously, the square vector norm in the real Euclidean space $R^{K}$.

Following [24], the preliminary relaxation test experiment was performed and the modulus $G\left(t\right)$ (33) measurements were recorded for 200 s. Then, for any K, the time scale factors α were selected by comparing the courses of experimentally recorded $\left\{\bar{G}\left(t_{i}\right)\right\}$ and basis functions $\phi_{k}\left(t\right)$ (11) for a few 1≤k≤K. Subsequently, to simulate the experiment, K sampling times $t_{k}=\alpha k$ were generated with the period α for K=20, 50, 100, 150, 200 measurements. Additive measurement noises $z\left(t_{k}\right)$ were selected independently by random choice with uniform distribution on the interval $\left[-10,\;10\right]\;\mathrm{Pa}$. The measurements $\bar{G}\left(t_{k}\right)$ were recorded.

#### 2.6.2. Double-Mode Gauss-like Spectrum

The double-mode Gauss-like relaxation spectrum considered in [23,24,25,26,54] is described by:

(36) $$H\left(\tau \right)=\left[\vartheta_{1}e^{-{\left(\frac{1}{\tau }-m_{1}\right)}^{2}/q_{1}}+\vartheta_{2}e^{-{\left(\frac{1}{\tau }-m_{2}\right)}^{2}/q_{2}}\right]/\tau ,$$

where the parameters are as follows [24,25,26]: $\vartheta_{1}=467\;\mathrm{Pa}\cdot s$, $m_{1}=0.0037{\;s}^{-1}$, $q_{1}=1.124261\times 10^{-6}{\;s}^{-2}$, $\vartheta_{2}=39\;\mathrm{Pa}\cdot s$, $m_{2}=0.045{\;s}^{-1}$, and $q_{2}=1.173\times 10^{-3}{\;s}^{-2}$. The corresponding relaxation frequency spectrum is given by:

(37) $$H\left(v\right)=\vartheta_{1}ve^{-{\left(v-m_{1}\right)}^{2}/q_{1}}+\vartheta_{2}ve^{-{\left(v-m_{2}\right)}^{2}/q_{2}}.$$

The related real relaxation modulus is composed of two summands of (33) form. In [24], the analytical formulas are derived to describe the square norms of the ‘real’ spectra $H\left(v\right)$ (37), $H\left(\tau \right)$ (36) and $H^{M}\left(v\right)=H\left(v\right)/v$, being as follows: $\|H^{M}\left(v\right){\|}_{2}=\|H\left(\tau \right){\|}_{2}=19.257051\;\mathrm{Pa}\cdot s^{1/2}$ and $\|H\left(v\right){\|}_{2}=0.394490\;\mathrm{Pa}\cdot s^{-1/2}$.

Following [24,25,26], in the preliminary experiment, N=5000 sampling instants were generated with the constant period in the time interval $T=\left[0,\;1550\right]$ seconds. Next, the time-scale factors were selected for K=50, 100, 150, 200, 300. Following [24,25,26], additive measurement noises $z\left(t_{i}\right)$ were selected independently by random choice with uniform distribution on the interval $\left[-0.005,\;0.005\right]\;\mathrm{Pa}$. Then, the noise measurements $\bar{G}\left(t_{k}\right)$ were recorded for K=50, 100, 150, 200, 300.

#### 2.6.3. KWW Relaxation Spectrum

The uni-modal [62] relaxation spectrum of the KWW model [63]:

(38) $$G\left(t\right)=G_{0}e^{-{\left(\frac{t}{\tau_{r}}\right)}^{\beta }},$$

is described by the infinite series [62,63]:

$$H\left(\tau \right)=\frac{G_{0}}{\pi }\;\sum_{k=1}^{\infty }\frac{{\left(-1\right)}^{k+1}}{k!}\sin\left(\pi \beta k\right)\;\Gamma \left(\beta k+1\right)\;{\left(\frac{\tau }{\tau_{r}}\right)}^{\beta k},$$

where $\Gamma \left(n\right)$ is Euler’s gamma function [61] (Equation (8.310.1)), the stretching exponent 0<β<1, $\tau_{r}$ is the relaxation time, and $G_{0}$ is the initial shear modulus. For the stretching exponent β=0.5, spectrum $H\left(\tau \right)$ has simple analytical form [24,62]:

(39) $$H\left(\tau \right)=\frac{G_{0}}{2\sqrt{\pi }}\sqrt{\frac{\tau }{\tau_{r}}}\;e^{-\frac{\tau }{4\tau_{r}\;}},$$

owing to which it is assumed here following [24]. The exponent β=0.5 has been reported by Plazek and Ngai [64] for poly(methylphenylsiloxane) at the glass temperature $T_{g}=200\;K$. The coefficient β=0.5 has been obtained for dehydroabietic acid, sorbitol, BBKDE, 1,4-cis-polyisoprene, and silicate flint glass [65] (Table I). The exponent β near 0.5 has been found for BCDE (β=0.51), toluene (25%) (β=0.52), and a few other forms of glass. Unfortunately, the related relaxation times $\tau_{r}$ are not reported in [64,65]. Recently, Chen et al. [66] applied the KWW model to crosslinked polystyrene with the following parameters: $G_{0}=0.78\;\mathrm{MPa}$, β=0.59, and $\tau_{r}=1.08\;s$ [66] (Table 4). Owing to small mean least squares error (1.2853 × 10^−4^ MPa [24]) between the moduli $G\left(t\right)$ (38) for β=0.5 and β=0.59, obtained for 500 equidistant sampling points, and having in mind Figure 13 in [24], which illustrates the discrepancy between these moduli, for the purpose of our studies we replace β=0.59 by β=0.5.

The relaxation frequency spectra corresponding to (39) are as follows:

(40) $$H\left(v\right)=\frac{G_{0}}{2\sqrt{\pi }}\sqrt{\frac{1}{\tau_{r}\cdot v}}\;e^{-\frac{1}{4\;\tau_{r}\cdot v}},$$

and

(41) $$H^{M}\left(v\right)=\frac{G_{0}}{2\sqrt{\pi }}\sqrt{\frac{1}{\tau_{r}\cdot v^{3}}}\;e^{-\frac{1}{4\;\tau_{r}\cdot v}}.$$

For the preliminary experiment, following [24], N=500 sampling instants were generated with the constant period in the interval $T=\left[0,\;50\right]$ seconds. Noises $z\left(t_{i}\right)$ were selected by independent random choice with uniform distribution on the interval $\left[-0.5,\;0.5\right]\;\mathrm{kPa}$.

## 3. Results and Discussion

Both the spectra model’s smoothness and the errors of the spectra and modulus models depend on the value of the regularization parameter. Therefore, the appropriate choice of respective regularization parameter is important to identify the best model. In [24], a simple rough rule based on the spectral conditional number of the matrix $\left(\Phi_{K}+\alpha \lambda I_{K,K}\right)$, being fundamental for the optimal model parameter ${\bar{g}}_{K}\left(\lambda \right)$ (22), is proposed and examined.

However, there are well-studied techniques for computing a good regularization parameter, such as generalized cross-validation and the L-curve technique. These methods have been developed for classical least-squares task and, as such, they cannot be directly applied here. Therefore, the regularized minimization problem (21) should be reformulated to the classic form of the linear least-squares problem, which was carried out, based on the properties of the non-singular matrix $\Phi_{K}$, in Section S.1.1. ‘Direct Spectrum Identification as a Least-Squares Problem’ in the Supplementary Materials. In Section S.1.2 ‘Applicability of the Generalized Cross Validation’, the applicability of the generalized cross-validation [35,36] has been considered. Unfortunately, as shown, the GCV method is not applicable here, since the GCV function is monotonically decreasing. Therefore, in Section S.1.3 ‘GCV Idea Applied to Direct Spectrum Identification Task’, the idea of the GCV method is applied directly to direct spectrum identification. However, it is demonstrated that such an approach also failed; the respective modified GCV function monotonically increases. In summary, both the classic GCV and modified GCV method are not applicable for direct spectrum identification.

Applicability of the L-curve method [37] is examined in Section S.1.4 ‘Applicability of the L-Curve Method’ for direct spectrum identification formulated as the classic least-squares problem. Next, the idea of the L-Curve method is applied to the task of direct spectra identification in a direct manner, which means the tradeoff-curve between the square norms of the relaxation modulus error and optimal model parameter. This is described in Section S.1.5 ‘Applicability of the L-Curve Idea to Direct Spectrum Identification’. This widely accepted method of selecting the regularization parameter also fails with respect to our task.

Therefore, another method of selecting the regularization parameter, specifically addressed to the problem of direct spectrum identification, must be sought. For this purpose, we first examine the properties of the main model quality indices, specifically the square norms of optimal spectra models and their parameter, the integral-empirical spectrum approximation index, and relaxation modulus model error, as the functions of λ to better explain the effect of the regularization parameter.

Next, two new regularization indices are introduced as the products of the norm of optimal spectra models/square of this norm and the square error of the relaxation modulus model. Based on the monotonicity of these indices, two problems of the optimal regularization parameter selection are stated and solved. The first and second order optimality conditions are derived.

Finally, a computational identification algorithm is developed and the results of simulation studies for Kohlrausch–Williams–Watts and Gaussian-like spectra are presented.

### 3.1. Monotonicity of the Models Quality Indices

The integral square norms $\|{\bar{H}}_{K}^{M}\left(v\right){\|}_{2}$ and $\|{\bar{H}}_{K}\left(\tau \right){\|}_{2}$ are natural measures of the model’s smoothness. For the models ${\bar{H}}_{K}^{M}\left(v\right)$ (28) and ${\bar{H}}_{K}\left(\tau \right)$ (30), we have (cf., Proposition 1 in [24]):

(42) $$\|{\bar{H}}_{K}^{M}\left(v\right){\|}_{2}^{2}=\|{\bar{H}}_{K}\left(\tau \right){\|}_{2}^{2}=\sum_{k=1}^{K}\sum_{m=1}^{K}{\bar{g}}_{k}\left(\lambda \right)g_{m}\varphi_{km}=\frac{1}{\alpha }{\bar{g}}_{K}^{T}\left(\lambda \right)\Phi_{K}{\bar{g}}_{K}\left(\lambda \right),$$

which, in view of (25), (23), the orthogonality of $U_{K}$ and diagonal structure of the matrices $\Omega_{K}\left(\lambda \right)$ (26), and $\Sigma_{K}$ (24), is given by:

(43) $$\|{\bar{H}}_{K}^{M}\left(v\right){\|}_{2}^{2}=\|{\bar{H}}_{K}\left(\tau \right){\|}_{2}^{2}=\alpha Y_{K}^{T}\Omega_{K}\left(\lambda \right)\Sigma_{K}\Omega_{K}\left(\lambda \right)Y_{K}=\alpha \sum_{k=1}^{K}\;\frac{\sigma_{k}y_{k}^{2}}{{\left(\sigma_{k}+\alpha \lambda \right)}^{2}},$$

where $y_{k}$, k=1,…,K, are the elements of the vector $Y_{K}$ (27). Therefore, the greater the parameter λ, the more limited the fluctuations of the models ${\bar{H}}_{K}^{M}\left(v\right)$ and ${\bar{H}}_{K}\left(\tau \right)$ are. By (43), we have:

(44) $$\frac{\partial \|{\bar{H}}_{K}\left(\tau \right){\|}_{2}^{2}}{\partial \lambda }=-2\alpha^{2}\sum_{k=1}^{K}\;\frac{\sigma_{k}y_{k}^{2}}{{\left(\sigma_{k}+\alpha \lambda \right)}^{3}},$$

and next:

$$\frac{\partial^{2}\|{\bar{H}}_{K}\left(\tau \right){\|}_{2}^{2}}{\partial \lambda^{2}}=6\alpha^{3}\sum_{k=1}^{K}\;\frac{\sigma_{k}y_{k}^{2}}{{\left(\sigma_{k}+\alpha \lambda \right)}^{4}}.$$

Therefore, the squares of the norms $\|{\bar{H}}_{K}\left(\tau \right){\|}_{2}^{2}=\|{\bar{H}}_{K}^{M}{\left(v\right)\|}_{2}^{2}$ are convex monotonically decreasing functions of λ, the exemplary courses of which are illustrated in Figure 1 for the Gauss-like $H\left(\tau \right)$ (32) and KWW $H\left(\tau \right)$ (39) spectra. Logarithmic scales are applied for the horizontal axes.

In turn, only the product αλ, not α and λ independently, affect the mean square error of the relaxation modulus model ${\bar{G}}_{K}\left(t\right)$ (31), being given by [24]:

(45) $$Q_{K}\left({\bar{g}}_{K}\left(\lambda \right)\right)=\frac{1}{K}\sum_{k=1}^{K}{\left[\bar{G}\left(t_{k}\right)-{\bar{G}}_{K}\left(t_{k}\right)\right]}^{2}.$$

According to Proposition 4 in [24], this index can be expressed as follows:

(46) $$Q_{K}\left({\bar{g}}_{K}\left(\lambda \right)\right)=\frac{1}{K}{\left[{\bar{G}}_{K}-\frac{1}{\alpha }\Phi_{K}{\bar{g}}_{K}\left(\lambda \right)\right]}^{T}\left[{\bar{G}}_{K}-\frac{1}{\alpha }\Phi_{K}{\bar{g}}_{K}\left(\lambda \right)\right]=\frac{1}{K}\sum_{k=1}^{K}\frac{y_{k}^{2}\;{\left(\alpha \lambda \right)}^{2}}{{\left(\sigma_{k}+\alpha \lambda \right)}^{2}},$$

whence:

(47) $$\frac{\partial Q_{K}\left({\bar{g}}_{K}\left(\lambda \right)\right)}{\partial \lambda }=\frac{2}{K}\alpha^{2}\lambda \sum_{k=1}^{K}\frac{y_{k}^{2}\sigma_{k}}{{\left(\sigma_{k}+\alpha \lambda \right)}^{3}},$$

and:

$$\frac{\partial^{2}Q_{K}\left({\bar{g}}_{K}\left(\lambda \right)\right)}{\partial \lambda^{2}}=\frac{2}{K}\alpha^{2}\left[\sum_{k=1}^{K}\frac{y_{k}^{2}\sigma_{k}\left(\sigma_{k}-2\alpha \lambda \right)}{{\left(\sigma_{k}+\alpha \lambda \right)}^{4}}\right].$$

Therefore, the error of the relaxation modulus model grows with increasing regularization parameter λ, and is convex for very small αλ and concave for large αλ. Figure 2 shows the courses of $Q_{K}\left({\bar{g}}_{K}\left(\lambda \right)\right)$ for the Gauss-like and KWW spectra.

By SVD (23), due to the diagonal structure of the matrices $\Omega_{K}\left(\lambda \right)$ (26) and $\Sigma_{K}$ (24), having in mind the notation (27), the integral-empirical index ${\bar{J}}_{K}\left(g_{K}\right)$ (20) for the optimal relaxation spectra models with the parameter ${\bar{g}}_{K}\left(\lambda \right)$ (22) can be written as:

(48) $${\bar{J}}_{K}\left({\bar{g}}_{K}\left(\lambda \right)\right)=\int_{0}^{\infty }H^{M}{\left(v\right)}^{2}dv-\alpha \sum_{k=1}^{K}\frac{y_{k}^{2}\;\left(\sigma_{k}+2\alpha \lambda \right)}{{\left(\sigma_{k}+\alpha \lambda \right)}^{2}},$$

where the first summand of the right-hand side $\int_{0}^{\infty }H^{M}{\left(v\right)}^{2}dv=\int_{0}^{\infty }H{\left(\tau \right)}^{2}d\tau$ depends on the unknown relaxation spectrum of the real material. Since, the first derivative:

$$\frac{\partial {\bar{J}}_{K}\left({\bar{g}}_{K}\left(\lambda \right)\right)}{\partial \lambda }=2\alpha^{3}\sum_{k=1}^{K}y_{k}^{2}\frac{\lambda }{{\left(\sigma_{k}+\alpha \lambda \right)}^{3}},$$

is positive for any λ>0, the error of the relaxation spectra models grows with increasing regularization parameter λ. Simultaneously:

$$\frac{\partial^{2}{\bar{J}}_{K}\left({\bar{g}}_{K}\left(\lambda \right)\right)}{\partial \lambda^{2}}=2\alpha^{3}\sum_{k=1}^{K}y_{k}^{2}\frac{\left(\sigma_{k}-\alpha \lambda \right)}{{\left(\sigma_{k}+\alpha \lambda \right)}^{4}}.$$

Therefore, ${\bar{J}}_{K}\left({\bar{g}}_{K}\left(\lambda \right)\right)$ is convex function of λ for very small λ and concave for large λ. For λ→∞, index ${\bar{J}}_{K}\left({\bar{g}}_{K}\left(\lambda \right)\right)$ tends to the squares of spectra norms $\int_{0}^{\infty }H^{M}{\left(v\right)}^{2}dv=\|H\left(\tau \right){\|}_{2}^{2}=\|H^{M}\left(v\right){\|}_{2}^{2}$.

Since ${\bar{J}}_{K}\left({\bar{g}}_{K}\left(\lambda \right)\right)$ depends on the norm of the real unknown spectrum, for further analysis it is convenient to use the increment of this index given by:

(49) $$∆{\bar{J}}_{K}\left({\bar{g}}_{K}\left(\lambda \right)\right)=\alpha \sum_{k=1}^{K}\frac{y_{k}^{2}\;\left(\sigma_{k}+2\alpha \lambda \right)}{{\left(\sigma_{k}+\alpha \lambda \right)}^{2}},$$

which, in view of the above analysis, is a monotonically decreasing function of λ, the courses of which for the Gauss-like and KWW spectra are plotted in Figure 3.

**Figure 4:**
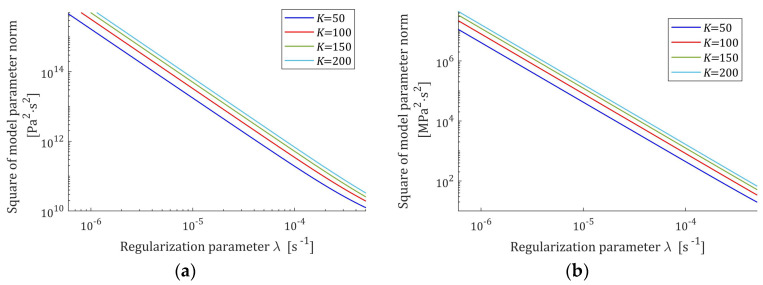
The square of the optimal model parameter norm $\|{\bar{g}}_{K}\left(\lambda \right){\|}_{2}^{2}$ (50) as the function of the regularization parameter λ for K=50, 100, 150, 200 relaxation modulus measurements disturbed by independent additive noises selected according uniform distribution on the interval $\left[-\xi ,\;\;\xi \right]$: (**a**) uni-mode Gauss-like relaxation spectrum $H\left(\tau \right)$ (32), ξ=10 Pa; (**b**) KWW spectrum $H\left(\tau \right)$ (39), ξ=0.5 kPa.

In view of (25), (26), and (27), the square norm of the regularized model parameter vector is as follows:

(50) $$\|{\bar{g}}_{K}\left(\lambda \right){\|}_{2}^{2}={\bar{g}}_{K}^{T}\left(\lambda \right){\bar{g}}_{K}\left(\lambda \right)=\alpha^{2}\sum_{k=1}^{K}\;\frac{y_{k}^{2}}{{\left(\sigma_{k}+\alpha \lambda \right)}^{2}},$$

i.e., is monotonically decreasing convex function of λ. The courses of this norm for the Gauss-like and KWW spectra are plotted in Figure 4.

From the above monotonicity analysis, the following rule results: the better smoothing of the spectra models and their parameters vector, the worse the fit indices of the spectra and relaxation modulus models. Although minimizing the additive weighted index ${\bar{J}}_{K}\left(g_{K}\right)$ (20) in the optimization task (21) is, in fact, a search for a compromise between a good fit of the relaxation spectra models and the smoothness of the model parameters vector, there is still a conflict between the fit of the spectra and relaxation modulus models and the smoothing of the spectra and their parameters despite the optimal selection of the parameter’s vector. An appropriate optimal selection of the regularization parameter will provide another, deeper, compromise between these conflicting requirements.

Additionally, comparing (50) and (46), we see that:

(51) $$Q_{K}\left({\bar{g}}_{K}\left(\lambda \right)\right)=\frac{\lambda^{2}}{K}\|{\bar{g}}_{K}\left(\lambda \right){\|}_{2}^{2},$$

whence:

$$\frac{Q_{K}\left({\bar{g}}_{K}\left(\lambda \right)\right)}{\|{\bar{g}}_{K}\left(\lambda \right){\|}_{2}^{2}}=\frac{\lambda^{2}}{K},$$

which means that the larger the λ, the faster the modulus error $Q_{K}\left({\bar{g}}_{K}\left(\lambda \right)\right)$ (51) grows with the decay of the norm $\|{\bar{g}}_{K}\left(\lambda \right){\|}_{2}^{2}$.

In turn, Equations (43), (49), and (50) imply the relation:

$$∆{\bar{J}}_{K}\left({\bar{g}}_{K}\left(\lambda \right)\right)=\|{\bar{H}}_{K}\left(\tau \right){\|}_{2}^{2}+2\lambda \|{\bar{g}}_{K}\left(\lambda \right){\|}_{2}^{2},$$

where the spectrum norm and $∆{\bar{J}}_{K}\left({\bar{g}}_{K}\left(\lambda \right)\right)$ decrease with increasing λ, while the last term $\lambda \|{\bar{g}}_{K}\left(\lambda \right){\|}_{2}^{2}$ increases for small λ and decreases for large λ.

### 3.2. New Multiplicative Indices

In the optimization task (21), the integral-empirical index ${\bar{J}}_{K}\left(g_{K}\right)$ (20) is minimized with the regularizing quadratic term $g_{K}^{T}\;g_{K}=\|g_{K}{\|}_{2}^{2}$ related to the model parameter. In result, the model parameter $g_{K}$ is smoothed; however, the spectra models are only indirectly smoothed. The integral-empirical error of the spectra models is minimized, but the error of the relaxation modulus model is only indirectly reduced. Therefore, for the choice of the best regularization parameter, the norms $\|{\bar{H}}_{K}^{M}\left(v\right){\|}_{2}^{2}=\|{\bar{H}}_{K}\left(\tau \right){\|}_{2}^{2}$ of the optimal spectra models and the mean square error $Q_{K}\left({\bar{g}}_{K}\left(\lambda \right)\right)$ (45) of the relaxation modulus model ${\bar{G}}_{K}\left(t\right)$ (31) are taken into account.

Let us define two new multiplicative indices: the Multiplicative Regularization Index (MRI):

(52) $$G_{K}\left(\lambda \right)=\|{\bar{H}}_{K}\left(\tau \right){\|}_{2}\cdot Q_{K}\left({\bar{g}}_{K}\left(\lambda \right)\right)=\|{\bar{H}}_{K}^{M}\left(v\right){\|}_{2}\cdot Q_{K}\left({\bar{g}}_{K}\left(\lambda \right)\right)$$

and the Square Multiplicative Regularization Index (SMRI):

(53) $$J_{K}\left(\lambda \right)=\|{\bar{H}}_{K}\left(\tau \right){\|}_{2}^{2}\cdot Q_{K}\left({\bar{g}}_{K}\left(\lambda \right)\right)=\|{\bar{H}}_{K}^{M}\left(v\right){\|}_{2}^{2}\cdot Q_{K}\left({\bar{g}}_{K}\left(\lambda \right)\right),$$

which, by (43) and (46), are given by the following functions of the regularization parameter:

(54) $$G_{K}\left(\lambda \right)=\frac{1}{K}\sqrt{\sum_{k=1}^{K}\;\frac{\alpha \sigma_{k}y_{k}^{2}}{{\left(\sigma_{k}+\alpha \lambda \right)}^{2}}}\left[\sum_{k=1}^{K}\frac{y_{k}^{2}\;{\left(\alpha \lambda \right)}^{2}}{{\left(\sigma_{k}+\alpha \lambda \right)}^{2}}\right],$$

and:

(55) $$J_{K}\left(\lambda \right)=\frac{1}{K}\left[\sum_{k=1}^{K}\;\frac{\alpha \sigma_{k}y_{k}^{2}}{{\left(\sigma_{k}+\alpha \lambda \right)}^{2}}\right]\left[\sum_{k=1}^{K}\frac{y_{k}^{2}\;{\left(\alpha \lambda \right)}^{2}}{{\left(\sigma_{k}+\alpha \lambda \right)}^{2}}\right].$$

**Figure 5:**
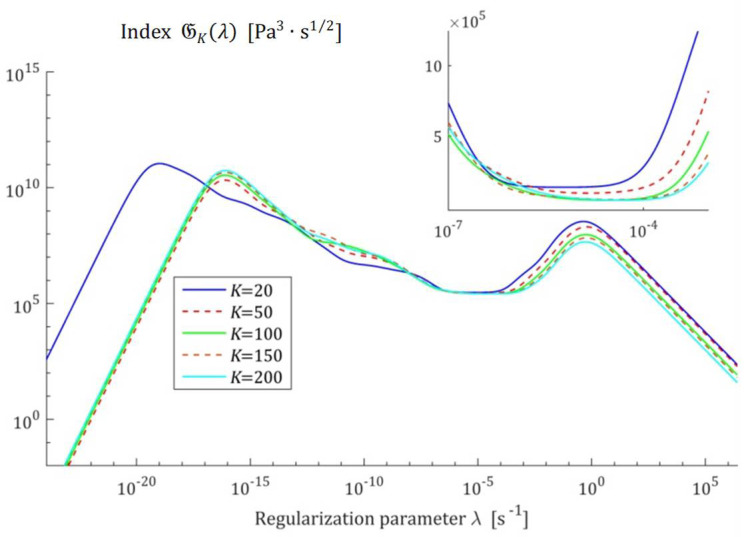
Multiplicative Regularization Index (MRI) $G_{K}\left(\lambda \right)$ (52) as the function of the regularization parameter λ for K relaxation modulus measurements disturbed by independent additive noises selected according to uniform distribution on the interval $\left[-10,\;10\right]\;\mathrm{Pa}$ for the uni-mode Gauss spectrum $H\left(\tau \right)$ (32).

**Figure 6:**
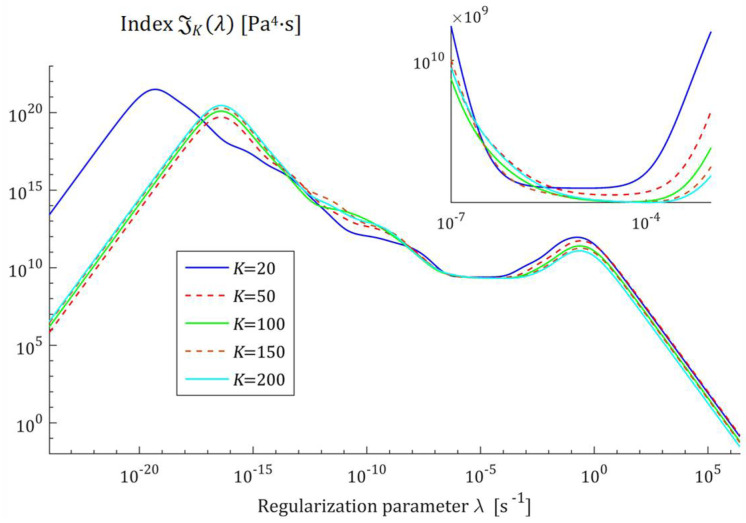
Square Multiplicative Regularization Index (SMRI) $J_{K}\left(\lambda \right)$ (53) as the function of the regularization parameter λ for K relaxation modulus measurements disturbed by independent additive noises selected according to uniform distribution on the interval $\left[-10,\;10\right]\;\mathrm{Pa}$ for the uni-mode Gauss spectrum $H\left(\tau \right)$ (32).

The courses of the indices $G_{K}\left(\lambda \right)$ (52) and $J_{K}\left(\lambda \right)$ (53) for the exemplary uni-mode Gauss like spectrum $H\left(\tau \right)$ (32) and experiment data collected in the experiment described above are presented in Figure 5 and Figure 6, respectively, for K=20, 50, 100, 150, 200. The courses of the indices $G_{K}\left(\lambda \right)$ (52) and $J_{K}\left(\lambda \right)$ (53) for double-mode Gauss-like $H\left(\tau \right)$ (36) and KWW (39) spectra are presented in Figures S12–S15 in Supplementary Materials.

Since by (54) and (55):

$$G_{K}\left(\lambda =0\right)=0,\;J_{K}\left(\lambda =0\right)=0$$

and, simultaneously:

$$\underset{\lambda \to \infty }{\lim}G_{K}\left(\lambda \right)=0^{+},\;\underset{\lambda \to \infty }{\lim}J_{K}\left(\lambda \right)=0^{+},$$

the nonnegative definite indices $J_{K}\left(\lambda \right)$ and $G_{K}\left(\lambda \right)$ have at least one extreme. The numerical studies shown that for 5≤K≤500, there are three local extrema, two maxima and one minimum; Figure 5 and Figure 6, and Figures S12–S15 in Supplementary Materials show the corresponding illustrations.

Therefore, the following tasks of the optimal regularization parameter selection can be introduced for the Multiplicative Regularization Index:

(56) $$\min\left\{\lambda :\;\lambda =\arg\;\underset{\lambda >0}{\mathrm{local}\;\min}G_{K}\left(\lambda \right)\right\},$$

and for the Square Multiplicative Regularization Index:

(57) $$\min\left\{\lambda :\;\lambda =\arg\;\underset{\lambda >0}{\mathrm{local}\;\min}J_{K}\left(\lambda \right)\right\}.$$

The notation applied in (56) and (57) means that the minimal regularization parameters being local minima (local min) of the indices $J_{K}\left(\lambda \right)$ and $G_{K}\left(\lambda \right)$ are selected. However, the numerical studies show that in all the tested examples, indices $J_{K}\left(\lambda \right)$ and $G_{K}\left(\lambda \right)$ have only one local minimum, therefore the next tasks:

(58) $$\underset{\lambda >0}{\mathrm{local}\;\min}G_{K}\left(\lambda \right)=G_{K}\left(\lambda^{*}\right)$$

and:

(59) $$\underset{\lambda >0}{\mathrm{local}\;\min}J_{K}\left(\lambda \right)=J_{K}\left(\lambda^{**}\right)$$

of the optimal MRI and SMRI rules are uniquely solved.

Both new multiplicative indices introduce a tradeoff between a good fit of the relaxation modulus experiment data and a good smoothness of the relaxation spectra models. The indices $J_{K}\left(\lambda \right)$ and $G_{K}\left(\lambda \right)$ are continuous nonnegative differentiable functions of nonnegative λ. The first and second order optimality conditions are derived in the next subsections.

### 3.3. Optimality Conditions of the MRI Rule

By (44) and (47), for index $G_{K}\left(\lambda \right)$ (54) after algebraic manipulations, we have:

(60) $$\frac{dG_{K}\left(\lambda \right)}{d\lambda }=\frac{\alpha^{3}\lambda \left[\sum_{k=1}^{K}\frac{y_{k}^{2}\sigma_{k}}{{\left(\sigma_{k}+\alpha \lambda \right)}^{3}}\right]}{K\sqrt{\sum_{k=1}^{K}\;\frac{\alpha \sigma_{k}y_{k}^{2}}{{\left(\sigma_{k}+\alpha \lambda \right)}^{2}}}}G\left(\lambda \right),$$

with the function $G\left(\lambda \right)$ defined by:

(61) $$G\left(\lambda \right)=\sum_{k=1}^{K}\;\frac{\left(2\sigma_{k}-\alpha \lambda \right)y_{k}^{2}}{{\left(\sigma_{k}+\alpha \lambda \right)}^{2}}.$$

Therefore, for the $\alpha \lambda \leq 2\sigma_{K}$ function, $G_{K}\left(\lambda \right)$ monotonically increases starting with the boundary value $G_{K}\left(0\right)=0$, while for $\alpha \lambda \geq 2\sigma_{1}$, it monotonically decreases to $G_{K}\left(\lambda \to \infty \right)=0$; compare Figure 5 as well as Figures S12 and S13 in Supplementary Materials. By (60), the stationary condition of nonzero local minima and maxima of $G_{K}\left(\lambda \right)$ is as follows:

(62) $$G\left(\lambda^{*}\right)=0,$$

which, in view of the boundary conditions:

$$G\left(\lambda =0\right)=\sum_{k=1}^{K}\;\frac{\sigma_{k}y_{k}^{2}}{{\left(\sigma_{k}+\alpha \lambda \right)}^{2}}>0,\;\underset{\lambda \to \infty }{\lim}G\left(\lambda \right)=0^{-},$$

has at least one solution. The unified solution $\alpha \lambda^{*}$ of (62) depends on the singular values $\left\{\sigma_{k}\right\}$ and the vector $Y_{K}$ (27), which, in turn, depends on the matrix $U_{K}$ and the relaxation modulus measurements. The upper and lower bounds of $\alpha \lambda^{*}$ follow from monotonicity of the sequence $\left\{\sigma_{k}\right\}$ and are as follows:

(63) $$2\sigma_{K}<\alpha \lambda^{*}<2\sigma_{1}.$$

Based on (60) and (61), in Appendix A.1, the following formula describing the second derivative of $G_{K}\left(\lambda \right)$ is derived:

(64) $$\frac{d^{2}G_{K}\left(\lambda \right)}{d\lambda^{2}}=\frac{\alpha^{3}\left[\sum_{k=1}^{K}\frac{y_{k}^{2}\sigma_{k}}{{\left(\sigma_{k}+\alpha \lambda \right)}^{3}}\right]G\left(\lambda \right)}{K\sqrt{\sum_{k=1}^{K}\;\frac{\alpha \sigma_{k}y_{k}^{2}}{{\left(\sigma_{k}+\alpha \lambda \right)}^{2}}}}+\frac{\alpha^{4}\lambda {\left[\sum_{k=1}^{K}\frac{y_{k}^{2}\sigma_{k}}{{\left(\sigma_{k}+\alpha \lambda \right)}^{3}}\right]}^{2}G\left(\lambda \right)}{K\sqrt{\sum_{k=1}^{K}\;\frac{\alpha \sigma_{k}y_{k}^{2}}{{\left(\sigma_{k}+\alpha \lambda \right)}^{2}}}\left[\sum_{k=1}^{K}\;\frac{\sigma_{k}y_{k}^{2}}{{\left(\sigma_{k}+\alpha \lambda \right)}^{2}}\right]}+\;\frac{\alpha^{4}\lambda \left\{-3\alpha \left[\sum_{k=1}^{K}\frac{y_{k}^{2}\sigma_{k}}{{\left(\sigma_{k}+\alpha \lambda \right)}^{4}}\right]G\left(\lambda \right)+\left[\sum_{k=1}^{K}\frac{y_{k}^{2}\sigma_{k}}{{\left(\sigma_{k}+\alpha \lambda \right)}^{3}}\right]\left[\sum_{k=1}^{K}\;\frac{y_{k}^{2}}{{\left(\sigma_{k}+\alpha \lambda \right)}^{2}}-\sum_{k=1}^{K}\;\frac{6\sigma_{k}y_{k}^{2}}{{\left(\sigma_{k}+\alpha \lambda \right)}^{3}}\right]\right\}}{K\sqrt{\sum_{k=1}^{K}\;\frac{\alpha \sigma_{k}y_{k}^{2}}{{\left(\sigma_{k}+\alpha \lambda \right)}^{2}}}}.$$

Therefore, for the extreme $\lambda^{*}$, according to (62), we have:

$$\frac{d^{2}G_{K}\left(\lambda =\lambda^{*}\right)}{d\lambda^{2}}=\frac{\alpha^{4}\lambda^{*}\left[\sum_{k=1}^{K}\frac{y_{k}^{2}\sigma_{k}}{{\left(\sigma_{k}+\alpha \lambda^{*}\right)}^{3}}\right]\left[\sum_{k=1}^{K}\;\frac{y_{k}^{2}}{{\left(\sigma_{k}+\alpha \lambda^{*}\right)}^{2}}-\sum_{k=1}^{K}\;\frac{6\sigma_{k}y_{k}^{2}}{{\left(\sigma_{k}+\alpha \lambda^{*}\right)}^{3}}\right]}{K\sqrt{\sum_{k=1}^{K}\;\frac{\alpha \sigma_{k}y_{k}^{2}}{{\left(\sigma_{k}+\alpha \lambda^{*}\right)}^{2}}}},$$

which means that $\frac{d^{2}G_{K}\left(\lambda =\lambda^{*}\right)}{d\lambda^{2}}>0$ whenever the following inequality holds:

$$\sum_{k=1}^{K}\;\frac{y_{k}^{2}}{{\left(\sigma_{k}+\alpha \lambda^{*}\right)}^{2}}>\sum_{k=1}^{K}\;\frac{6\sigma_{k}y_{k}^{2}}{{\left(\sigma_{k}+\alpha \lambda^{*}\right)}^{3}},$$

which, combined with (62), results in the optimality conditions.

**Proposition 1.**  *Let the time-scale factor* α>0*. Regularization parameter* $\lambda^{*}$* is the solution to the regularization parameter selection MRI task (58) if and only if *$G\left(\lambda^{*}\right)=0$* and:*

(65) $$\sum_{k=1}^{K}\;\frac{\left(5\sigma_{k}-\alpha \lambda^{*}\right)y_{k}^{2}}{{\left(\sigma_{k}+\alpha \lambda^{*}\right)}^{3}}<0.$$

Rewriting function $G\left(\lambda \right)$ (61) as:

$$G\left(\lambda \right)=2\sum_{k=1}^{K}\frac{\sigma_{k}y_{k}^{2}}{{\left(\sigma_{k}+\alpha \lambda \right)}^{2}}-\alpha \lambda \sum_{k=1}^{K}\frac{y_{k}^{2}}{{\left(\sigma_{k}+\alpha \lambda \right)}^{2}}=2Y_{K}^{T}\;\Sigma_{K}^{1/2}\;\Omega_{K}^{2}\left(\lambda \right)\Sigma_{K}^{1/2}\;Y_{K}-\;\alpha \lambda \;Y_{K}^{T}\;\Omega_{K}^{2}\left(\lambda \right)Y_{K},$$

where diagonal K×K matrix function $\Omega_{K}\left(\lambda \right)$ is defined by (26), vector $Y_{K}$ by (27), the square root $\Sigma_{K}^{1/2}$ of the matrix $\Sigma_{K}$ (24) is given by:

(66) $$\Sigma_{K}^{1/2}=diag\left(\sqrt{\sigma_{1}},\ldots ,\sqrt{\sigma_{K}}\right),$$

and expressing the left-hand side of (65) as:

$$\sum_{k=1}^{K}\frac{5\sigma_{k}y_{k}^{2}}{{\left(\sigma_{k}+\alpha \lambda^{*}\right)}^{3}}-\alpha \lambda^{*}\sum_{k=1}^{K}\frac{y_{k}^{2}}{{\left(\sigma_{k}+\alpha \lambda^{*}\right)}^{3}}=5Y_{K}^{T}\;\Sigma_{K}^{1/2}\;\Omega_{K}^{3}\left(\lambda^{*}\right)\Sigma_{K}^{1/2}\;Y_{K}-\;\alpha \lambda^{*}\;Y_{K}^{T}\;\Omega_{K}^{3}\left(\lambda^{*}\right)Y_{K}.$$

we can reformulate the above result in the vector-matrix terms as follows.

**Proposition 2.**  *Let the time-scale factor* α>0*. Regularization parameter* $\lambda^{*}$* is the solution to the regularization parameter optimal choice MRI task (58) if and only if the following equation:*

$$2Y_{K}^{T}\;\Sigma_{K}^{1/2}\;\Omega_{K}^{2}\left(\lambda^{*}\right)\Sigma_{K}^{1/2}\;Y_{K}=\alpha \lambda^{*}\;Y_{K}^{T}\;\Omega_{K}^{2}\left(\lambda^{*}\right)Y_{K},$$

*and inequality:*

$$5Y_{K}^{T}\;\Sigma_{K}^{1/2}\Omega_{K}^{3}\left(\lambda^{*}\right)\Sigma_{K}^{1/2}\;Y_{K}<\alpha \lambda^{*}\;Y_{K}^{T}\;\Omega_{K}^{3}\left(\lambda^{*}\right)Y_{K}$$

*hold.*

### 3.4. Optimality Conditions of the SMRI Rule

By (44) and (47), after algebraic manipulations, we have:

(67) $$\frac{dJ_{K}\left(\lambda \right)}{d\lambda }=\frac{2\alpha^{3}}{K}\lambda \left[\sum_{k=1}^{K}\;\frac{\sigma_{k}y_{k}^{2}}{{\left(\sigma_{k}+\alpha \lambda \right)}^{3}}\right]F\left(\lambda \right),$$

where function $F\left(\lambda \right)$ is defined by:

(68) $$F\left(\lambda \right)=\sum_{k=1}^{K}\;\frac{\left(\sigma_{k}-\alpha \lambda \right)y_{k}^{2}}{{\left(\sigma_{k}+\alpha \lambda \right)}^{2}}.$$

Therefore, for $\alpha \lambda \leq \sigma_{K}$, function $J_{K}\left(\lambda \right)$ monotonically increases from the boundary value from $J_{K}\left(0\right)=0$, while for $\alpha \lambda \geq \sigma_{1}$, it monotonically decreases to $J_{K}\left(\lambda \to \infty \right)=0$; see Figure 6 and Figures S14 and S15 in Supplementary Materials. By (67), the stationary condition of nonzero local minima and maxima of $J_{K}\left(\lambda \right)$ is as follows:

(69) $$F\left(\lambda^{**}\right)=0,$$

And, in view of the previous analysis of the index $J_{K}\left(\lambda \right)$, monotonicity has at least one solution. Similarly, as $\alpha \lambda^{*}$, the unified solution $\alpha \lambda^{**}$ of (69) depends only on the singular values $\left\{\sigma_{k}\right\}$ and on the vector $Y_{K}$ (27). The upper and lower bounds follow from the sequence $\left\{\sigma_{k}\right\}$ monotonicity and are as follows:

(70) $$\sigma_{K}<\alpha \lambda^{**}<\sigma_{1}.$$

In Appendix A.2, the following formula describing the second derivative of $J_{K}\left(\lambda \right)$ is derived:

(71) $$\frac{d^{2}J_{K}\left(\lambda \right)}{d\lambda^{2}}=\frac{2\alpha^{3}}{K}\left[\sum_{k=1}^{K}\;\frac{\sigma_{k}y_{k}^{2}}{{\left(\sigma_{k}+\alpha \lambda \right)}^{3}}\right]F\left(\lambda \right)-\;\frac{2\alpha^{4}}{K}\lambda \left\{3\sum_{k=1}^{K}\;\frac{\sigma_{k}y_{k}^{2}}{{\left(\sigma_{k}+\alpha \lambda \right)}^{4}}F\left(\lambda \right)+\left[\sum_{k=1}^{K}\;\frac{\sigma_{k}y_{k}^{2}}{{\left(\sigma_{k}+\alpha \lambda \right)}^{3}}\right]\left[\sum_{k=1}^{K}\;\frac{\left(3\sigma_{k}-\alpha \lambda \right)y_{k}^{2}}{{\left(\sigma_{k}+\alpha \lambda \right)}^{3}}\right]\right\}.$$

Therefore, for any stationary point $\lambda^{**}$ of $J_{K}\left(\lambda \right)$, bearing in mind Equation (69), we have:

$$\frac{d^{2}J_{K}\left(\lambda =\lambda^{**}\right)}{d\lambda^{2}}=-\frac{2\alpha^{4}}{K}\lambda^{**}\left\{\left[\sum_{k=1}^{K}\;\frac{\sigma_{k}y_{k}^{2}}{{\left(\sigma_{k}+\alpha \lambda^{**}\right)}^{3}}\right]\left[\sum_{k=1}^{K}\;\frac{\left(3\sigma_{k}-\alpha \lambda^{**}\right)y_{k}^{2}}{{\left(\sigma_{k}+\alpha \lambda^{**}\right)}^{3}}\right]\right\}$$

and the following optimality conditions results.

**Proposition 3.**  *Let the time-scale factor* α>0*. Regularization parameter* $\lambda^{**}$* is the solution to the regularization parameter selection SMRI task (59) if and only if* $F\left(\lambda^{**}\right)=0$* and:*

$$\sum_{k=1}^{K}\;\frac{\left(3\sigma_{k}-\alpha \lambda^{**}\right)y_{k}^{2}}{{\left(\sigma_{k}+\alpha \lambda^{**}\right)}^{3}}<0.$$

The equivalent vector-matrix form of the optimality conditions is as follows.

**Proposition 4.**  *Let the time-scale factor* α>0. *Regularization parameter* $\lambda^{**}$* is the solution to the regularization parameter selection SMRI task (59) if and only if the following equation:*

$$Y_{K}^{T}\;\Sigma_{K}^{1/2}\;\Omega_{K}^{2}\left(\lambda^{**}\right)\Sigma_{K}^{1/2}\;Y_{K}=\alpha \lambda^{**}\;Y_{K}^{T}\;\Omega_{K}^{2}\left(\lambda^{**}\right)Y_{K},$$

* and inequality:*

$$3Y_{K}^{T}\;\Sigma_{K}^{1/2}\;\Omega_{K}^{3}\left(\lambda^{**}\right)\Sigma_{K}^{1/2}\;Y_{K}<\alpha \lambda^{**}\;Y_{K}^{T}\;\Omega_{K}^{3}\left(\lambda^{**}\right)Y_{K}$$

* hold, with the diagonal matrices* $\Omega_{K}\left(\lambda \right)$* (26) and* $\Sigma_{K}^{1/2}$*(66) and the vector* $Y_{K}$* (27).*

### 3.5. Relation Between the MRI and SMRI Rules

Comparison of (68) and (61) immediately yields identity:

$$G\left(\lambda \right)=\sum_{k=1}^{K}\;\frac{2\sigma_{k}y_{k}^{2}}{{\left(\sigma_{k}+\alpha \lambda \right)}^{2}}+F\left(\lambda \right)$$

valid for any λ>0. As such, in view of the stationary point condition (69) for the minimum $\lambda^{**}$ of the multiplicative index $J_{K}\left(\lambda \right)$ (53), we have $G\left(\lambda^{**}\right)>0$, which, owing to (60), means that index $G_{K}\left(\lambda \right)$ grows for $\lambda =\lambda^{**}$. Simultaneously, for $\lambda^{*}$ minimizing $G_{K}\left(\lambda \right)$, condition $G\left(\lambda^{*}\right)=0$ means that $F\left(\lambda^{*}\right)<0$, i.e., according to (67) index $J_{K}\left(\lambda \right)$ decreases for $\lambda =\lambda^{*}$. Therefore, having in mind the courses of the indices $J_{K}\left(\lambda \right)$ and $G_{K}\left(\lambda \right)$ (cf., Figure 5 and Figure 6 and Figures S12–S15 in Supplementary Materials), the following relation between the two minima results:

$$\lambda^{*}<\lambda^{**},$$

which means a stronger smoothing of the spectrum whenever index $J_{K}\left(\lambda \right)$ is applied.

### 3.6. Identification Scheme

In summary, for the calculation of the optimal relaxation spectra models, the following steps are needed:

1. For the investigated polymeric material, conduct the preliminary stress relaxation experiment [2,42] and register the measurements $\bar{G}\left(t_{i}\right)$, i=1,…,N, for pre-selected sampling times $t_{i}$, e.g., equidistant in the time interval $\left[0,T\right]$, T<∞.
2. Select the time-scaling factor α and the number K of model summands by comparison of a few functions $\left\{\phi_{k}\left(t\right)\right\}$ defined by (11), with the experiment results $\left\{\bar{G}\left(t_{i}\right)\right\}$ for different values of α.
3. Conduct the stress relaxation experiment and register the modulus measurements $\bar{G}\left(t_{k}\right)$ at time instants $t_{k}=\alpha \cdot k$, k=1,…,K.
4. Determine the matrix $\Phi_{K}$ (19), and next determine its SVD (23) decomposition.
5. Select the optimal regularization parameter $\lambda^{*}$ as the solution to the MRI optimization task (58) or parameter $\lambda^{**}$ by solving the SMRI minimization task (59).
6. For selected parameter $\lambda =\lambda^{*}$ or $\lambda =\lambda^{**}$, determine the regularized optimal model parameter ${\bar{g}}_{K}\left(\lambda \right)$ using Formula (25).
7. Determine the modified relaxation frequency spectrum ${\bar{H}}_{K}^{M}\left(v\right)$ applying Formula (28).
8. Compute, according to Formulas (30) and (29), the relaxation time ${\bar{H}}_{K}\left(\tau \right)$ and frequency ${\bar{H}}_{K}\left(v\right)$ spectra models as the linear combinations of the basis functions $h_{k}\left(\tau \right)$ and $h_{k}\left(v\right)v$, respectively.

Note that Steps 1–4 and 6–8 are identical with those of the identification algorithm presented in [24]; only Step 5, concerning the optimal regularization parameter selection, is introduced here. In [24], the simple Spectral Condition Number Rule (SCNR) is applied, according to which the parameters λ were selected, such that:

(72) $$\kappa \left(\Phi_{K}+\alpha \lambda I_{K,K}\right)=\frac{\sigma_{1}+\alpha \lambda }{\sigma_{K}+\alpha \lambda }\leq 10^{5},$$

where the spectral conditional number $\kappa \left(\Phi_{K}+\alpha \lambda I_{K,K}\right)$, for definition see Equation (3) in [24] or Equation (2.6.3) in [45], measures the sensitivity of the answer to small perturbations of the measurement data. The rule (72) means that the condition number does not exceed the numerically acceptable value 10^5^ [25].

Since the matrix $\Phi_{K}$ (19), consequently, the singular values $\sigma_{k}$ of $\Phi_{K}$, do not depend on the time-scale factor α, for fixed K, the SVD, being accessible as the numerical procedures of computational complexity $O\left(NK^{2}\right)$ [45] in most computational packets, must be computed only once, even if the α-dependent sampling instants $t_{k}=\alpha k$ are changed in the experiment.

Owing to (63) and (70), only restricted intervals of the regularization parameter values can be considered in the numerical solution of the optimization tasks (58) and (59).

The proposed identification algorithm based on the MRI or SMRI rules is, in fact, a two-level identification scheme in which the upper level task consists of solving the problem of the optimal regularization parameter selection, while the regularized linear-quadratic problem of the model parameter optimal determination is solved in the lower level. However, owing to the simple analytical formula describing the lower level task solution, only the upper level nonlinear optimization tasks must be numerically solved to determine the best regularization parameters for which the optimal model parameters are computed only once.

### 3.7. Numerical Studies

This section presents the application of the MRI and SMRI rules for selecting the optimal regularization parameters for the Gauss-like and KWW spectra introduced in Section 2.6. Simulation verification of the relaxation spectra identification method, based on the pre-assumed real relaxation spectra description, is an initial and necessary step of the method validation because the effectiveness of the identification method can only be verified when the identified spectrum is exactly known. This is an obvious requirement in the case of ill-posed inverse problems, and such is the task of the relaxation spectrum determination.

For both regularization parameter selection rules, the optimal regularization parameters $\lambda^{*}$ and $\lambda^{**}$, solving the optimization problems (58) and (59), and the best spectra models ${\bar{H}}_{K}^{M}\left(v\right)$ (28), ${\bar{H}}_{K}\left(v\right)$ (29) and ${\bar{H}}_{K}\left(\tau \right)$ (30), were determined for a few numbers of measurements, applying the algorithm described above.

The model smoothness is estimated by their integral square norm, which for ${\bar{H}}_{K}^{M}\left(v\right)$ and ${\bar{H}}_{K}\left(\tau \right)$, according to (42), is given by:

(73) $$\|{\bar{H}}_{K}^{M}\left(v\right){\|}_{2}=\|{\bar{H}}_{K}\left(\tau \right){\|}_{2}=\frac{1}{\sqrt{\alpha }}\sqrt{{\bar{g}}_{K}^{T}\left(\lambda \right)\;\Phi_{K}{\bar{g}}_{K}\left(\lambda \right)}.$$

In view of Equation (62) in [24], we have:

(74) $$\|{\bar{H}}_{K}\left(v\right){\|}_{2}=\frac{\sqrt{2}}{\alpha \sqrt{\alpha }}\sqrt{{\bar{g}}_{K}^{T}\left(\lambda \right)\;\Theta_{K}{\bar{g}}_{K}\left(\lambda \right)},$$

with the K×K symmetric matrix $\Theta_{K}$ composed of the elements $\theta_{ij}=\frac{1}{{\left(i+j\right)}^{3}}$. Index $Q_{K}\left({\bar{g}}_{K}\left(\lambda \right)\right)$ (45) estimates the errors of the relaxation modulus models. The errors of the relaxation spectra optimal models are measured directly by the integral $J\left(g_{K}\right)$ (15), which by (16) and (19), for the optimal models with parameter ${\bar{g}}_{K}\left(\lambda \right)$ (22), is given by:

(75) $$J\left({\bar{g}}_{K}\left(\lambda \right)\right)=\int_{0}^{\infty }H^{M}{\left(v\right)}^{2}dv-2G_{K}^{T}\;{\bar{g}}_{K}\left(\lambda \right)+\frac{1}{\alpha }{\bar{g}}_{K}^{T}\left(\lambda \right)\Phi_{K}{\bar{g}}_{K}\left(\lambda \right),$$

where $G_{K}={\left[\begin{array}{ccc} G\left(t_{1}\right) & \cdots & G\left(t_{K}\right) \end{array}\right]}^{T}$ is the vector of noise-free values of relaxation modulus. All the indices are determined for parameters ${\bar{g}}_{K}\left(\lambda^{*}\right)$ and ${\bar{g}}_{K}\left(\lambda^{**}\right)$ resulting from the application of MRI and SMRI rules, whose smoothness is rated by the square vector norms $\|{\bar{g}}_{K}\left(\lambda^{*}\right){\|}_{2}$ and $\|{\bar{g}}_{K}\left(\lambda^{**}\right){\|}_{2}$.

The ‘real’ polymer and the optimal models were simulated in Matlab R2023b, The Mathworks, Inc., Natick, MA, USA. For the singular value decomposition procedure, *svd* was applied.

#### 3.7.1. Direct Identification of the Uni-Mode Gauss-like Spectrum

**Figure 7:**
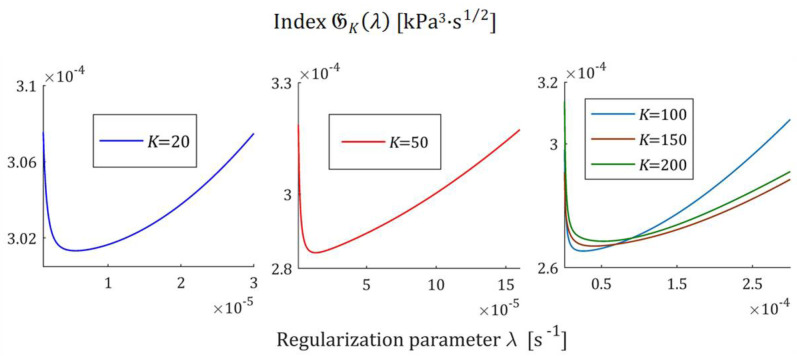
Multiplicative Regularization Index $G_{K}\left(\lambda \right)$ (52) as the function of the regularization parameter λ for K relaxation modulus measurements disturbed by independent additive noises selected according to uniform distribution on the interval $\left[-10,\;10\right]\;\mathrm{Pa}$ for the uni-mode Gauss spectrum $H\left(\tau \right)$ (32) in the neighborhood of its local minimum $\lambda^{*}$.

**Figure 8:**
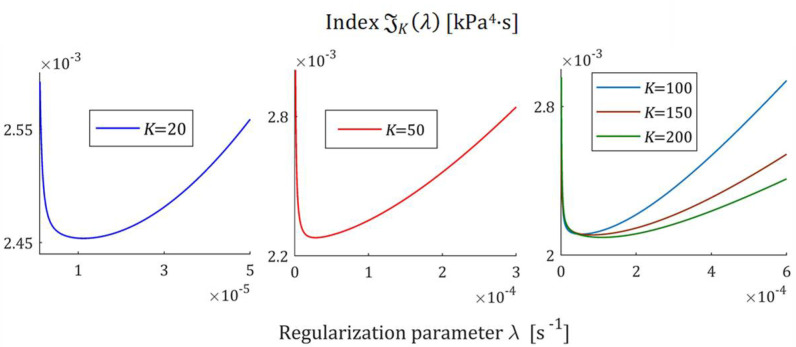
The SMRI $J_{K}\left(\lambda \right)$ (53) as the function of the regularization parameter λ for K relaxation modulus measurements disturbed by independent additive noises selected according to uniform distribution on the interval $\left[-10,\;10\right]\;\mathrm{Pa}$ for the uni-mode Gauss spectrum $H\left(\tau \right)$ (32) in the neighborhood of its local minimum $\lambda^{**}$.

For the spectra $H\left(\tau \right)$ (32) and $H^{M}\left(v\right)$ (35), the courses of MRI $G_{K}\left(\lambda \right)$ (52) and SMRI $J_{K}\left(\lambda \right)$ (53) indices in the neighborhoods of their local minima, selected based on the inspection of Figure 5 and Figure 6, are presented in Figure 7 and Figure 8, respectively. Here, linear scales are used for both axes. The optimal regularization parameters $\lambda^{*}$ and $\lambda^{**}$ solving the optimization problems (58) and (59) are given in Table 1 and Table 2, respectively, where the optimal values $G_{K}\left(\lambda^{*}\right)$ and $J_{K}\left(\lambda^{**}\right)$ of MRI and SMRI are also enclosed. The norms $\|{\bar{g}}_{K}\left(\lambda^{*}\right){\|}_{2}$ and $\|{\bar{g}}_{K}\left(\lambda^{**}\right){\|}_{2}$, and the norms $\|{\bar{H}}_{K}^{M}\left(v\right){\|}_{2}=\|{\bar{H}}_{K}\left(\tau \right){\|}_{2}$ (73) and $\|{\bar{H}}_{K}\left(v\right){\|}_{2}$ (74), the square indices $J\left({\bar{g}}_{K}\left(\lambda \right)\right)$ (75) and $Q_{K}\left({\bar{g}}_{K}\left(\lambda \right)\right)$ (45) are also given in these tables.

**Figure 9:**
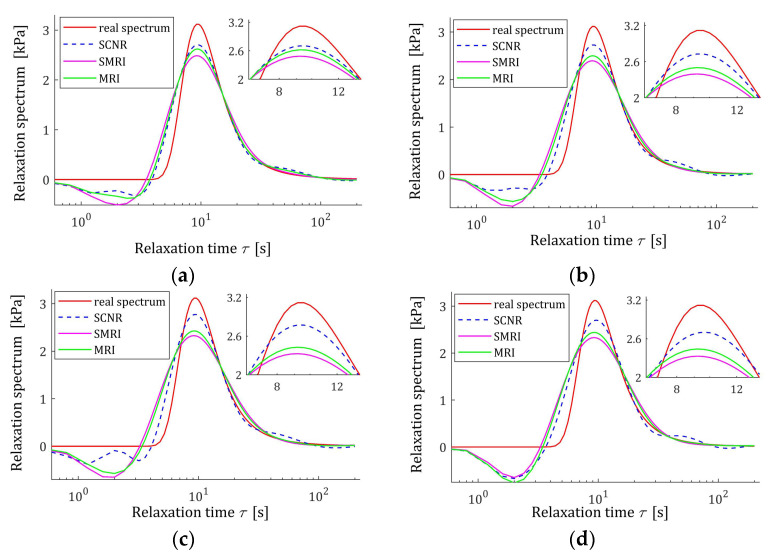
Uni-mode Gauss relaxation time spectrum $H\left(\tau \right)$ (32) (solid red line) and the corresponding models ${\bar{H}}_{K}\left(\tau \right)$ (30) for K relaxation modulus measurements disturbed by independent additive noises selected according uniform distribution on the interval $\left[-10,\;10\right]\;\mathrm{Pa}$ determined for regularization parameters computed using MRI (58) and SMRI (59) rules and Spectral Condition Number Rule (SCNR), Equation (72): (**a**) K=50; (**b**) K=100; (**c**) K=150; (**d**) K=200.

**Figure 10:**
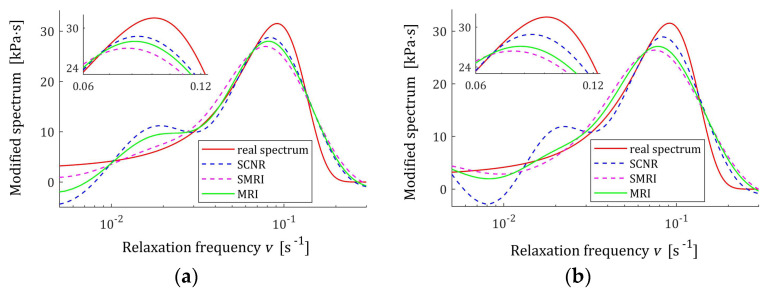

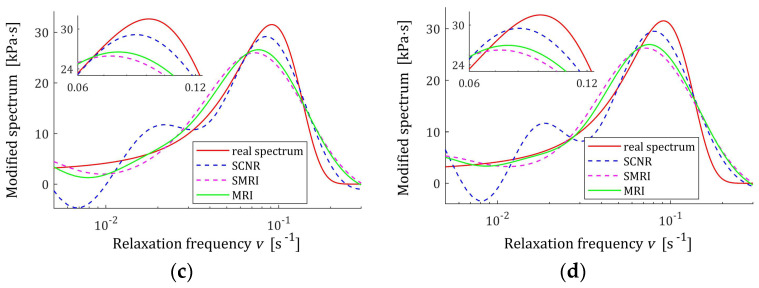
Modified Uni-mode Gauss spectrum $H^{M}\left(v\right)$ (35) (solid red line) and the corresponding models ${\bar{H}}_{K}^{M}\left(v\right)$ (28) for K relaxation modulus measurements disturbed by independent additive noises selected according uniform distribution on the interval $\left[-10,\;10\right]\;\mathrm{Pa}$ determined for regularization parameters computed using MRI (58), SMRI (59) and SCNR (72) rules: (**a**) K=50; (**b**) K=100; (**c**) K=150; (**d**) K=200.

**Figure 11:**
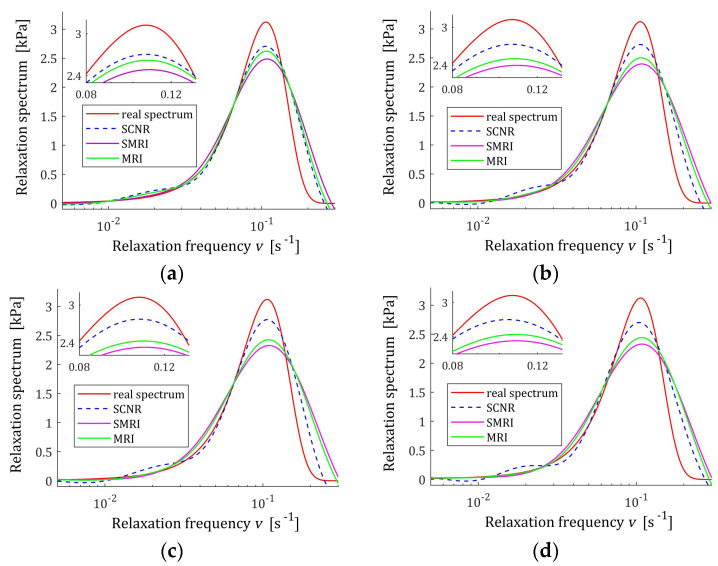
Uni-mode Gauss frequency spectrum $H\left(v\right)$ (34) (solid red line) and the corresponding models ${\bar{H}}_{K}\left(v\right)$ (29) for K relaxation modulus measurements disturbed by independent additive noises selected according uniform distribution on the interval $\left[-10,\;10\right]\;\mathrm{Pa}$ determined for regularization parameters computed using MRI (58), SMRI (59) and SCNR (72) rules: (**a**) K=50; (**b**) K=100; (**c**) K=150; (**d**) K=200.

The best models, determined using MRI and SMRI rules, are depicted in Figure 9, Figure 10 and Figure 11, together with the real spectra (32), (34), and (35) marked by red lines. Small subfigures show fitting near the maximum of the real spectrum. The logarithmic time and frequency scales are used. In these figures, the spectra determined based on SCNR (72) are also given; the respective regularization parameters λ were given in Table 3 in [24].

An inspection of the above figures shows that for the number of K≥5 measurements for the tested methods, satisfactory approximation of the relaxation spectra was obtained while maintaining the consistency of the maxima of real spectra and their models. However, for the relaxation time spectrum, the model’s smoothness obtained by applying the MRI and SMRI rules is comparable to that obtained by the approximate SCNR method, while for the modified relaxation frequency spectrum, starting from K=100 measurements, the optimal MRI and SMRI methods provide significantly better smoothing of the spectrum than SCNR method. The relaxation frequency spectrum models are similar for the three methods. For each of the relaxation spectra, the best approximation of the real spectrum maximum is provided by the SCNR method. Larger optimal regularization parameters $\lambda^{*}$ and $\lambda^{**}$ result in a worse approximation of the maximum of real spectrum, although the maximum locations are similar, especially for the relaxation time and frequency spectra. In summary, for the case of a Gaussian spectrum with a middle relaxation times, the MRI rule can be recommended, which guarantees a compromise between a good smoothing of all three spectra models and a good approximation of the maxima of the real spectra.

#### 3.7.2. Direct Identification of the Double-Mode Gauss-like Spectrum

**Figure 12:**
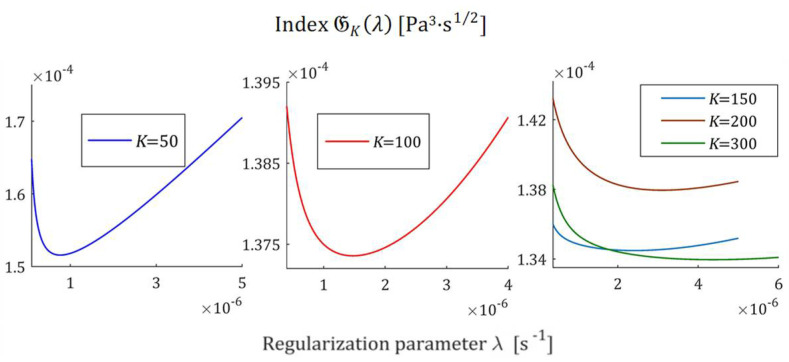
Multiplicative Regularization Index $G_{K}\left(\lambda \right)$ (52) as the function of the regularization parameter λ for K relaxation modulus measurements disturbed by independent additive noises selected according to uniform distribution on the interval $\left[-0.005,\;0.005\right]\;\mathrm{Pa}$ for the double-mode Gauss spectrum $H\left(\tau \right)$ (36) in the neighborhood of its local minimum $\lambda^{*}$.

**Figure 13:**
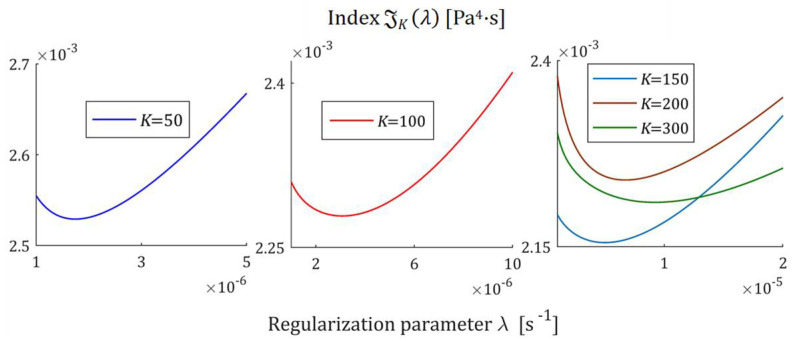
The SMRI $J_{K}\left(\lambda \right)$ (53) as the function of the regularization parameter λ for K relaxation modulus measurements disturbed by independent additive noises selected according to uniform distribution on the interval $\left[-0.005,\;0.005\right]\;\mathrm{Pa}$ for the double-mode Gauss spectrum $H\left(\tau \right)$ (36) in the neighborhood of its local minimum $\lambda^{**}$.

For the spectra $H\left(\tau \right)$ (36) and $H^{M}\left(v\right)=H\left(v\right)/v$, with relaxation frequency spectrum $H\left(v\right)$ (37), the courses of MRI $G_{K}\left(\lambda \right)$ (52) and SMRI $J_{K}\left(\lambda \right)$ (53) indices in the neighborhoods of their local minima are presented in Figure 12 and Figure 13, respectively; linear scales are used for both axes. The optimal regularization parameters $\lambda^{*}$ and $\lambda^{**}$ are given in Table 3 and Table 4, respectively, where the optimal values $G_{K}\left(\lambda^{*}\right)$ and $J_{K}\left(\lambda^{**}\right)$ are also enclosed together with the norms $\|{\bar{g}}_{K}\left(\lambda^{*}\right){\|}_{2}$ and $\|{\bar{g}}_{K}\left(\lambda^{**}\right){\|}_{2}$, norms $\|{\bar{H}}_{K}^{M}\left(v\right){\|}_{2}=\|{\bar{H}}_{K}\left(\tau \right){\|}_{2}$ (73) and $\|{\bar{H}}_{K}\left(v\right){\|}_{2}$ (74), and the square indices $J\left({\bar{g}}_{K}\left(\lambda \right)\right)$ (75) and $Q_{K}\left({\bar{g}}_{K}\left(\lambda \right)\right)$ (45).

**Figure 14:**
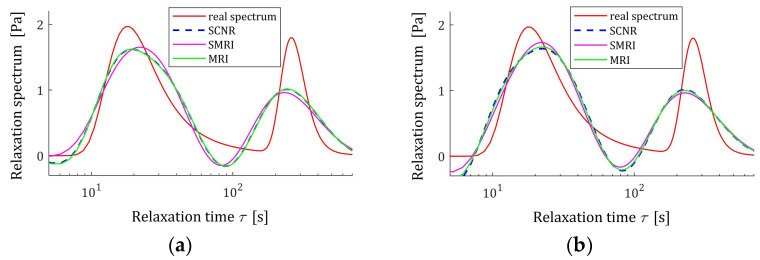

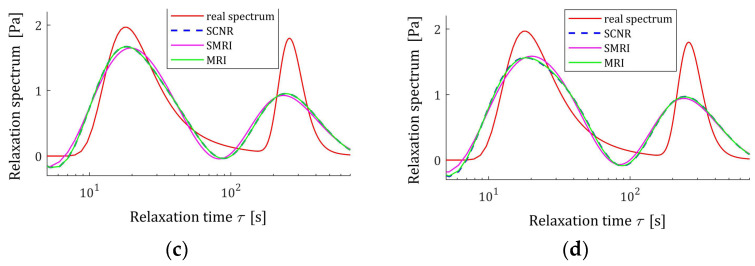
Double-mode Gauss relaxation time spectrum $H\left(\tau \right)$ (36) (solid red line) and the corresponding models ${\bar{H}}_{K}\left(\tau \right)$ (30) for K relaxation modulus measurements disturbed by independent additive noises selected according to uniform distribution on the interval $\left[-0.005,\;0.005\right]\;\mathrm{Pa}$ determined for regularization parameters computed using MRI (58), SMRI (59) and SCNR (72) rules: (**a**) K=50; (**b**) K=100; (**c**) K=200; (**d**) K=300.

**Figure 15:**
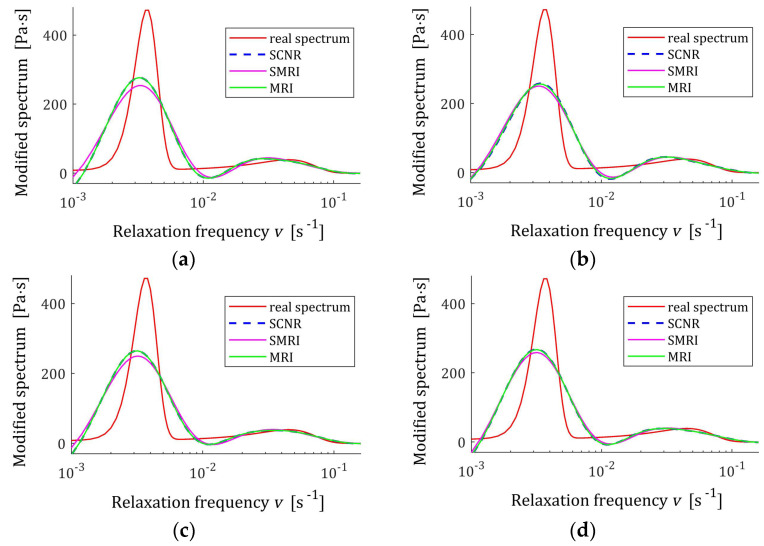
Modified double-mode Gauss spectrum $H^{M}\left(v\right)=H\left(v\right)/v$ (solid red line) defined by the spectrum $H\left(v\right)$ (37) and the corresponding models ${\bar{H}}_{K}^{M}\left(v\right)$ (28) for K measurements of the relaxation modulus corrupted by additive independent noises uniformly distributed on the interval $\left[-0.005,\;0.005\right]\;\mathrm{Pa}$ determined for regularization parameters computed using MRI (58), SMRI (59) and SCNR (72) rules: (**a**) K=50; (**b**) K=100; (**c**) K=200; (**d**) K=300.

**Figure 16:**
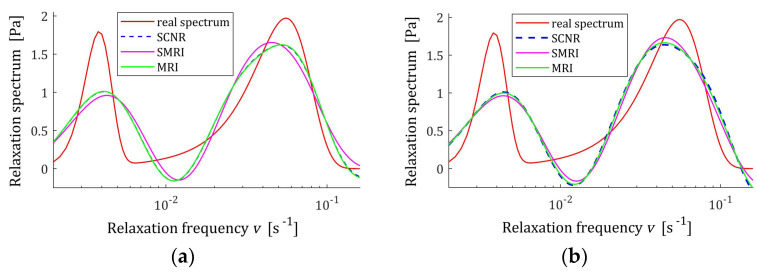

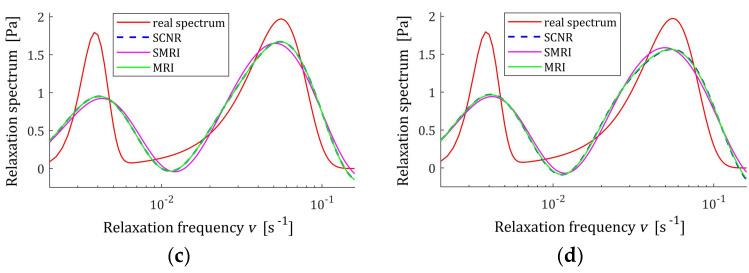
Double-mode Gauss relaxation frequency spectrum $H\left(v\right)$ (37) (solid red line) and the corresponding models ${\bar{H}}_{K}\left(v\right)$ (29) for K relaxation modulus measurements disturbed by independent additive noises selected according uniform distribution on the interval $\left[-0.005,\;0.005\right]\;\mathrm{Pa}$ determined for regularization parameters computed using MRI (58), SMRI (59) and SCNR (72) rules: (**a**) K=50; (**b**) K=100; (**c**) K=200; (**d**) K=300.

The best models determined using MRI and SMRI rules are depicted in Figure 14, Figure 15 and Figure 16; the real spectra $H\left(\tau \right)$ (36), $H\left(v\right)$ (37), and $H^{M}\left(v\right)=H\left(v\right)/v$ are marked by red lines. Small subfigures show fitting near the maximum of the real spectrum. The logarithmic horizontal axes are applied. In these figures, the spectra resulting from application of SCNR (72) are also given; the SCNR regularization parameters can be found in Table 4 in [24].

For double-mode long-relaxation times Gaussian spectra, the spectra models recovered by the three methods are almost similar. Therefore, the MRI rule can be recommended because, for most K, it provides the best approximation of the real spectra maxima.

#### 3.7.3. Direct Identification of the KWW Spectrum

**Figure 17:**
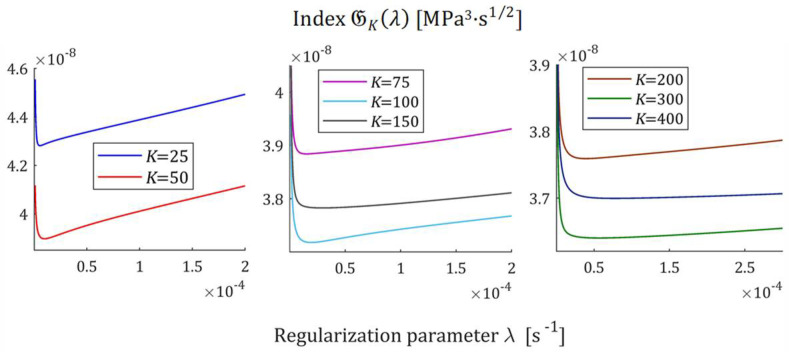
Multiplicative Regularization Index $G_{K}\left(\lambda \right)$ (52) as the function of the regularization parameter λ for K relaxation modulus measurements disturbed by independent additive noises selected according to uniform distribution on the interval $\left[-0.5,\;0.5\right]\;\mathrm{kPa}$ for the KWW relaxation spectrum $H\left(\tau \right)$ (39) in the neighborhood of its local minimum $\lambda^{*}$.

**Figure 18:**
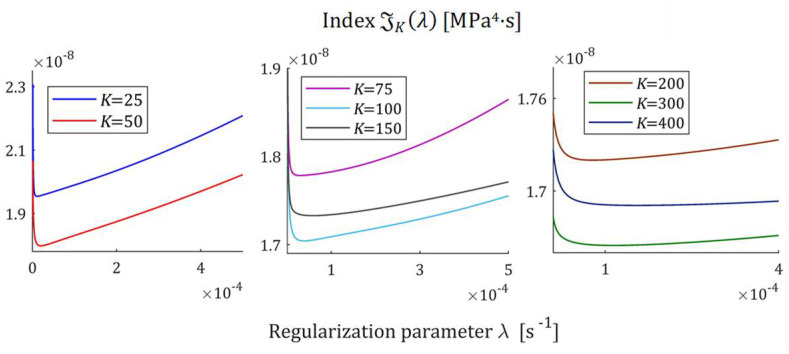
The SMRI $J_{K}\left(\lambda \right)$ (53) as the function of the regularization parameter λ for K relaxation modulus measurements disturbed by independent additive noises selected according to uniform distribution on the interval $\left[-0.5,\;0.5\right]\;\mathrm{kPa}$ the KWW relaxation spectrum $H\left(\tau \right)$ (39) in the neighborhood of its local minimum $\lambda^{**}$.

The indices MRI $G_{K}\left(\lambda \right)$ (52) and SMRI $J_{K}\left(\lambda \right)$ (53) for the spectra $H\left(\tau \right)$ (39) and $H^{M}\left(v\right)$ (41) are plotted in Figure 17 and Figure 18 in the near neighborhoods of their local minima. The optimal regularization parameters $\lambda^{*}$ and $\lambda^{**}$ defined by (58) and (59) are given in Table 5 and Table 6, respectively, where the optimal values $G_{K}\left(\lambda^{*}\right)$ and $J_{K}\left(\lambda^{**}\right)$ of MRI and SMRI are also enclosed together with the other models’ quality indices.

**Figure 19:**
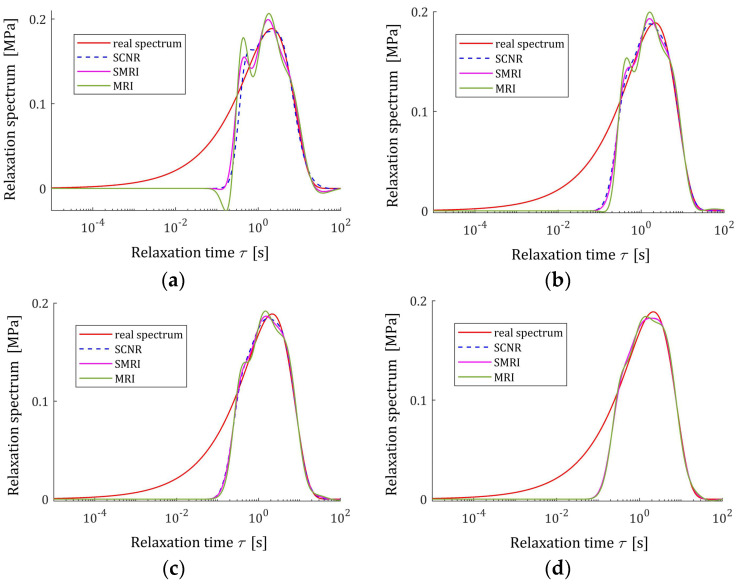
KWW spectrum $H\left(\tau \right)$ (39) (solid red line) and the corresponding models ${\bar{H}}_{K}\left(\tau \right)$ (30) for K relaxation modulus measurements disturbed by independent additive noises selected according to uniform distribution on the interval $\left[-0.5,\;0.5\right]\;\mathrm{kPa}$ determined for regularization parameters computed using MRI (58), SMRI (59) and SCNR (72) rules: (**a**) K=50; (**b**) K=100; (**c**) K=200; (**d**) K=400.

**Figure 20:**
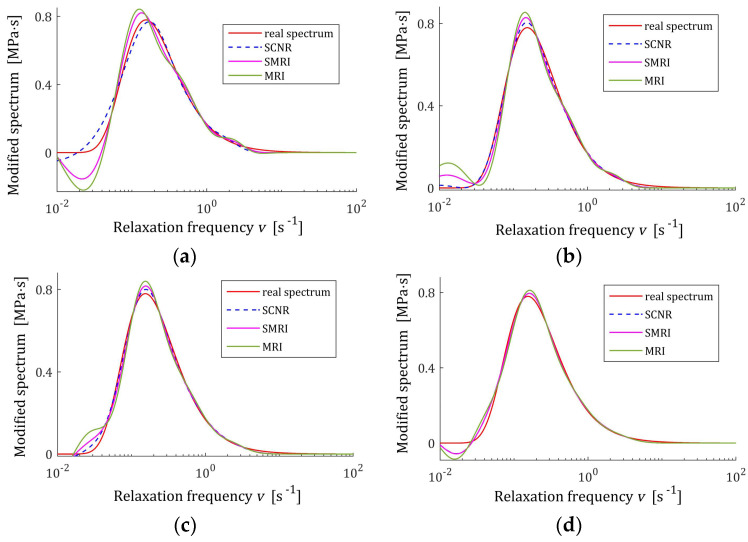
Modified KWW relaxation spectrum $H^{M}\left(v\right)$ (41) (solid red line) and the corresponding models ${\bar{H}}_{K}^{M}\left(v\right)$ (28) for K relaxation modulus measurements disturbed by independent additive noises selected according to uniform distribution on the interval $\left[-0.5,\;0.5\right]\;\mathrm{kPa}$ determined for regularization parameters computed using MRI (58), SMRI (59) and SCNR (72) rules: (**a**) K=50; (**b**) K=100; (**c**) K=200; (**d**) K=400.

**Figure 21:**
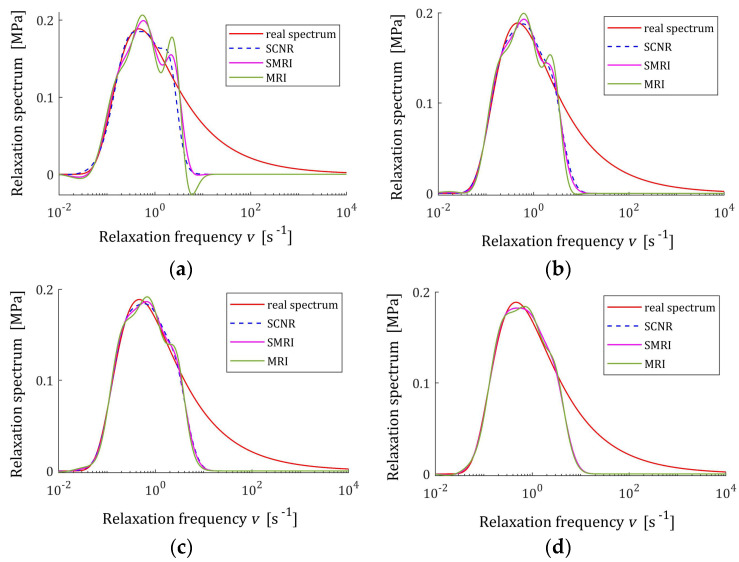
KWW relaxation frequency spectrum $H\left(v\right)$ (40) (solid red line) and the corresponding models ${\bar{H}}_{K}\left(v\right)$ (29) for K relaxation modulus measurements disturbed by independent additive noises selected according to uniform distribution on the interval $\left[-0.5,\;0.5\right]\;\mathrm{kPa}$ determined for regularization parameters computed using MRI (58), SMRI (59) and SCNR (72) rules: (**a**) K=50; (**b**) K=100; (**c**) K=200; (**d**) K=400.

The best models determined using MRI and SMRI rules are depicted in Figure 19, Figure 20 and Figure 21 for selected values of K with the real spectra $H\left(\tau \right)$ (36), $H\left(v\right)$ (37), and $H^{M}\left(v\right)=H\left(v\right)/v$ marked by red lines. The regularization parameters determined according to SCNR (72) can be found in Table 5 in [24]. Spectra determined for all K from the above tables can be found in Supplementary Materials, Section S.3 ‘Plots of the optimal models of the KWW relaxation spectra’.

The analysis of Figure 19, Figure 20 and Figure 21 and Figures S16–S18 in Supplementary Materials indicates that only reaching K≥200 measurements provide smoothing of the relaxation time and frequency spectra models in the vicinity of the maxima of the real characteristics, especially if the MRI rule is applied. For the modified relaxation frequency spectrum, the problem of model fluctuations in the near neighborhood of their maxima does not occur even for a small number of measurements. This spectrum is particularly well approximated by all models. Both new regularization parameter selection rules, MRI and SMRI, lead to the determination of models whose maxima, with locations almost identical to the locations of the maxima of the real characteristics, are slightly larger than them, especially when the MRI rule is used. Therefore, for the short relaxation times KWW spectrum, the SMRI rule can be suggested; however, the spectra models yielded by the MRI rule are also satisfactory.

## 4. Conclusions

The work continued research on a new concept of relaxation spectrum determination, according to which the identification index is related directly to the completely unknown and inaccessible by measurement relaxation spectrum—on the concept of spectrum direct identification introduced in the previous paper. Since, as shown, the classical known methods for selecting the regularization parameter cannot be effectively applied here, two new rules for the optimal selection of the regularization parameter for relaxation spectra direct identification were proposed. Both are based on the multiplicative regularization indices being defined as the products of integral square norms of the optimal spectra models and the mean square error of the respective relaxation modulus model.

The numerical studies demonstrated that applying the approach proposed, it is possible to determine the spectra models for a wide range of the relaxation times and frequencies of real polymers. Simulation studies have also shown that, in many cases, a not worse approximation of the real relaxation spectrum can be obtained by using a spectral conditional number rule SCNR. However, the simplicity and uniqueness of the MRI and SMRI rules, together with the ease of their application, are their advantages over the approximate SCNR rule, the ambiguity of which makes its practical application difficult. Assuming, for the basic regularized matrix, the upper bound of numerically acceptable value of the spectral conditional number may lead to excessive smoothing of the relaxation spectra models. In turn, the selection of the regularization parameter that guarantees a smaller value of the conditional number is not unique and may require many attempts.

In all tested examples, both new multiplicative regularization indices have only one local minimum. Nevertheless, the analytical studies concerning the existence, uniqueness, and location of the local minima can be continued.

Future studies will also concern the application of the regularization technique developed here for direct spectrum identification to data from a real stress relaxation experiment conducted for real materials. Comparison of the results with those obtained using other methods for identifying relaxation spectra from the relaxation test data is another direction of extensive research.

The new rules for the regularization parameter selection based on the multiplicative MRI and SMRI indices were derived specifically for the regularized task of direct relaxation spectrum identification. Analysis of the possibilities of transferring them to classic relaxation spectrum identification tasks (with the identification index related to the relaxation modulus when the stress relaxation experiment data are used) will be the subject of future research.

It is known that, sometimes, Tikhonov’s regularization technique fails. One alternative approach may then be to directly smooth the relaxation spectrum model. The search for an approach that combines the idea of direct relaxation spectrum identification with the optimal smoothing of the spectrum model is one of the directions of future studies.

The idea of direct spectrum identification can be transferred into the retardation spectrum identification based on the creep experiment data; however, some modifications are needed. The challenge that determines the direction of future research is to transfer the concept of relaxation spectrum identification with a quality index referring directly to the measurement-inaccessible spectrum to the task of spectrum recovery from the oscillatory shear data, i.e., when only the storage and/or loss moduli data are accessible for identification.

## Figures and Tables

**Table 1:** For uni-mode Gauss spectrum $H\left(\tau \right)$ (32) and the models ${\bar{H}}_{K}^{M}\left(v\right)$ (28),$\;{\bar{H}}_{K}\left(v\right)$ (29), ${\bar{H}}_{K}\left(\tau \right)$ (30): numbers of model summands K, time-scale factors α, optimal MRI rule (58) regularization parameters $\lambda^{*}$, optimal values $G_{K}\left(\lambda^{*}\right)$ of the index (52), the smoothness indices $\|{\bar{H}}_{K}^{M}\left(v\right){\|}_{2}=\|{\bar{H}}_{K}\left(\tau \right){\|}_{2}$ (73) and $\|{\bar{H}}_{K}{\left(v\right)\|}_{2}$ (74), the mean square relaxation modulus approximation index $Q_{K}\left({\bar{g}}_{K}\left(\lambda^{*}\right)\right)$ (45), norms $\|{\bar{g}}_{K}\left(\lambda^{*}\right){\|}_{2}$ (50) of the model parameter, and the integral square approximation indices $J\left({\bar{g}}_{K}\left(\lambda^{*}\right)\right)$ (75).

K	α $\;\left[s\right]$	$\lambda^{*}$ $\;\left[s^{-1}\right]$	$G_{K}\left(\lambda^{*}\right)$ $\left[kPa^{3}\cdot s^{1/2}\right]$	$\|{\bar{H}}_{K}\left(\tau \right){\|}_{2}$ $\;\left[kPa\cdot s^{1/2}\right]$	$\|{\bar{H}}_{K}\left(v\right){\|}_{2}$ $\;\left[kPa\cdot s^{-1/2}\right]$	$Q_{K}\left({\bar{g}}_{K}\left(\lambda \right)\right)$ $\;\left[kPa^{2}\right]$	$\|{\bar{g}}_{K}\left(\lambda \right){\|}_{2}$ $\;\left[kPa\cdot s\right]$	$J\left({\bar{g}}_{K}\left(\lambda \right)\right)$ $\left[kPa^{2}\cdot s\right]$
20	4.90	5.59540 × 10^−6^	3.013279 × 10^−4^	8.159207	0.974949	3.693104 × 10^−5^	4.857128 × 10^3^	4.197619
50	3.75	1.34875 × 10^−5^	2.842363 × 10^−4^	8.079248	0.849103	3.518103 × 10^−5^	3.109624 × 10^3^	2.650137
100	3.98	2.58751 × 10^−5^	2.653418 × 10^−4^	8.001643	0.891247	3.315447 × 10^−5^	2.225302 × 10^3^	3.635707
150	3.75	3.9961 × 10^−5^	2.669496 × 10^−4^	7.940807	0.888169	3.361745 × 10^−5^	1.777017 × 10^3^	4.359678
200	4.08	5.22487 × 10^−5^	2.685191 × 10^−4^	8.007734	0.929681	3.353246 × 10^−5^	1.567372 × 10^3^	4.524643

**Table 2:** For uni-mode Gauss spectrum $H\left(\tau \right)$ (32) and the models ${\bar{H}}_{K}^{M}\left(v\right)$ (28),$\;{\bar{H}}_{K}\left(v\right)$ (29) and ${\bar{H}}_{K}\left(\tau \right)$ (30): numbers of model summands K, time-scale factors α, optimal regularization parameters $\lambda^{**}$ determined according to the SMRI rule (59), optimal values $J_{K}\left(\lambda^{**}\right)$ of SMRI $G_{K}\left(\lambda \right)$ (53), the smoothness indices $\|{\bar{H}}_{K}^{M}\left(v\right){\|}_{2}=\|{\bar{H}}_{K}\left(\tau \right){\|}_{2}$ (73) and $\|{\bar{H}}_{K}\left(v\right){\|}_{2}$ (74), the mean square relaxation modulus approximation index $Q_{K}\left({\bar{g}}_{K}\left(\lambda^{**}\right)\right)$ (45), norms $\|{\bar{g}}_{K}\left(\lambda^{**}\right){\|}_{2}$ (50) of the model parameter, and the integral square approximation indices $J\left({\bar{g}}_{K}\left(\lambda^{**}\right)\right)$ (75).

K	α $\;\left[s\right]$	$\lambda^{**}$ $\;\left[s^{-1}\right]$	$J_{K}\left(\lambda^{**}\right)$ $\left[kPa^{4}\cdot s\right]$	$\|{\bar{H}}_{K}\left(\tau \right){\|}_{2}$ $\;\left[kPa\cdot s^{1/2}\right]$	$\|{\bar{H}}_{K}\left(v\right){\|}_{2}$ $\;\left[kPa\cdot s^{-1/2}\right]$	$Q_{K}\left({\bar{g}}_{K}\left(\lambda \right)\right)$ $\;\left[kPa^{2}\right]$	$\|{\bar{g}}_{K}\left(\lambda \right){\|}_{2}$ $\;\left[kPa\cdot s\right]$	$J\left({\bar{g}}_{K}\left(\lambda \right)\right)$ $\left[kPa^{2}\cdot s\right]$
20	4.90	1.11898 × 10^−5^	2.453706 × 10^−3^	8.129988	0.962135	3.712297 × 10^−5^	2.435084 × 10^3^	4.876315
50	3.75	2.81728 × 10^−5^	2.278118 × 10^−3^	7.971443	0.872795	3.585108 × 10^−5^	1.502817 × 10^3^	3.614060
100	3.98	5.34475 × 10^−5^	2.112612 × 10^−3^	7.929903	0.917628	3.359571 × 10^−5^	1.084462 × 10^3^	4.779057
150	3.75	8.26183 × 10^−5^	2.108808 × 10^−3^	7.866855	0.913183	3.407491 × 10^−5^	0.865339 × 10^3^	5.504216
200	4.08	1.10091 × 10^−4^	2.095602 × 10^−3^	7.855564	0.918668	3.395894 × 10^−5^	0.7485848 × 10^3^	5.749361

**Table 3:** For double-mode Gauss spectrum $H\left(\tau \right)$ (36) and the models ${\bar{H}}_{K}^{M}\left(v\right)$ (28),$\;{\bar{H}}_{K}\left(v\right)$ (29) and ${\bar{H}}_{K}\left(\tau \right)$ (30): numbers of model summands K, time-scale factors α, optimal regularization parameters $\lambda^{*}$ determined according to MRI rule (58), optimal values $G_{K}\left(\lambda^{*}\right)$ of MRI $G_{K}\left(\lambda \right)$ (52), the smoothness indices $\|{\bar{H}}_{K}^{M}\left(v\right){\|}_{2}=\|{\bar{H}}_{K}\left(\tau \right){\|}_{2}$ (73) and $\|{\bar{H}}_{K}\left(v\right){\|}_{2}$ (74), the mean square relaxation modulus approximation indices $Q_{K}\left({\bar{g}}_{K}\left(\lambda^{*}\right)\right)$ (45), norms $\|{\bar{g}}_{K}\left(\lambda^{*}\right){\|}_{2}$ (50), and the integral square approximation indices $J\left({\bar{g}}_{K}\left(\lambda^{*}\right)\right)$ (75).

K	α $\;\left[s\right]$	$\lambda^{*}$ $\;\left[s^{-1}\right]$	$G_{K}\left(\lambda^{*}\right)$ $\left[Pa^{3}\cdot s^{1/2}\right]$	$\|{\bar{H}}_{K}{\left(\tau \right)\|}_{2}$ $\;\left[Pa\cdot s^{1/2}\right]$	$\|{\bar{H}}_{K}{\left(v\right)\|}_{2}$ $\;\left[Pa\cdot s^{-1/2}\right]$	$Q_{K}\left({\bar{g}}_{K}\left(\lambda^{*}\right)\right)$ $\;\left[Pa^{2}\right]$	$\|{\bar{g}}_{K}{\left(\lambda^{*}\right)\|}_{2}$ $\left[Pa\cdot s\right]$	$J\left({\bar{g}}_{K}\left(\lambda^{*}\right)\right)$ $\left[Pa^{2}\cdot s\right]$
50	22.5	7.64987 × 10^−7^	1.515858 × 10^−4^	17.046243	0.378227	8.892620 × 10^−6^	2.756423 × 10^4^	82.965797
100	16.3	1.475563 × 10^−6^	1.373569 × 10^−4^	16.696517	0.406747	8.226679 × 10^−6^	1.943813 × 10^4^	90.388169
150	9.35	2.408194 × 10^−6^	1.344844 × 10^−4^	16.119592	0.436842	8.342914 × 10^−6^	1.468970 × 10^4^	91.374527
200	6.5	3.100806 × 10^−6^	1.379494 × 10^−4^	16.446934	0.404089	8.387543 × 10^−6^	1.320863 × 10^4^	85.712215
300	5.2	4.372258 × 10^−6^	1.339584 × 10^−4^	16.625818	0.398369	8.38689 × 10^−6^	1.27654 × 10^4^	88.211903

**Table 4:** For double-mode Gauss spectrum $H\left(\tau \right)$ (36) and the models ${\bar{H}}_{K}^{M}\left(v\right)$ (28),$\;{\bar{H}}_{K}\left(v\right)$ (29) and ${\bar{H}}_{K}\left(\tau \right)$ (30): numbers of model summands K, time-scale factors α, optimal regularization parameters $\lambda^{**}$ determined according to SMRI rule (59), optimal values $J_{K}\left(\lambda^{**}\right)$ of SMRI $G_{K}\left(\lambda \right)$ (53), the smoothness indices $\|{\bar{H}}_{K}^{M}\left(v\right){\|}_{2}=\|{\bar{H}}_{K}\left(\tau \right){\|}_{2}$ (73) and $\|{\bar{H}}_{K}\left(v\right){\|}_{2}$ (74), the mean square relaxation modulus approximation errors $Q_{K}\left({\bar{g}}_{K}\left(\lambda^{**}\right)\right)$ (45), norms $\|{\bar{g}}_{K}\left(\lambda^{**}\right){\|}_{2}$ (50), and the integral square approximation indices $J\left({\bar{g}}_{K}\left(\lambda^{**}\right)\right)$ (75).

K	α $\;\left[s\right]$	$\lambda^{**}$ $\;\left[s^{-1}\right]$	$J_{K}\left(\lambda^{**}\right)$ $\left[Pa^{4}\cdot s\right]$	$\|{\bar{H}}_{K}{\left(\tau \right)\|}_{2}$ $\;\left[Pa\cdot s^{1/2}\right]$	$\|{\bar{H}}_{K}{\left(v\right)\|}_{2}$ $\;\left[Pa\cdot s^{-1/2}\right]$	$Q_{K}\left({\bar{g}}_{K}\left(\lambda^{*}\right)\right)$ $\;\left[Pa^{2}\right]$	$\|{\bar{g}}_{K}{\left(\lambda^{*}\right)\|}_{2}$ $\;\left[Pa\cdot s\right]$	$J\left({\bar{g}}_{K}\left(\lambda^{*}\right)\right)$ $\left[Pa^{2}\cdot s\right]$
50	22.5	1.7453 × 10^−6^	2.529070 × 10^−3^	16.407358	0.373189	9.394714 × 10^−6^	1.241814 × 10^4^	89.601861
100	16.3	3.0764 × 10^−6^	2.278727 × 10^−3^	16.497668	0.392429	8.372346 × 10^−6^	9.405476 × 10^3^	93.292315
150	9.35	5.002 × 10^−6^	2.155531 × 10^−3^	15.944346	0.426699	8.478939 × 10^−6^	7.129723 × 10^3^	95.515503
200	6.5	6.7275 × 10^−6^	2.239574 × 10^−3^	16.063379	0.397277	8.679437 × 10^−6^	6.193085 × 10^3^	90.040123
300	5.2	9.21601 × 10^−6^	2.209601 × 10^−3^	16.376495	0.386739	8.238949 × 10^−6^	5.394711 × 10^3^	90.560338

**Table 5:** For KWW spectrum $H\left(\tau \right)$ (39) and the models ${\bar{H}}_{K}^{M}\left(v\right)$ (28),$\;{\bar{H}}_{K}\left(v\right)$ (29) and ${\bar{H}}_{K}\left(\tau \right)$ (30): numbers of model summands K, time-scale factors α, optimal regularization parameters $\lambda^{*}$ determined according to the MRI rule (58), optimal values $G_{K}\left(\lambda^{*}\right)$ of MRI $G_{K}\left(\lambda \right)$ (52), the smoothness indices $\|{\bar{H}}_{K}^{M}\left(v\right){\|}_{2}=\|{\bar{H}}_{K}\left(\tau \right){\|}_{2}$ (73) and $\|{\bar{H}}_{K}\left(v\right){\|}_{2}$ (74), the mean square relaxation modulus approximation indices $Q_{K}\left({\bar{g}}_{K}\left(\lambda^{*}\right)\right)$ (45), norms $\|{\bar{g}}_{K}\left(\lambda^{*}\right){\|}_{2}$ (50), and the integral square approximation indices $J\left({\bar{g}}_{K}\left(\lambda^{*}\right)\right)$ (75).

K	α $\;\left[s\right]$	$\lambda^{*}$ $\;\left[s^{-1}\right]$	$G_{K}\left(\lambda^{*}\right)$ $\left[MPa^{3}\cdot s^{1/2}\right]$	$\|{\bar{H}}_{K}\left(\tau \right){\|}_{2}$ $\;\left[MPa\cdot s^{1/2}\right]$	$\|{\bar{H}}_{K}\left(v\right){\|}_{2}$ $\;\left[MPa\cdot s^{-1/2}\right]$	$Q_{K}\left({\bar{g}}_{K}\left(\lambda \right)\right)$ $\;\left[MPa^{2}\right]$	$\|{\bar{g}}_{K}\left(\lambda \right){\|}_{2}$ $\;\left[MPa\cdot s\right]$	$J\left({\bar{g}}_{K}\left(\lambda \right)\right)$ $\left[MPa^{2}\cdot s\right]$
25	0.8	5.54090 × 10^−6^	4.281113 × 10^−8^	0.458183	0.280157	9.343671 × 10^−8^	2.758344 × 10^2^	3.613509 × 10^−8^
50	0.65	9.78240 × 10^−6^	3.897311 × 10^−8^	0.463474	0.294728	8.408914 × 10^−8^	2.096088 × 10^2^	5.893675 × 10^−8^
75	0.6	1.51217 × 10^−5^	3.884095 × 10^−8^	0.458345	0.292681	8.474179 × 10^−8^	1.667166 × 10^2^	1.790198 × 10^−8^
100	0.65	1.91636 × 10^−5^	3.717906 × 10^−8^	0.459359	0.285467	8.093675 × 10^−8^	1.484553 × 10^2^	2.480823 × 10^−8^
150	0.6	2.94430 × 10^−5^	3.782735 × 10^−8^	0.457921	0.289998	8.371472 × 10^−8^	1.243022 × 10^2^	1.282906 × 10^−8^
200	0.6	3.90737 × 10^−5^	3.759169 × 10^−8^	0.458133	0.290078	8.205409 × 10^−8^	1.036765 × 10^2^	1.678857 × 10^−8^
300	0.55	5.69379 × 10^−5^	3.639980 × 10^−8^	0.457682	0.295626	7.953070 × 10^−8^	85.788011	1.130750 × 10^−8^
400	0.55	7.73969 × 10^−5^	3.699510 × 10^−8^	0.457289	0.295293	8.090095 × 10^−8^	73.499308	1.016209 × 10^−8^

**Table 6:** For KWW spectrum $H\left(\tau \right)$ (39) and the models ${\bar{H}}_{K}^{M}\left(v\right)$ (28),$\;{\bar{H}}_{K}\left(v\right)$ (29) and ${\bar{H}}_{K}\left(\tau \right)$ (30): numbers of model summands K, time-scale factors α, optimal regularization parameters $\lambda^{**}$ determined according to the SMRI rule (59), optimal values $J_{K}\left(\lambda^{**}\right)$ of SMRI $G_{K}\left(\lambda \right)$ (53), the smoothness indices $\|{\bar{H}}_{K}^{M}\left(v\right){\|}_{2}=\|{\bar{H}}_{K}\left(\tau \right){\|}_{2}$ (73) and $\|{\bar{H}}_{K}\left(v\right){\|}_{2}$ (74), the mean square relaxation modulus approximation indices $Q_{K}\left({\bar{g}}_{K}\left(\lambda^{**}\right)\right)$ (45), norms $\|{\bar{g}}_{K}{\left(\lambda^{**}\right)\|}_{2}$ (50), and the integral square approximation indices $J\left({\bar{g}}_{K}\left(\lambda^{**}\right)\right)$ (75).

K	α $\;\left[s\right]$	$\lambda^{**}$ $\;\left[s^{-1}\right]$	$J_{K}\left(\lambda^{**}\right)$ $\left[MPa^{4}\cdot s\right]$	$\|{\bar{H}}_{K}{\left(\tau \right)\|}_{2}$ $\;\left[MPa\cdot s^{1/2}\right]$	$\|{\bar{H}}_{K}\left(v\right){\|}_{2}$ $\;\left[MPa\cdot s^{-1/2}\right]$	$Q_{K}\left({\bar{g}}_{K}\left(\lambda \right)\right)$ $\;\left[MPa^{2}\right]$	$\|{\bar{g}}_{K}\left(\lambda \right){\|}_{2}$ $\;\left[MPa\cdot s\right]$	$J\left({\bar{g}}_{K}\left(\lambda \right)\right)$ $\left[MPa^{2}\cdot s\right]$
25	0.8	1.13293 × 10^−5^	1.954304 × 10^−8^	0.455582	0.268909	9.415834 × 10^−8^	1.354242 × 10^2^	2.047282 × 10^−3^
50	0.65	2.00618 × 10^−5^	1.798098 × 10^−8^	0.459997	0.285329	8.497718 × 10^−8^	1.027463 × 10^2^	2.833885 × 10^−3^
75	0.6	3.04410 × 10^−5^	1.778439 × 10^−8^	0.457604	0.289663	8.492972 × 10^−8^	82.908976	1.111339 × 10^−3^
100	0.65	3.88242 × 10^−5^	1.704248 × 10^−8^	0.457753	0.284658	8.133359 × 10^−8^	73.456875	1.300175 × 10^−3^
150	0.6	5.91834 × 10^−5^	1.732882 × 10^−8^	0.457403	0.289823	8.383839 × 10^−8^	61.884452	8.912376 × 10^−4^
200	0.6	7.87442 × 10^−5^	1.719985 × 10^−8^	0.457169	0.289616	8.229464 × 10^−8^	51.520752	9.747906 × 10^−48^
300	0.55	1.143728 × 10^−4^	1.664771 × 10^−8^	0.457116	0.295142	7.967139 × 10^−8^	42.745369	8.056422 × 10^−4^
400	0.55	1.55291 × 10^−4^	1.690685 × 10^−8^	0.456807	0.294910	8.102074 × 10^−8^	36.659148	7.196253 × 10^−4^

## Data Availability

The original contributions presented in the study are included in the article and Supplementary Materials; further inquiries can be directed to the first author.

## Supplementary Materials

The following supporting information can be downloaded at: https://www.mdpi.com/article/10.3390/polym17010031/s1, Supplementary Materials, Section S.1. Applicability of GCV and L-Curve Methods to Direct Relaxation Spectrum Identification; Section S.2. Plots of the Multiplicative Regularization Indices; Section S.3. Plots of the Optimal Models of the KWW Relaxation Spectra.

## Appendix A

### Appendix A.1. Derivation of the Formula (64)

Combining (60) and (61), we have

$$\frac{dG_{K}\left(\lambda \right)}{d\lambda }=\frac{\alpha^{3}\lambda \left[\sum_{k=1}^{K}\frac{y_{k}^{2}\sigma_{k}}{{\left(\sigma_{k}+\alpha \lambda \right)}^{3}}\right]\left[\sum_{k=1}^{K}\;\frac{\left(2\sigma_{k}-\alpha \lambda \right)y_{k}^{2}}{{\left(\sigma_{k}+\alpha \lambda \right)}^{2}}\right]}{K\sqrt{\sum_{k=1}^{K}\;\frac{\alpha \sigma_{k}y_{k}^{2}}{{\left(\sigma_{k}+\alpha \lambda \right)}^{2}}}},$$

whence we obtain the next equation

$$\frac{d^{2}G_{K}\left(\lambda \right)}{d\lambda^{2}}=\frac{\alpha^{5}}{K}\lambda \frac{{\left[\sum_{k=1}^{K}\frac{y_{k}^{2}\sigma_{k}}{{\left(\sigma_{k}+\alpha \lambda \right)}^{3}}\right]}^{2}\left[\sum_{k=1}^{K}\;\frac{\left(2\sigma_{k}-\alpha \lambda \right)y_{k}^{2}}{{\left(\sigma_{k}+\alpha \lambda \right)}^{2}}\right]}{\sqrt{\sum_{k=1}^{K}\;\frac{\alpha \sigma_{k}y_{k}^{2}}{{\left(\sigma_{k}+\alpha \lambda \right)}^{2}}}\left[\sum_{k=1}^{K}\;\frac{\alpha \sigma_{k}y_{k}^{2}}{{\left(\sigma_{k}+\alpha \lambda \right)}^{2}}\right]}+\frac{\alpha^{3}\left[\sum_{k=1}^{K}\frac{y_{k}^{2}\sigma_{k}}{{\left(\sigma_{k}+\alpha \lambda \right)}^{3}}\right]\left[\sum_{k=1}^{K}\;\frac{\left(2\sigma_{k}-\alpha \lambda \right)y_{k}^{2}}{{\left(\sigma_{k}+\alpha \lambda \right)}^{2}}\right]}{K\sqrt{\sum_{k=1}^{K}\;\frac{\alpha \sigma_{k}y_{k}^{2}}{{\left(\sigma_{k}+\alpha \lambda \right)}^{2}}}}+\;\frac{\alpha^{3}}{K}\lambda \frac{\left\{-3\alpha \left[\sum_{k=1}^{K}\frac{y_{k}^{2}\sigma_{k}}{{\left(\sigma_{k}+\alpha \lambda \right)}^{4}}\right]\left[\sum_{k=1}^{K}\;\frac{\left(2\sigma_{k}-\alpha \lambda \right)y_{k}^{2}}{{\left(\sigma_{k}+\alpha \lambda \right)}^{2}}\right]+\left[\sum_{k=1}^{K}\frac{y_{k}^{2}\sigma_{k}}{{\left(\sigma_{k}+\alpha \lambda \right)}^{3}}\right]\left[\sum_{k=1}^{K}\;\frac{\alpha y_{k}^{2}}{{\left(\sigma_{k}+\alpha \lambda \right)}^{2}}-\sum_{k=1}^{K}\;\frac{\left(6\sigma_{k}\right)\alpha y_{k}^{2}}{{\left(\sigma_{k}+\alpha \lambda \right)}^{3}}\right]\right\}}{\sqrt{\sum_{k=1}^{K}\;\frac{\alpha \sigma_{k}y_{k}^{2}}{{\left(\sigma_{k}+\alpha \lambda \right)}^{2}}}},$$

which, having in mind the notation (61), can be rewritten as follows

$$\frac{d^{2}G_{K}\left(\lambda \right)}{d\lambda^{2}}=\frac{\alpha^{3}\left[\sum_{k=1}^{K}\frac{y_{k}^{2}\sigma_{k}}{{\left(\sigma_{k}+\alpha \lambda \right)}^{3}}\right]G\left(\lambda \right)}{K\sqrt{\sum_{k=1}^{K}\;\frac{\alpha \sigma_{k}y_{k}^{2}}{{\left(\sigma_{k}+\alpha \lambda \right)}^{2}}}}+\frac{\alpha^{4}}{K}\lambda \frac{\left\{-3\alpha \left[\sum_{k=1}^{K}\frac{y_{k}^{2}\sigma_{k}}{{\left(\sigma_{k}+\alpha \lambda \right)}^{4}}\right]G\left(\lambda \right)+\left[\sum_{k=1}^{K}\frac{y_{k}^{2}\sigma_{k}}{{\left(\sigma_{k}+\alpha \lambda \right)}^{3}}\right]\left[\sum_{k=1}^{K}\;\frac{y_{k}^{2}}{{\left(\sigma_{k}+\alpha \lambda \right)}^{2}}-\sum_{k=1}^{K}\;\frac{6\sigma_{k}y_{k}^{2}}{{\left(\sigma_{k}+\alpha \lambda \right)}^{3}}\right]\right\}}{\sqrt{\sum_{k=1}^{K}\;\frac{\alpha \sigma_{k}y_{k}^{2}}{{\left(\sigma_{k}+\alpha \lambda \right)}^{2}}}}+\;\frac{\alpha^{4}}{K}\lambda \frac{{\left[\sum_{k=1}^{K}\frac{y_{k}^{2}\sigma_{k}}{{\left(\sigma_{k}+\alpha \lambda \right)}^{3}}\right]}^{2}G\left(\lambda \right)}{\sqrt{\sum_{k=1}^{K}\;\frac{\alpha \sigma_{k}y_{k}^{2}}{{\left(\sigma_{k}+\alpha \lambda \right)}^{2}}}\left[\sum_{k=1}^{K}\;\frac{\sigma_{k}y_{k}^{2}}{{\left(\sigma_{k}+\alpha \lambda \right)}^{2}}\right]},$$

and is equivalent to (64).

### Appendix A.2. Derivation of the Formula (71)

Equations (67) and (68) imply

$$\frac{dJ_{K}\left(\lambda \right)}{d\lambda }=\frac{2\alpha^{3}}{K}\lambda \left[\sum_{k=1}^{K}\;\frac{\sigma_{k}y_{k}^{2}}{{\left(\sigma_{k}+\alpha \lambda \right)}^{3}}\right]\left[\sum_{k=1}^{K}\;\frac{\left(\sigma_{k}-\alpha \lambda \right)y_{k}^{2}}{{\left(\sigma_{k}+\alpha \lambda \right)}^{2}}\right],$$

whence the second derivative is as follows

$$\frac{d^{2}J_{K}\left(\lambda \right)}{d\lambda^{2}}=\frac{2\alpha^{3}}{K}\left[\sum_{k=1}^{K}\;\frac{\sigma_{k}y_{k}^{2}}{{\left(\sigma_{k}+\alpha \lambda \right)}^{3}}\right]\left[\sum_{k=1}^{K}\;\frac{\left(\sigma_{k}-\alpha \lambda \right)y_{k}^{2}}{{\left(\sigma_{k}+\alpha \lambda \right)}^{2}}\right]+\frac{2\alpha^{3}}{K}\lambda \left\{-3\alpha \left[\sum_{k=1}^{K}\;\frac{\sigma_{k}y_{k}^{2}}{{\left(\sigma_{k}+\alpha \lambda \right)}^{4}}\right]\left[\sum_{k=1}^{K}\;\frac{\left(\sigma_{k}-\alpha \lambda \right)y_{k}^{2}}{{\left(\sigma_{k}+\alpha \lambda \right)}^{2}}\right]+\;\left[\sum_{k=1}^{K}\;\frac{\sigma_{k}y_{k}^{2}}{{\left(\sigma_{k}+\alpha \lambda \right)}^{3}}\right]\left[\sum_{k=1}^{K}\;\frac{\alpha y_{k}^{2}}{{\left(\sigma_{k}+\alpha \lambda \right)}^{2}}-\sum_{k=1}^{K}\;\frac{4\alpha \sigma_{k}y_{k}^{2}}{{\left(\sigma_{k}+\alpha \lambda \right)}^{3}}\right]\right\},$$

and, after algebraic manipulations, having in mind the notation (68), can be written as

$$\frac{d^{2}J_{K}\left(\lambda \right)}{d\lambda^{2}}=\frac{2\alpha^{3}}{K}\left[\sum_{k=1}^{K}\;\frac{\sigma_{k}y_{k}^{2}}{{\left(\sigma_{k}+\alpha \lambda \right)}^{3}}\right]F\left(\lambda \right)+\frac{2\alpha^{4}}{K}\lambda \left\{-3\left[\sum_{k=1}^{K}\;\frac{\sigma_{k}y_{k}^{2}}{{\left(\sigma_{k}+\alpha \lambda \right)}^{4}}\right]F\left(\lambda \right)+\left[\sum_{k=1}^{K}\;\frac{\sigma_{k}y_{k}^{2}}{{\left(\sigma_{k}+\alpha \lambda \right)}^{3}}\right]\left[\sum_{k=1}^{K}\;\frac{\left(-3\sigma_{k}+\alpha \lambda \right)y_{k}^{2}}{{\left(\sigma_{k}+\alpha \lambda \right)}^{3}}\right]\right\},$$

whence Equation (71) directly results.

## Appendix B

### Appendix B.1. Symbols

$G\left(t\right)$	-linear relaxation modulus, Equations (1) and (2), Pa
$G\left(t_{k}\right)$	-relaxation modulus obtained in stress relaxation test, k=1,…,K, Pa
$\bar{G}\left(t_{k}\right)$	-noise corrupted measurement of the modulus $G\left(t_{k}\right)$, k=1,…,K, Pa
$G_{K}\left(t\right)$	-relaxation modulus model, Equation (10), Pa
${\bar{G}}_{K}\left(t\right)$	-optimal model of relaxation modulus, Equation (31), Pa
${\bar{G}}_{K}$	-K dimensional vector of the measurements $\bar{\bar{G}}\left(t_{k}\right)$, Equation (19)
$G_{K}$	-N dimensional vector of noise-free measurements $G\left(t_{k}\right)$, below Equation (75)
$g_{k}$	-parameters of the models $H_{K}\left(v\right)$ $H_{K}^{M}\left(v\right)$, $H_{K}\left(\tau \right)$ and $G_{K}\left(t\right)$, Pa·s
$g_{K}$	-vector of parameters $g_{k}$ of models $H_{K}\left(v\right)$ $H_{K}^{M}\left(v\right)$, $H_{K}\left(\tau \right)$ and $G_{K}\left(t\right)$ defined below Equation (15)
${\bar{g}}_{K}\left(\lambda \right)$	-optimal regularized model parameter vector, Equation (22)
$G\left(\lambda \right)$, $F\left(\lambda \right)$	-auxiliary functions introduced in Equations (61) and (68)
$H\left(\tau \right)$	-continuous spectrum of relaxation times τ, Equation (1), Pa
$H_{K}\left(\tau \right)$	-model of the spectrum $H\left(\tau \right)$, Equation (12), Pa
${\bar{H}}_{K}\left(\tau \right)$	-optimal model of the spectrum $H\left(\tau \right)$, Equation (30), Pa
$H\left(v\right)$	-continuous spectrum of relaxation frequencies v, Equation (2), Pa
$H_{K}\left(v\right)$	-model of the spectrum $H\left(v\right)$, Equation (9), Pa
${\bar{H}}_{K}\left(v\right)$	-optimal model of the spectrum $H\left(v\right)$, Equation (29), Pa
$H^{M}\left(v\right)$	-modified relaxation spectrum, Equation (4), Pa·s
$H_{K}^{M}\left(v\right)$	-model of the modified relaxation spectrum $H^{M}\left(v\right)$, Equation (8), Pa·s
${\bar{H}}_{K}^{M}\left(v\right)$	-optimal model of modified spectrum $H^{M}\left(v\right)$, Equation (28), Pa·s
$h_{k}\left(v\right)$	-exponential basis functions of the model $H_{K}^{M}\left(v\right)$, Equation (7), –
$h_{k}\left(\tau \right)$	-basis functions of the model $H_{K}\left(\tau \right)$, Equation (13), $s^{-1}$
$I_{K}$	-K×K identity matrix
$J\left(g_{K}\right)$	-integral square relaxation spectra approximation index, Equation (15), ${\mathrm{Pa}}^{2}\cdot s$
${\bar{J}}_{K}\left(g_{K}\right)$	-integral-empirical identification index, Equation (18), ${\mathrm{Pa}}^{2}\cdot s$
$∆{\bar{J}}_{K}\left({\bar{g}}_{K}\left(\lambda \right)\right)$	-increment of index ${\bar{J}}_{K}\left(g_{K}\right)$ for optimal model parameter, Equation (49), ${\mathrm{Pa}}^{2}\cdot s$
K	-number of models $H_{K}\left(v\right)$ $H_{K}^{M}\left(v\right)$, $H_{K}\left(\tau \right)$ and $G_{K}\left(t\right)$ summands and number of measurements, –
$Q_{K}\left({\bar{g}}_{K}\left(\lambda \right)\right)$	-mean square error of the relaxation modulus model ${\bar{G}}_{K}\left(t\right)$, Equation (45), Pa^2^
$t_{k}$	-sampling instants used in the stress relaxation test, $t_{k}=\alpha k,$ k=1,…,K, s
$U_{K}$	-K×K orthogonal matrix introduced by SVD of $\Phi_{K}$, Equation (23)
$Y_{K}$	-K dimensional vector $U_{K}^{T}{\bar{G}}_{K}$, Equation (27)
$z\left(t_{k}\right)$	-additive noise of relaxation modulus measurement, k=1,…,K, Pa
α	-time-scaling factor of the basis functions $h_{k}\left(v\right)$ and $h_{k}\left(\tau \right)$, s
$\kappa \left(\Phi_{K}\right)$	-spectral conditional number of matrix $\Phi_{K}$, Equation (72)
λ	-regularization parameter introduced in the optimization task (21), $s^{-1}$
$\lambda^{*}$	-optimal regularization parameter according MRI rule, Equation (58), $s^{-1}$
$\lambda^{**}$	-optimal regularization parameter according SMRI rule, Equation (59), $s^{-1}$
$\sigma_{k}$	-non-zero singular values of $\Phi_{K}$, k=1,…,K introduced by SVD (23)
$\Sigma_{K}$	-K×K diagonal matrix of singular values $\sigma_{1},\ldots ,\sigma_{K}$ of $\Phi_{K}$, Equation (24)
$\Phi_{K}$	-K×K matrix defined by (19) basic for regularized direct relaxation spectra identification task (21)
$\phi_{k}\left(t\right)$	-basis functions of the model $G_{K}\left(t\right)$, Equation (11), $s^{-1}$
$\Omega_{K}\left(\lambda \right)$	-diagonal K×K matrix function of regularization parameter, Equation (26)
$G_{K}\left(\lambda \right)$	-multiplicative regularization index MRI, Equation (52), ${\mathrm{Pa}}^{3}\cdot s^{1/2}$
$J_{K}\left(\lambda \right)$	-square multiplicative regularization index SMRI, Equation (53), ${\mathrm{Pa}}^{4}\cdot s$

### Appendix B.2. Mathematical Terminology and Special Functions

$erfc\left(x\right)$	-complementary error function
$L^{2}\left(0,\right.\left.\infty \right)$	-space of real-valued square-integrable functions on the interval $\left(0,\right.\left.\infty \right)$
$\Gamma \left(n\right)$	-Euler’s gamma function
$\|\cdot {\|}_{2}$	-square norm in the real Euclidean space $R^{K}$ and in $L^{2}\left(0,\right.\left.\infty \right)$
$\underset{x}{\mathrm{local}\;\min}f\left(x\right)$	-find the value *x* at which the function $f\left(x\right)$ attains a local minimum, Equation (58)
$\underset{x}{\arg\;\mathrm{local}\;\min}f\left(x\right)$	-the value of x at which the function $f\left(x\right)$ attains a local minimum, Equation (56)

### Appendix B.3. Acronyms

MRI	-multiplicative regularization index $G_{K}\left(\lambda \right)$, Equation (52), ${\mathrm{Pa}}^{3}\cdot s^{1/2}$
SMRI	-square multiplicative regularization index $J_{K}\left(\lambda \right)$, Equation (53), ${\mathrm{Pa}}^{4}\cdot s$
SVD	-singular value decomposition, Equation (23)
SCNR	-Spectral Condition Number Rule, Equation (72)
