# Supplementary material for: Optimal Choice of the Regularization Parameter for Direct Identification of Polymers Relaxation Time and Frequency Spectra
Source: Polymers (Basel). 2024 Dec 26;17(1):31. doi: 10.3390/polym17010031 (PMC11722655; doi:10.3390/polym17010031)
Supplement: Supplementary file 1 [file polymers-17-00031-s001.zip › Supplementary materials 26.12.2024.pdf]

polymers

MDPI

Article

Optimal Choice of the Regularization Parameter for Direct Identification of Polymers Relaxation Time and Frequency Spectra

Anna Stankiewicz and Monika Bojanowska

S.1. Applicability of GCV and L-Curve Methods to Direct Relaxation Spectrum Identification

S.1.1. Direct Spectrum Identification as a Least-Squares Problem

According to Lemma 1 in [24], matrix $\boldsymbol{\Phi}_K$ is the Gram matrix, i.e., can be represented as

$$\boldsymbol{\Phi}_K = \boldsymbol{\Psi}_K^T \boldsymbol{\Psi}_K, \tag{S1}$$

where matrix $\boldsymbol{\Psi}_K \in R^{K,K}$ is also non-singular. In general, the factorization (S1) of $\boldsymbol{\Phi}_K$ is not unique. However, by the SVD decomposition (23) and the orthogonality of $\boldsymbol{U}_K$, we have

$$\boldsymbol{\Phi}_K = \boldsymbol{\Psi}_K^T \boldsymbol{\Psi}_K = \boldsymbol{U}_K \boldsymbol{\Sigma}_K^{1/2} \boldsymbol{\Sigma}_K^{1/2} \boldsymbol{U}_K^T = \boldsymbol{U}_K \boldsymbol{\Sigma}_K^{1/2} \boldsymbol{U}_K^T \boldsymbol{U}_K \boldsymbol{\Sigma}_K^{1/2} \boldsymbol{U}_K^T,$$

where, according to (24), the square root of the diagonal matrix $\boldsymbol{\Sigma}_K$ is as follows

$$\boldsymbol{\Sigma}_K^{1/2} = diag(\sqrt{\sigma_1}, \dots, \sqrt{\sigma_K}), \tag{S2}$$

whence

$$\boldsymbol{\Psi}_K = \boldsymbol{U}_K \boldsymbol{\Sigma}_K^{1/2} \boldsymbol{U}_K^T. \tag{S3}$$

In view of (S1), the linear-quadratic regularized optimization task (21) can be expressed as

$$\min_{\boldsymbol{g}_K \in R^K} \ \frac{1}{\alpha} \boldsymbol{g}_K^T \boldsymbol{\Psi}_K^T \boldsymbol{\Psi}_K \boldsymbol{g}_K - 2\frac{1}{\sqrt{\alpha}} \overline{\overline{\boldsymbol{G}}}_K^T \boldsymbol{\Psi}_K \boldsymbol{g}_K + \lambda \boldsymbol{g}_K^T \boldsymbol{g}_K,$$

where the respective relaxation modulus experiment data

$$\overline{\overline{\boldsymbol{G}}}_K = \sqrt{\alpha}\, \boldsymbol{\Psi}_K^{-T} \overline{\boldsymbol{G}}_K, \tag{S4}$$

or, equivalently, as the standard regularized linear least-squares task

$$\min_{\boldsymbol{g}_K \in R^K} \ \left\| \overline{\overline{\boldsymbol{G}}}_K - \frac{1}{\sqrt{\alpha}} \boldsymbol{\Psi}_K \boldsymbol{g}_K \right\|_2^2 + \lambda \|\boldsymbol{g}_K\|_2^2. \tag{S5}$$

The vector of optimal model parameters $\overline{\boldsymbol{g}}_K(\lambda)$ (22) in terms of $\boldsymbol{\Psi}_K$ and $\overline{\overline{\boldsymbol{G}}}_K$ as as follows

$$\overline{\boldsymbol{g}}_K(\lambda) = \left(\frac{1}{\alpha} \boldsymbol{\Psi}_K^T \boldsymbol{\Psi}_K + \lambda \mathbb{I}_K\right)^{-1} \frac{1}{\sqrt{\alpha}} \boldsymbol{\Psi}_K^T \overline{\overline{\boldsymbol{G}}}_K = (\boldsymbol{\Psi}_K^T \boldsymbol{\Psi}_K + \alpha\lambda \mathbb{I}_K)^{-1} \sqrt{\alpha}\, \boldsymbol{\Psi}_K^T \overline{\overline{\boldsymbol{G}}}_K. \tag{S6}$$

The optimal $\overline{\boldsymbol{g}}_K(\lambda)$ (S6) is obviously identical with that described by formula (22).

S.1.2. Applicability of the Generalized Cross Validation

 https://doi.org/10.3390/polym17010031

The generalized cross validation (GCV) algorithm applied to the regularized problem (S5) relies on choosing that regularization parameter λ which minimizes the GCV functional [35,36]:

$$V_{GCV}(\lambda) = \frac{\|\boldsymbol{\varrho}(\lambda)\|_2^2}{tr[\Xi(\lambda)]^2}, \tag{S7}$$

where, for the task (S5), the square matrix is as follows

$$\Xi(\lambda) = \mathbb{I}_K - \frac{1}{\alpha}\boldsymbol{\Psi}_K\left(\frac{1}{\alpha}\boldsymbol{\Psi}_K^T\boldsymbol{\Psi}_K + \lambda\mathbb{I}_K\right)^{-1}\boldsymbol{\Psi}_K^T = \mathbb{I}_K - \boldsymbol{\Psi}_K(\boldsymbol{\Psi}_K^T\boldsymbol{\Psi}_K + \alpha\lambda\mathbb{I}_K)^{-1}\boldsymbol{\Psi}_K^T, \tag{S8}$$

and the residual vector $\boldsymbol{\varrho}(\lambda)$ for the regularized solution (S6) is given by

$$\boldsymbol{\varrho}(\lambda) = \Xi(\lambda)\overline{\overline{\boldsymbol{G}}}_K = \overline{\overline{\boldsymbol{G}}}_K - \frac{1}{\sqrt{\alpha}}\boldsymbol{\Psi}_K\overline{\boldsymbol{g}}_K(\lambda), \tag{S9}$$

where $tr[\Xi(\lambda)]$ denotes the trace of $\Xi(\lambda)$.

The problem of choosing the optimal regularization parameter takes the form

$$\lambda_{GCV} = \min\left\{\lambda: \lambda = \arg\min_{\lambda\geq 0} V_{GCV}(\lambda)\right\}.$$

In view of (S8) and (S1), $tr[\Xi(\lambda)]$ can be expressed as

$$tr[\Xi(\lambda)] = K - tr[(\boldsymbol{\Phi}_K + \alpha\lambda\mathbb{I}_K)^{-1}(\boldsymbol{\Phi}_K + \alpha\lambda\mathbb{I}_K) - \alpha\lambda(\boldsymbol{\Phi}_K + \alpha\lambda\mathbb{I}_K)^{-1}],$$

whence

$$tr[\Xi(\lambda)] = \alpha\lambda tr[(\boldsymbol{\Phi}_K + \alpha\lambda\mathbb{I}_K)^{-1}]. \tag{S10}$$

By the left equality in (S9), the right equality in (S8) and (S1), we obtain

$$\boldsymbol{\varrho}(\lambda) = \overline{\overline{\boldsymbol{G}}}_K - \boldsymbol{\Psi}_K(\boldsymbol{\Phi}_K + \alpha\lambda\mathbb{I}_K)^{-1}\boldsymbol{\Psi}_K^T\overline{\overline{\boldsymbol{G}}}_K,$$

whence, including (S4), the square of the residual vector $\boldsymbol{\varrho}(\lambda)$ norm is as follows

$$\|\boldsymbol{\varrho}(\lambda)\|_2^2 = \alpha\overline{\boldsymbol{G}}_K^T[\boldsymbol{\Psi}_K^{-1} - (\boldsymbol{\Phi}_K + \alpha\lambda\mathbb{I}_K)^{-1}\boldsymbol{\Psi}_K^T][\boldsymbol{\Psi}_K^{-T} - \boldsymbol{\Psi}_K(\boldsymbol{\Phi}_K + \alpha\lambda\mathbb{I}_K)^{-1}]\overline{\boldsymbol{G}}_K,$$

which, in view of (S1) and invertability of $\boldsymbol{\Phi}_K$, can be expressed as

$$\|\boldsymbol{\varrho}(\lambda)\|_2^2 = \alpha\overline{\boldsymbol{G}}_K^T\boldsymbol{\Theta}(\lambda)\overline{\boldsymbol{G}}_K, \tag{S11}$$

where square matrix

$$\boldsymbol{\Theta}(\lambda) = \boldsymbol{\Phi}_K^{-1} - 2(\boldsymbol{\Phi}_K + \alpha\lambda\mathbb{I}_K)^{-1} + (\boldsymbol{\Phi}_K + \alpha\lambda\mathbb{I}_K)^{-1}\boldsymbol{\Phi}_K(\boldsymbol{\Phi}_K + \alpha\lambda\mathbb{I}_K)^{-1}.$$

Multiplying the above equation on both sides by $(\boldsymbol{\Phi}_K + \alpha\lambda\mathbb{I}_K)$, we get

$$(\boldsymbol{\Phi}_K + \alpha\lambda\mathbb{I}_K)\boldsymbol{\Theta}(\lambda)(\boldsymbol{\Phi}_K + \alpha\lambda\mathbb{I}_K) = (\alpha\lambda)^2\boldsymbol{\Phi}_K^{-1},$$

which immediately yields

$$\boldsymbol{\Theta}(\lambda) = (\alpha\lambda)^2(\boldsymbol{\Phi}_K + \alpha\lambda\mathbb{I}_K)^{-1}\boldsymbol{\Phi}_K^{-1}(\boldsymbol{\Phi}_K + \alpha\lambda\mathbb{I}_K)^{-1}. \tag{S12}$$

Combining (S7), (S11), (S12) and (S10), the next formula is obtained

$$V_{GCV}(\lambda) = \frac{\alpha\overline{\boldsymbol{G}}_K^T(\boldsymbol{\Phi}_K+\alpha\lambda\mathbb{I}_K)^{-1}\boldsymbol{\Phi}_K^{-1}(\boldsymbol{\Phi}_K+\alpha\lambda\mathbb{I}_K)^{-1}\overline{\boldsymbol{G}}_K}{tr[(\boldsymbol{\Phi}_K+\alpha\lambda\mathbb{I}_K)^{-1}]^2}. \tag{S13}$$

Therefore, the GCV function $V_{GCV}(\lambda)$ (S7) of the least squares task (S5) can be uniquely expressed in terms of the original task (21) although the factorization (S1) is not unique.

By (S13), (24), and since, by (23) and (26), we have

$$(\boldsymbol{\Phi}_K + \alpha\lambda\mathbb{I}_K)^{-1} = \boldsymbol{U}_K(\boldsymbol{\Sigma}_K + \alpha\lambda\mathbb{I}_K)^{-1}\boldsymbol{U}_K^T = \boldsymbol{U}_K\boldsymbol{\Omega}_K(\lambda)\boldsymbol{U}_K^T, \tag{S14}$$

in view of (27) and orthogonality of $\boldsymbol{U}_K$, we obtain

$$V_{GCV}(\lambda) = \frac{\alpha \bar{\boldsymbol{G}}_K^T \boldsymbol{U}_K \boldsymbol{\Omega}_K(\lambda) \boldsymbol{\Sigma}_K^{-1} \boldsymbol{\Omega}_K(\lambda) \boldsymbol{U}_K^T \bar{\boldsymbol{G}}_K}{tr\left[\boldsymbol{U}_K \boldsymbol{\Omega}_K(\lambda) \boldsymbol{U}_K^T\right]^2} = \frac{\alpha \boldsymbol{Y}_K^T \boldsymbol{\Omega}_K(\lambda) \boldsymbol{\Sigma}_K^{-1} \boldsymbol{\Omega}_K(\lambda) \boldsymbol{Y}_K}{tr[\boldsymbol{\Omega}_K(\lambda)]^2} = \alpha \frac{\sum_{k=1}^K \frac{y_k^2}{\sigma_k(\sigma_k+\alpha\lambda)^2}}{\left[\sum_{k=1}^K \frac{1}{(\sigma_k+\alpha\lambda)}\right]^2},$$

where both the denominator and nominator of the right-hand side equation are strictly monotonically decreasing functions of the regularization parameter. The GCV functional $V_{GCV}(\lambda)$ (S13), equivalently given by the above formula, is a differentiable function of the regularization parameter λ.

Following [35,37], we consider for the regularized task (S5) the following condition, called the Discrete Picard Condition [37].

Condition 1. *The coefficients* $|\bar{\bar{y}}_k|$ *decay faster that the singular values* $\sqrt{\sigma_k}$ *of the matrix* $\boldsymbol{\Psi}_K$ *(S3), where* $\bar{\bar{y}}_k$ *are elements of the vector (compare (27))*

$$\bar{\bar{\boldsymbol{Y}}}_K = \boldsymbol{U}_K^T \bar{\bar{\boldsymbol{G}}}_K = \sqrt{\alpha} \boldsymbol{U}_K^T \boldsymbol{\Psi}_K^{-T} \bar{\boldsymbol{G}}_K = \sqrt{\alpha} \boldsymbol{\Sigma}_K^{-1/2} \boldsymbol{U}_K^T \bar{\boldsymbol{G}}_K = \sqrt{\alpha} \boldsymbol{\Sigma}_K^{-1/2} \boldsymbol{Y}_K. \quad \text{(S15)}$$

The successive equalities in (S15) follow from (S3), (S4) and (27). According to [37], this assumption ensures that there exists a physically meaningful solution to the underlying inverse problem and that the solution can be approximated by a regularized solution. The above assumption means, in particular, that $\bar{\bar{y}}_k^2/\sigma_k$ is decreasing (non-increasing) sequence, i.e., the left inequality in the following holds

$$\frac{\bar{\bar{y}}_k^2}{\sigma_k} \geq \frac{\bar{\bar{y}}_{k+1}^2}{\sigma_{k+1}} \geq \frac{\bar{\bar{y}}_{k+1}^2}{\sigma_k}, \quad \text{(S16)}$$

where, in turn, the right inequality is obvious for decreasing sequence $\{\sigma_k\}$ and implies

$$\bar{\bar{y}}_k^2 \geq \bar{\bar{y}}_{k+1}^2. \quad \text{(S17)}$$

The first derivative of $V_{GCV}(\lambda)$ is given by

$$\frac{dV_{GCV}(\lambda)}{d\lambda} = 2\alpha^2 \frac{W(\lambda)}{\left[\sum_{k=1}^K \frac{1}{(\sigma_k+\alpha\lambda)}\right]^3},$$

where the expression in nominator is as follows

$$W(\lambda) = -\left[\sum_{k=1}^K \frac{y_k^2}{\sigma_k(\sigma_k+\alpha\lambda)^3}\right]\left[\sum_{k=1}^K \frac{1}{(\sigma_k+\alpha\lambda)}\right] + \left[\sum_{k=1}^K \frac{1}{(\sigma_k+\alpha\lambda)^2}\right]\left[\sum_{k=1}^K \frac{y_k^2}{\sigma_k(\sigma_k+\alpha\lambda)^2}\right],$$

and, in view of the well known Chebyshev identity

$$\sum_{k=1}^K a_k \sum_{k=1}^K b_k = K \sum_{k=1}^K a_k b_k - \sum_{k=1}^K \sum_{j=k+1}^K (a_k - a_j)(b_k - b_j), \quad \text{(S18)}$$

after tedious algebraic manipulations, can be expressed as

$$W(\lambda) = \sum_{k=1}^K \sum_{j=k+1}^K \frac{(\sigma_k - \sigma_j)}{(\sigma_k+\alpha\lambda)^2(\sigma_j+\alpha\lambda)^2}\left[\frac{y_k^2}{\sigma_k(\sigma_k+\alpha\lambda)} - \frac{y_j^2}{\sigma_j(\sigma_j+\alpha\lambda)}\right]. \quad \text{(S19)}$$

Since, in view of the relations (S15) and (S2), we have $\bar{\bar{y}}_k = \frac{\sqrt{\alpha}}{\sqrt{\sigma_k}} y_k$, whence $y_k^2 = \frac{\sigma_k}{\alpha} \bar{\bar{y}}_k^2$, expression (S19) can be rewritten as

$$W(\lambda) = \frac{1}{\alpha} \sum_{k=1}^K \sum_{j=k+1}^K \frac{(\sigma_k - \sigma_j)}{(\sigma_k+\alpha\lambda)^3(\sigma_j+\alpha\lambda)^3}\left[\bar{\bar{y}}_k^2(\sigma_j + \alpha\lambda) - \bar{\bar{y}}_j^2(\sigma_k + \alpha\lambda)\right]. \quad \text{(S20)}$$

Since for $j > k$ we have $\sigma_k \geq \sigma_j$, inequalities (S16) and (S17), for any $\lambda \geq 0$, imply

$$\bar{\bar{y}}_k^2(\sigma_j + \alpha\lambda) \geq \bar{\bar{y}}_j^2(\sigma_k + \alpha\lambda).$$

Therefore, the nominator $W(\lambda)$ (S20) is positive (nonnegative) for any $\lambda \geq 0$. Therefore, if the Picard condition is satisfied, the GCV function (S7) is increasing

(non-decreasing) function and GCV method cannot be applied for direct identification method. However, for most tested examples the standard Picard condition is not satisfied, and the GCV function may be decreasing. Specifically, the numerical studies shown that for considered exemplary polymers the function $V_{GCV}(\lambda)$ monotonically decrease, which is illustrated by Figure S1, where $V_{GCV}(\lambda)$ (S7) is plotted for uni- and double-mode Gauss-like spectra. In Figure S2 the $V_{GCV}(\lambda)$ function is plotted for the exemplary KWW spectrum. These and all subsequent figures were drawn for the time-scale factors given in Tables 1-6 and the simulated stress relaxation experiment described in Subsections 2.6.1-2.6.3 of the main manuscript.

Summarizing, among many tests not a single example was found in which the application of the GCV technique would be possible.

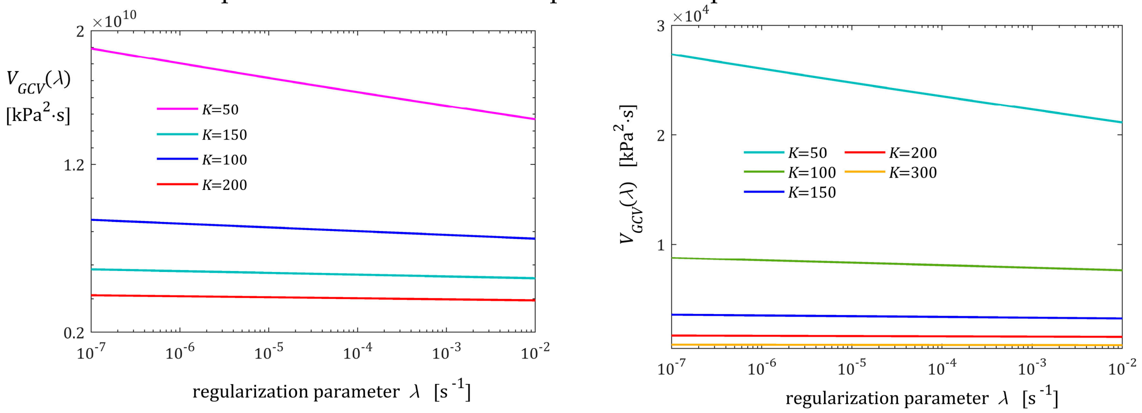

(**a**) (**b**)

Figure S1. The GCV function $V_{GCV}(\lambda)$ (S7) for: (**a**) uni-mode Gauss-like spectrum $\mathcal{H}(\tau)$ (32); (**b**) double-mode Gauss-like spectrum $\mathcal{H}(\tau)$ (36) for K relaxation modulus measurements.

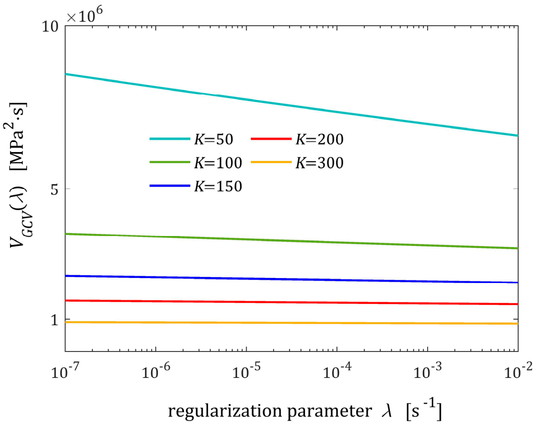

Figure S2. The GCV function $V_{GCV}(\lambda)$ (S7) for KWW spectrum $\mathcal{H}(\tau)$ (39) and K relaxation modulus measurements.

S.1.3. GCV Idea Applied to Direct Spectrum Identification Task

For any $t_k = \alpha k$ and any m, by (11) and (17), we have

$$\phi_m(t_k) = \frac{1}{\alpha k + \alpha m} = \varphi_{km} = \varphi_{mk},$$

whence the values of the relaxation modulus model $\bar{G}_K(t)$ (31) for $t = t_k = \alpha k$ can be described by

$$\bar{G}_K(t_k) = \sum_{k=1}^{K} \bar{g}_k(\lambda)\, \varphi_{mk},$$

whence, bearing in mind the notation of the matrix $\boldsymbol{\Phi}_K$ (19) and equation (22), the vector

$$\boldsymbol{G}_{MK} = \begin{bmatrix} \bar{G}_K(t_1) \\ \vdots \\ \bar{G}_K(t_K) \end{bmatrix},$$

corresponding to the measurements vector $\overline{\boldsymbol{G}}_K$ (19), can be written as follows (compare (46))

$$\boldsymbol{G}_{MK} = \frac{1}{\alpha}\boldsymbol{\Phi}_K \overline{\boldsymbol{g}}_K(\lambda) = \boldsymbol{\Phi}_K(\boldsymbol{\Phi}_K + \alpha\lambda\mathbb{I}_K)^{-1}\overline{\boldsymbol{G}}_K. \tag{S21}$$

Therefore, the vector of the relaxation modulus error (compare (S9)):

$$\boldsymbol{\varsigma}(\lambda) = \overline{\boldsymbol{G}}_K - \boldsymbol{G}_{MK} = \overline{\boldsymbol{G}}_K - \boldsymbol{\Phi}_K(\boldsymbol{\Phi}_K + \alpha\lambda\mathbb{I}_K)^{-1}\overline{\boldsymbol{G}}_K = \overline{\Xi}(\lambda)\overline{\boldsymbol{G}}_K, \tag{S22}$$

where matrix (compare $\Xi(\lambda)$ (S8))

$$\overline{\Xi}(\lambda) = \mathbb{I}_K - \boldsymbol{\Phi}_K(\boldsymbol{\Phi}_K + \alpha\lambda\mathbb{I}_K)^{-1}.$$

Let us define, by analogy to classic GCV function $V_{GCV}(\lambda)$ (S7), the modified GCV function as follows

$$V_{GCVm}(\lambda) = \frac{\|\varsigma(\lambda)\|_2^2}{\left[tr[\overline{\Xi}(\lambda)]\right]^2}. \tag{S23}$$

Since,

$$\overline{\Xi}(\lambda) = (\boldsymbol{\Phi}_K + \alpha\lambda\mathbb{I}_K)(\boldsymbol{\Phi}_K + \alpha\lambda\mathbb{I}_K)^{-1} - \boldsymbol{\Phi}_K(\boldsymbol{\Phi}_K + \alpha\lambda\mathbb{I}_K)^{-1},$$

yields

$$\overline{\Xi}(\lambda) = \alpha\lambda(\boldsymbol{\Phi}_K + \alpha\lambda\mathbb{I}_K)^{-1}, \tag{S24}$$

by (S22), we have

$$\boldsymbol{\varsigma}(\lambda) = \alpha\lambda(\boldsymbol{\Phi}_K + \alpha\lambda\mathbb{I}_K)^{-1}\overline{\boldsymbol{G}}_K,$$

whence immediately

$$\|\boldsymbol{\varsigma}(\lambda)\|_2^2 = (\alpha\lambda)^2\overline{\boldsymbol{G}}_K^T(\boldsymbol{\Phi}_K + \alpha\lambda\mathbb{I}_K)^{-1}(\boldsymbol{\Phi}_K + \alpha\lambda\mathbb{I}_K)^{-1}\overline{\boldsymbol{G}}_K,$$

which, combined with (S24) and (S23), results in

$$V_{GCVm}(\lambda) = \frac{(\alpha\lambda)^2\overline{\boldsymbol{G}}_K^T(\boldsymbol{\Phi}_K+\alpha\lambda\mathbb{I}_K)^{-1}(\boldsymbol{\Phi}_K+\alpha\lambda\mathbb{I}_K)^{-1}\overline{\boldsymbol{G}}_K}{(\alpha\lambda)^2 tr[(\boldsymbol{\Phi}_K+\alpha\lambda\mathbb{I}_K)^{-1}]^2} = \frac{\overline{\boldsymbol{G}}_K^T(\boldsymbol{\Phi}_K+\alpha\lambda\mathbb{I}_K)^{-1}(\boldsymbol{\Phi}_K+\alpha\lambda\mathbb{I}_K)^{-1}\overline{\boldsymbol{G}}_K}{tr[(\boldsymbol{\Phi}_K+\alpha\lambda\mathbb{I}_K)^{-1}]^2}. \tag{S25}$$

The above formula differs from the classic GCV function $V_{GCV}(\lambda)$ (S13) with the absence of ill-conditioned matrix $\boldsymbol{\Phi}_K^{-1}$ in the square form in nominator.

By (S14), (S25), (26), (27) and orthogonality of $\boldsymbol{U}_K$ we have

$$V_{GCVm}(\lambda) = \frac{\overline{\boldsymbol{G}}_K^T\boldsymbol{U}_K\boldsymbol{\Omega}_K(\lambda)\boldsymbol{\Omega}_K(\lambda)\boldsymbol{U}_K^T\overline{\boldsymbol{G}}_K}{tr\left[\boldsymbol{U}_K\boldsymbol{\Omega}_K(\lambda)\boldsymbol{U}_K^T\right]^2} = \frac{\boldsymbol{Y}_K^T\boldsymbol{\Omega}_K(\lambda)\boldsymbol{\Omega}_K(\lambda)\boldsymbol{Y}_K}{tr[\boldsymbol{\Omega}_K(\lambda)]^2} = \frac{\sum_{k=1}^K\frac{y_k^2}{(\sigma_k+\alpha\lambda)^2}}{\left[\sum_{k=1}^K\frac{1}{(\sigma_k+\alpha\lambda)}\right]^2},$$

where both the denominator and nominator are strictly monotonically decreasing functions of the regularization parameter. The first derivative of $V_{GCVm}(\lambda)$ is as follows

$$\frac{dV_{GCVm}(\lambda)}{d\lambda} = 2\alpha\frac{W_m(\lambda)}{\left[\sum_{k=1}^K\frac{1}{(\sigma_k+\alpha\lambda)}\right]^3},$$

where the expression in nominator is given by

$$W_m(\lambda) = -\left[\sum_{k=1}^K\frac{y_k^2}{(\sigma_k+\alpha\lambda)^3}\right]\left[\sum_{k=1}^K\frac{1}{(\sigma_k+\alpha\lambda)}\right] + \left[\sum_{k=1}^K\frac{1}{(\sigma_k+\alpha\lambda)^2}\right]\left[\sum_{k=1}^K\frac{y_k^2}{(\sigma_k+\alpha\lambda)^2}\right],$$

and using the Chebyshev identity (S18) can be written as (compare (S19))

$$W_m(\lambda) = \sum_{k=1}^{K}\sum_{j=k+1}^{K}\frac{(\sigma_k-\sigma_j)}{(\sigma_k+\alpha\lambda)^2(\sigma_j+\alpha\lambda)^2}\left[\frac{y_k^2}{(\sigma_k+\alpha\lambda)}-\frac{y_j^2}{(\sigma_j+\alpha\lambda)}\right],$$

which, by $y_k^2 = \frac{\sigma_k}{\alpha}\bar{\bar{y}}_k^2$, yields

$$W_m(\lambda) = \frac{1}{\alpha}\sum_{k=1}^{K}\sum_{j=k+1}^{K}\frac{(\sigma_k-\sigma_j)}{(\sigma_k+\alpha\lambda)^2(\sigma_j+\alpha\lambda)^2}\left[\frac{\sigma_k\bar{\bar{y}}_k^2}{(\sigma_k+\alpha\lambda)}-\frac{\sigma_j\bar{\bar{y}}_j^2}{(\sigma_j+\alpha\lambda)}\right],$$

where the expressions in the square brackets are positive (non-negative) for any k whenever the Picard Condition 1 is satisfied. The modified GCV function is monotonically increasing for this standard condition. Also the numerical studies shown, that for considered exemplary Gauss-like and KWW spectra, function $V_{GCVm}(\lambda)$ monotonically increases, which is illustrated by Figures S3 and S4. Summarizing, both in the case of the Picard Condition 1 or not, the classic GCV and modified GCV method are not applicable for direct spectrum identification.

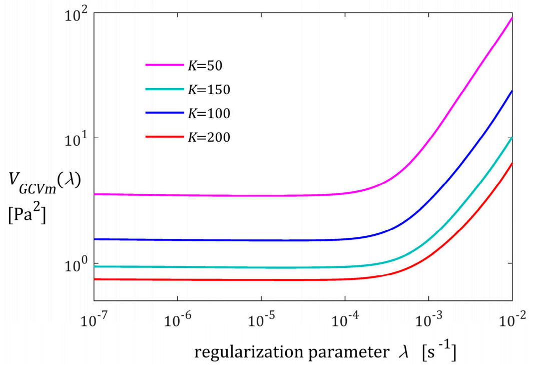

(**a**)

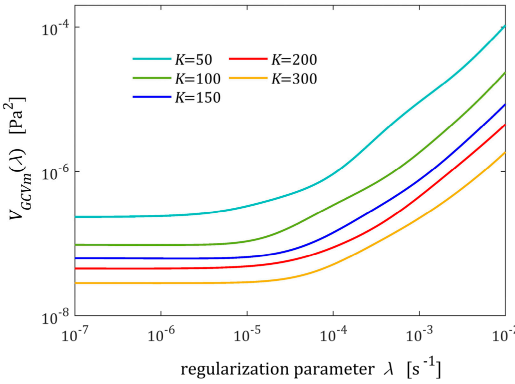

(**b**)

Figure S3. The GCV function $V_{GCVm}(\lambda)$ (S23) for: (**a**) uni-mode Gauss-like spectrum $\mathcal{H}(\tau)$ (32), (**b**) double-mode Gauss-like spectrum $\mathcal{H}(\tau)$ (36) for K relaxation modulus measurements.

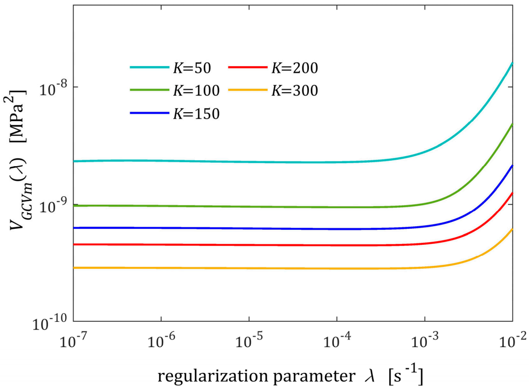

Figure S4. The GCV function $V_{GCVm}(\lambda)$ (S23) for KWW spectrum $\mathcal{H}(\tau)$ (39) for K relaxation modulus measurements.

S.1.4. Applicability of the L-Curve Method

For the regularized least-squares task (S5) the L-curve is the plot of residual norm $\|\boldsymbol{\varrho}(\lambda)\|_2$ versus solution norm $\|\bar{\boldsymbol{g}}_K(\lambda)\|_2$ in logarithmic scales parameterized by the regularization parameter, where $\boldsymbol{\varrho}(\lambda)$ is given by (S9). Introducing the notations

$$x(\lambda) = \log\|\boldsymbol{\varrho}(\lambda)\|_2, \quad y(\lambda) = \log\|\bar{\boldsymbol{g}}_K(\lambda)\|_2, \tag{S26}$$

where log denotes the natural logarithm, the L-curve is a plot of $y(\lambda)$ as a function of $x(\lambda)$. Combining (S11)-(S14), (24), (26), (27), by the orthogonality of $\boldsymbol{U}_K$, we obtain

$$\|\boldsymbol{\varrho}(\lambda)\|_2^2 = \alpha \overline{\boldsymbol{G}}_K^T \boldsymbol{\Theta}(\lambda) \overline{\boldsymbol{G}}_K = \alpha(\alpha\lambda)^2 \boldsymbol{Y}_K^T \boldsymbol{\Omega}_K(\lambda) \boldsymbol{\Sigma}_K^{-1} \boldsymbol{\Omega}_K(\lambda) \boldsymbol{Y}_K = \alpha(\alpha\lambda)^2 \sum_{k=1}^{K} \frac{y_k^2}{\sigma_k(\sigma_k+\alpha\lambda)^2}. \quad \text{(S27)}$$

Therefore, functions $x(\lambda)$ and $y(\lambda)$ are as follows

$$x(\lambda) = \log\|\boldsymbol{\varrho}(\lambda)\|_2 = \frac{3}{2}\log\alpha + \log\lambda + \frac{1}{2}\log\left[\sum_{k=1}^{K} \frac{y_k^2}{\sigma_k(\sigma_k+\alpha\lambda)^2}\right],$$

and

$$y(\lambda) = \log\|\overline{\boldsymbol{g}}_K(\lambda)\|_2 = \log\alpha + \frac{1}{2}\log\left[\sum_{k=1}^{K} \frac{y_k^2}{(\sigma_k+\alpha\lambda)^2}\right],$$

where the last formula results directly from equation (50).

The L-curve method consists in choosing the parameter λ that maximizes the curvature index of this curve. However the studies conducted for Gauss-like and KWW spectra show that the shape of the L-curve precludes the use of this method. The courses of the L-curve for Gauss-like spectra are shown in Figures S5 and S6; these plots are parameterized by the regularization parameter $1.1 \cdot 10^{-7} \leq \lambda \leq 10^{-1}$ [s^{-1}].

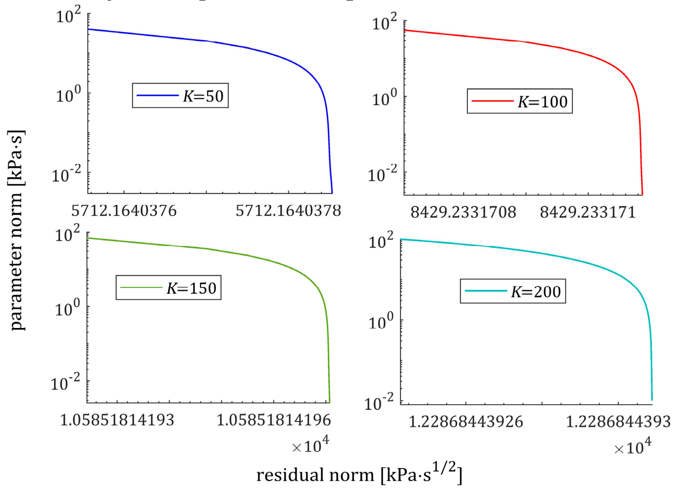

Figure S5. The L-curve: the plot of residual norm $\|\boldsymbol{\varrho}(\lambda)\|_2$ (S27) versus the parameter norm $\|\overline{\boldsymbol{g}}_K(\lambda)\|_2$ (50), for uni-mode Gauss-like spectrum $\mathcal{H}(\tau)$ (32) and K relaxation modulus measurements.

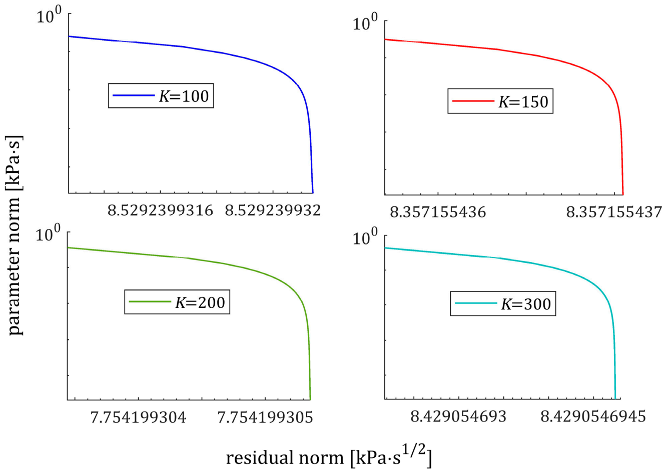

Figure S6. The L-curve: the residual norm $\|\boldsymbol{\varrho}(\lambda)\|_2$ (S27) versus the parameter norm $\|\overline{\boldsymbol{g}}_K(\lambda)\|_2$ (50), for double-mode Gauss-like spectrum $\mathcal{H}(\tau)$ (36) and K relaxation modulus measurements.

A quick inspection of the above figures shows that for small λ the norm of residual $\|\boldsymbol{\varrho}(\lambda)\|_2 = \left\|\overline{\overline{\boldsymbol{G}}}_K - \frac{1}{\sqrt{\alpha}}\boldsymbol{\Psi}_K \overline{\boldsymbol{g}}_K(\lambda)\right\|_2$ grows but the parameter norm $\|\overline{\boldsymbol{g}}_K(\lambda)\|_2$ does not de-

crease, which, in turn, rapidly decrees only for large norm of residual. This mean that the idea of the L-curve method, which relies in the rapid decrees of $\|\overline{\boldsymbol{g}}_K(\lambda)\|_2$ for small $\|\boldsymbol{\varrho}(\lambda)\|_2$ and slow decrees of $\|\overline{\boldsymbol{g}}_K(\lambda)\|_2$ for growing $\left\|\overline{\overline{\boldsymbol{G}}}_K - \frac{1}{\sqrt{\alpha}}\boldsymbol{\Psi}_K\overline{\boldsymbol{g}}_K(\lambda)\right\|_2$, is lost here.

S.1.5. Applicability of the L-Curve Idea to Direct Spectrum Identification

Let us apply an idea of the L-Curve method to the relaxation modulus error $\boldsymbol{\varsigma}(\lambda)$ (S22), being residual of the relaxation modulus model, and optimal model parameter $\overline{\boldsymbol{g}}_K(\lambda)$, i.e., consider $\|\boldsymbol{\varsigma}(\lambda)\|_2$ versus $\|\overline{\boldsymbol{g}}_K(\lambda)\|_2$ in logarithmic scales. Comparing the left equality in (S22), where $\boldsymbol{G}_{MK}$ is given by (S21), with the left equality in (46) we see that

$$\|\boldsymbol{\varsigma}(\lambda)\|_2^2 = KQ_K\big(\overline{\boldsymbol{g}}_K(\lambda)\big),$$

which, by (51), yields

$$\|\boldsymbol{\varsigma}(\lambda)\|_2^2 = K\lambda^2\|\overline{\boldsymbol{g}}_K(\lambda)\|_2^2, \tag{S28}$$

whence the next equation follows

$$\log\|\boldsymbol{\varsigma}(\lambda)\|_2 = \log(\lambda) + \log\big(\sqrt{K}\big) + \log\|\overline{\boldsymbol{g}}_K(\lambda)\|_2, \tag{S29}$$

The courses of the L-curve for Gauss-like and KWW spectra are shown in Figures S7-S9; all these plots are parameterized by the regularization parameter $1.1\cdot 10^{-7} \le \lambda \le 10^{-1}$ [s^{-1}].

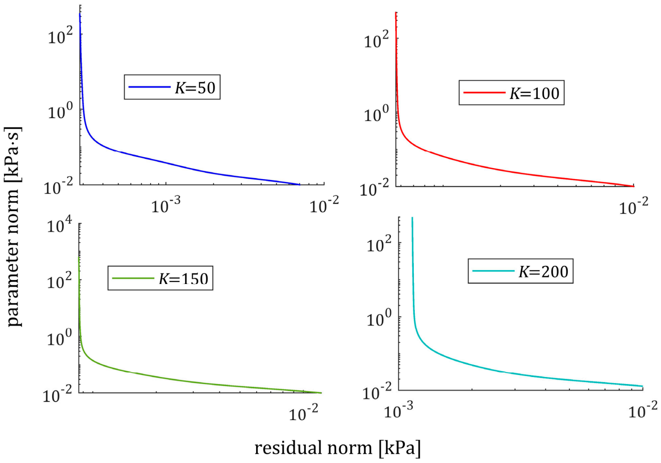

Figure S7. The L-curve: residual norm $\|\boldsymbol{\varsigma}(\lambda)\|_2$ (S28) versus parameter norm $\|\overline{\boldsymbol{g}}_K(\lambda)\|_2$ (50), for uni-mode Gauss-like spectrum $\mathcal{H}(\tau)$ (32) and K relaxation modulus measurements.

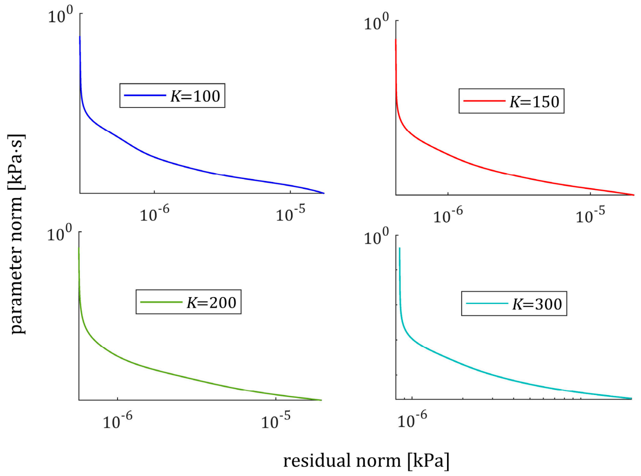

Figure S8. The L-curve: residual norm $\|\boldsymbol{\varsigma}(\lambda)\|_2$ (S28) versus parameter norm $\|\overline{\boldsymbol{g}}_K(\lambda)\|_2$ (50), for double-mode Gauss-like spectrum $\mathcal{H}(\tau)$ (36) and K relaxation modulus measurements.

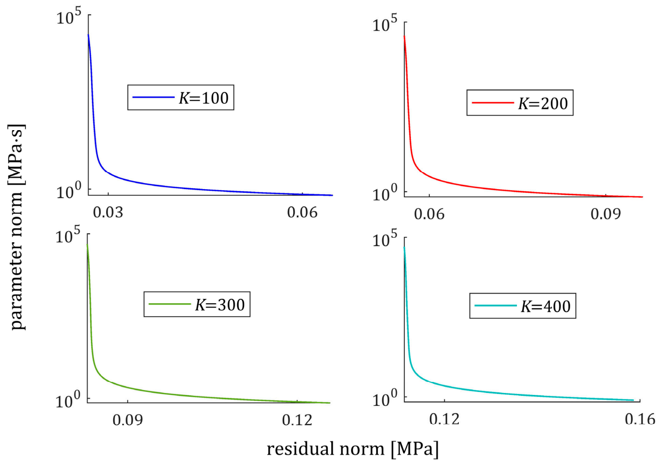

Figure S9. The L-curve: residual norm $\|\boldsymbol{\varsigma}(\lambda)\|_2$ (S28) versus parameter norm $\|\overline{\boldsymbol{g}}_K(\lambda)\|_2$ (50), for KWW spectrum $\mathcal{H}(\tau)$ (39) and K relaxation modulus measurements.

The curves shown above do not exclude the possibility of the L-Curve method application. Whence, introducing the function

$$x(\lambda) = \log\|\boldsymbol{\varsigma}(\lambda)\|_2,$$

we can determine the curvature index for the L-curve $\left(x(\lambda), y(\lambda)\right)$, which is given by the formula [37]

$$\mathcal{K}(\lambda) = \frac{|\dot{x}(\lambda)\ddot{y}(\lambda) - \ddot{x}(\lambda)\dot{y}(\lambda)|}{[[\dot{x}(\lambda)]^2 + [\dot{y}(\lambda)]^2]^{3/2}}, \quad \text{(S30)}$$

where $y(\lambda)$ is defined by (S26).

From (S29) we have

$$y(\lambda) = x(\lambda) - \log(\lambda) - \log\left(\sqrt{K}\right).$$

Therefore,

$$\dot{y}(\lambda) = \dot{x}(\lambda) - \frac{1}{\lambda},$$

and

$$\ddot{y}(\lambda) = \ddot{x}(\lambda) + \frac{1}{\lambda^2},$$

whence the curvature index (S30) is as follows

$$\mathcal{K}(\lambda) = \frac{\left|\dot{x}(\lambda)\left[\ddot{x}(\lambda) + \frac{1}{\lambda^2}\right] - \ddot{x}(\lambda)\left[\dot{x}(\lambda) - \frac{1}{\lambda}\right]\right|}{\left[[\dot{x}(\lambda)]^2 + \left[\dot{x}(\lambda) - \frac{1}{\lambda}\right]^2\right]^{3/2}} = \frac{|\dot{x}(\lambda) + \lambda\,\ddot{x}(\lambda)|}{\lambda^2\left[[\dot{x}(\lambda)]^2 + \left[\dot{x}(\lambda) - \frac{1}{\lambda}\right]^2\right]^{3/2}}. \quad \text{(S31)}$$

According to the L-Curve method, the maximum of the curvature determines the optimal regularization parameter. The courses of curvature index $\mathcal{K}(\lambda)$ (S31) for uni-mode Gauss-like relaxation spectrum are depicted in Figure S10, from which it is seen that the curvature $\mathcal{K}(\lambda)$ has small (not greater than 0.6) local maxima but none of them dominates. Therefore, the L-curve method cannot be applied here. The curvature courses for double-mode Gauss-like spectrum (36) are similar. The uni-mode curvatures for KWW spectrum (39) are plotted in Figure S11; however the maxima of the curvatures $\mathcal{K}(\lambda)$ do not exceed 1.1 and, simultaneously, the regularization parameters λ_{max} maximizing the curvature index $\mathcal{K}(\lambda)$ are too big, their values are given in Table S1. In consequence, too smoothed relaxation spectra models are obtained. Summarizing, the

L-curve method cannot be successfully applied for direct spectrum identification. However, the L-curve tool, allows us to understand and graphically illustrate the dependence of the model quality indices on the regularization parameter.

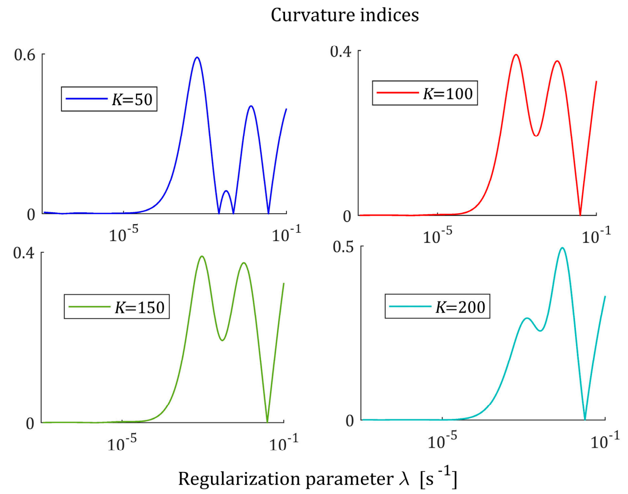

Figure S10. The curvature index $\mathcal{K}(\lambda)$ (S31) of the L-curve: residual norm $\|\boldsymbol{\varsigma}(\lambda)\|_2$ versus parameter norm $\|\overline{\boldsymbol{g}}_K(\lambda)\|_2$ (50), for one-mode Gauss-like spectrum $\mathcal{H}(\tau)$ (32) and K relaxation modulus measurements.

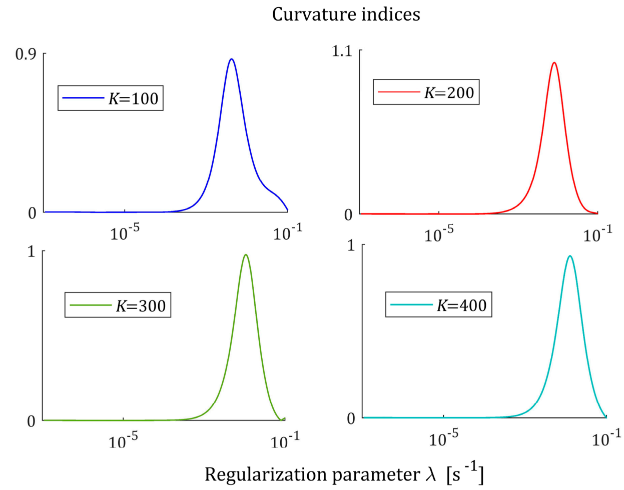

Figure S11. The curvature index $\mathcal{K}(\lambda)$ (S31) of the L-curve: residual norm $\|\boldsymbol{\varsigma}(\lambda)\|_2$ versus parameter norm $\|\overline{\boldsymbol{g}}_K(\lambda)\|_2$ (50), for KWW spectrum $\mathcal{H}(\tau)$ (39) and K relaxation modulus measurements. Time-scale factors are given in Table S1.

Table S1. For the KWW spectrum $\mathcal{H}(\tau)$ (39): time-scale factors α, numbers of model summands K, regularization parameters λ_{max} maximizing the curvature index $\mathcal{K}(\lambda)$ (S31) of the L-curve: residual norm $\|\boldsymbol{\varsigma}(\lambda)\|_2$ versus parameter norm $\|\overline{\boldsymbol{g}}_K(\lambda)\|_2$ (50), and the maximal values $\mathcal{K}(\lambda_{max})$ of the curvature $\mathcal{K}(\lambda)$.

$\boldsymbol{K}$	$\boldsymbol{\alpha}$ $[\boldsymbol{s}]$	$\boldsymbol{\lambda}$ $[\boldsymbol{s^{-1}}]$	$\boldsymbol{\lambda_{max}}$ $[\boldsymbol{s^{-1}}]$	$\boldsymbol{\mathcal{K}(\lambda_{max})}$
25	0.8	2×10^{-5}	2.50261×10^{-3}	1.163379
50	0.65	7×10^{-5}	5.72351×10^{-3}	0.804296
75	0.6	7.5×10^{-5}	3.61311×10^{-3}	0.644996
100	0.65	8.5×10^{-5}	4.17921×10^{-3}	0.867875
150	0.6	1×10^{-4}	6.30731×10^{-3}	0.983846
200	0.6	1.5×10^{-4}	8.03801×10^{-3}	1.015725
300	0.55	1.6×10^{-4}	1.074731×10^{-2}	0.975800
400	0.55	1.6×10^{-4}	1.267871×10^{-2}	0.933897

S.2. Plots of the Multiplicative Regularization Indices

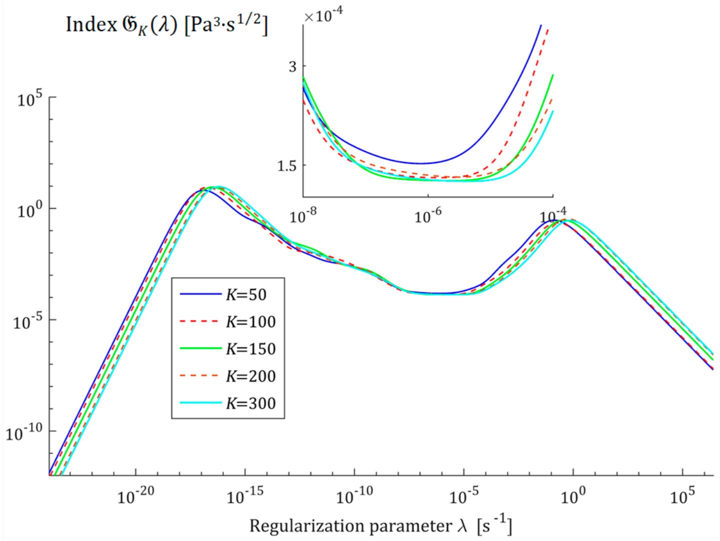

Figure S12. Multiplicative Regularization Index $\mathfrak{G}_K(\lambda)$ (52) as the function of the regularization parameter λ for K relaxation modulus measurements corrupted by additive independent noises uniformly distributed on the interval $[-0.005, 0.005]$ Pa for the double-mode Gauss-like time relaxation spectrum $\mathcal{H}(\tau)$ (36).

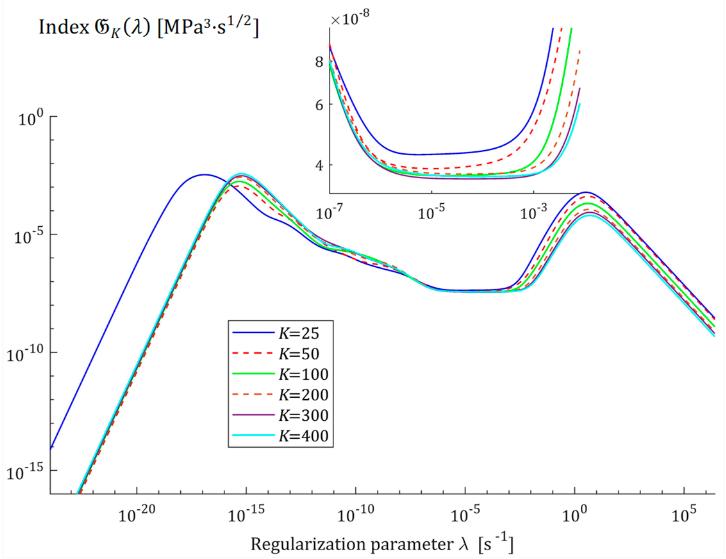

Figure S13. Multiplicative Regularization Index $\mathfrak{G}_K(\lambda)$ (52) for as the function of the regularization parameter λ for K measurements of the relaxation modulus corrupted by additive independent noises uniformly distributed on the interval $[-0.5, 0.5]$ kPa for the KWW relaxation spectrum $\mathcal{H}(\tau)$ (39).

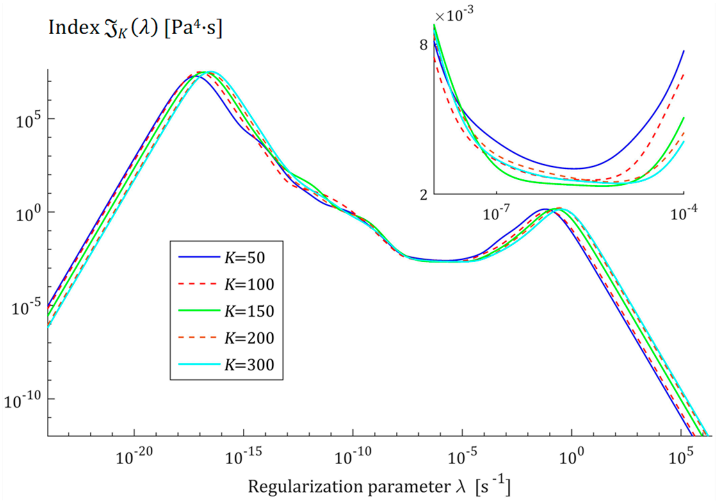

Figure S14. Square Multiplicative Regularization Index $\mathfrak{J}_K(\lambda)$ (53) as the function of the regularization parameter λ for K relaxation modulus measurements corrupted by additive independent noises uniformly distributed on the interval $[-0.005, 0.005]$ Pa for the double-mode Gauss-like time relaxation spectrum $\mathcal{H}(\tau)$ (36).

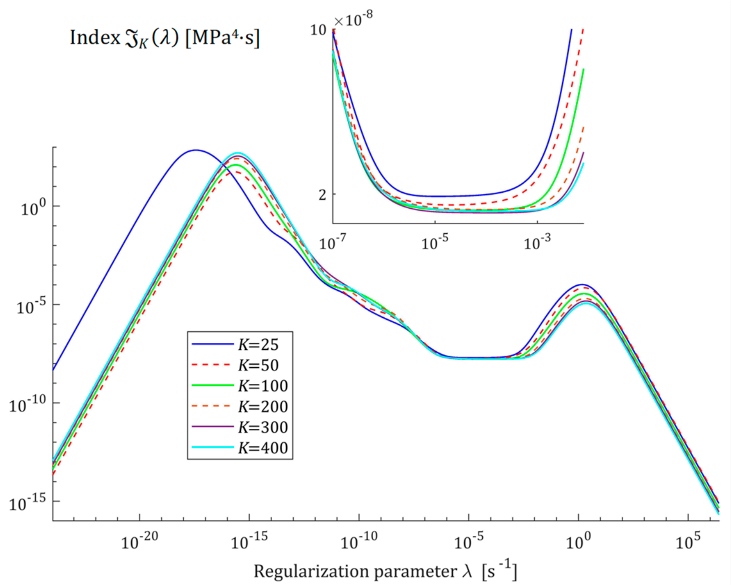

Figure S15. Square Multiplicative Regularization Index $\mathfrak{J}_K(\lambda)$ (53) as the function of the regularization parameter λ for K measurements of the relaxation modulus corrupted by additive independent noises uniformly distributed on the interval $[-0.5, 0.5]$ kPa for the KWW relaxation spectrum $\mathcal{H}(\tau)$ (39).

S.3. Plots of the Optimal Models of the KWW Relaxation Spectra

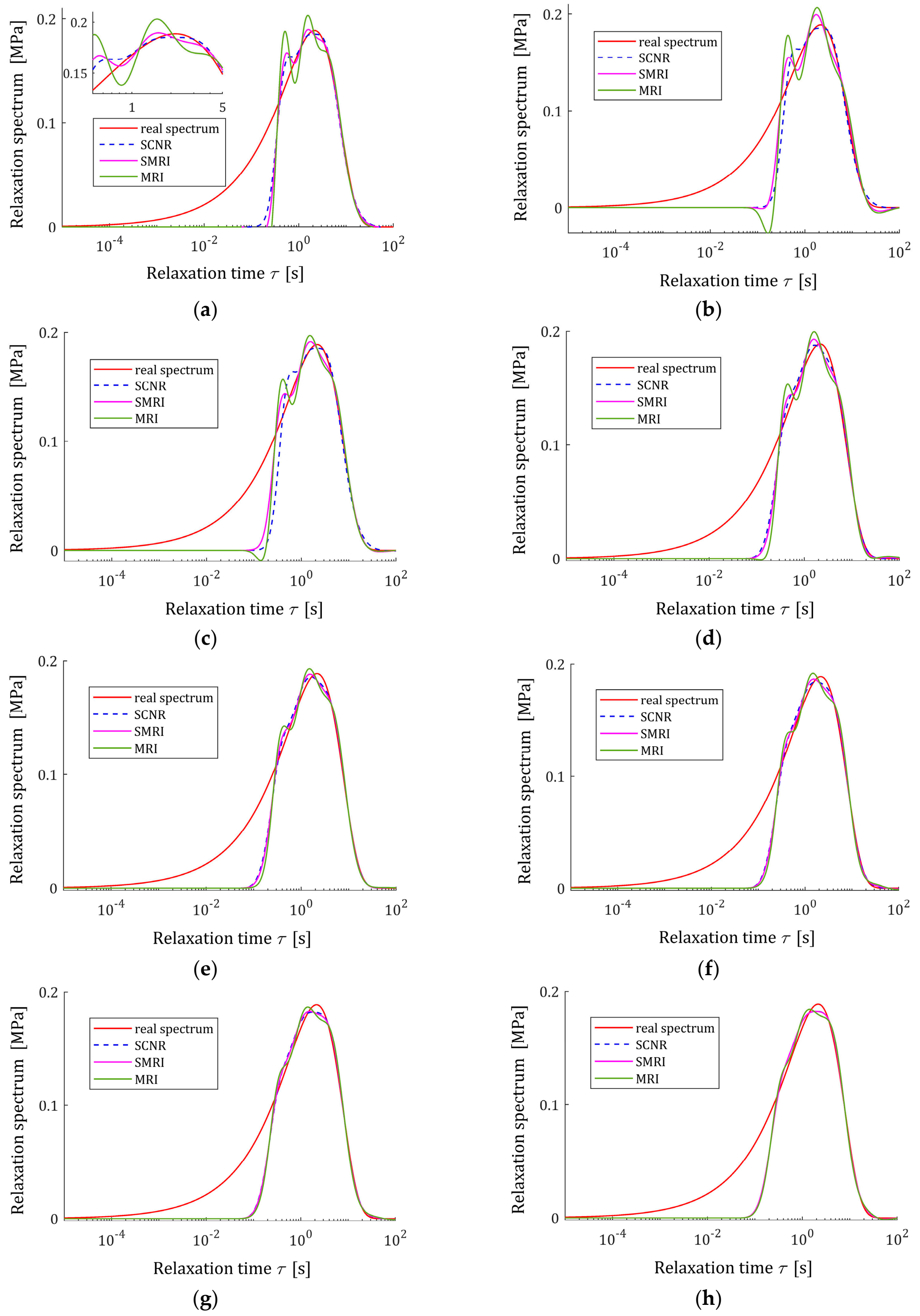

Figure S16. KWW spectrum $\mathcal{H}(\tau)$ (39) (solid red line) and the corresponding models $\overline{\mathcal{H}}_K(\tau)$ (30) for K measurements of the relaxation modulus corrupted by additive independent noises uniformly distributed on the interval $[-0.5, 0.5]$ kPa determined for regularization parameters computed using MRI (58), SMRI (59) and SCNR (72) rules: (**a**) $K = 25$; (**b**) $K = 50$; (**c**) $K = 75$; (**d**) $K = 100$; (**e**) $K = 150$; (**f**) $K = 200$; (**g**) $K = 300$; (**h**) $K = 400$.

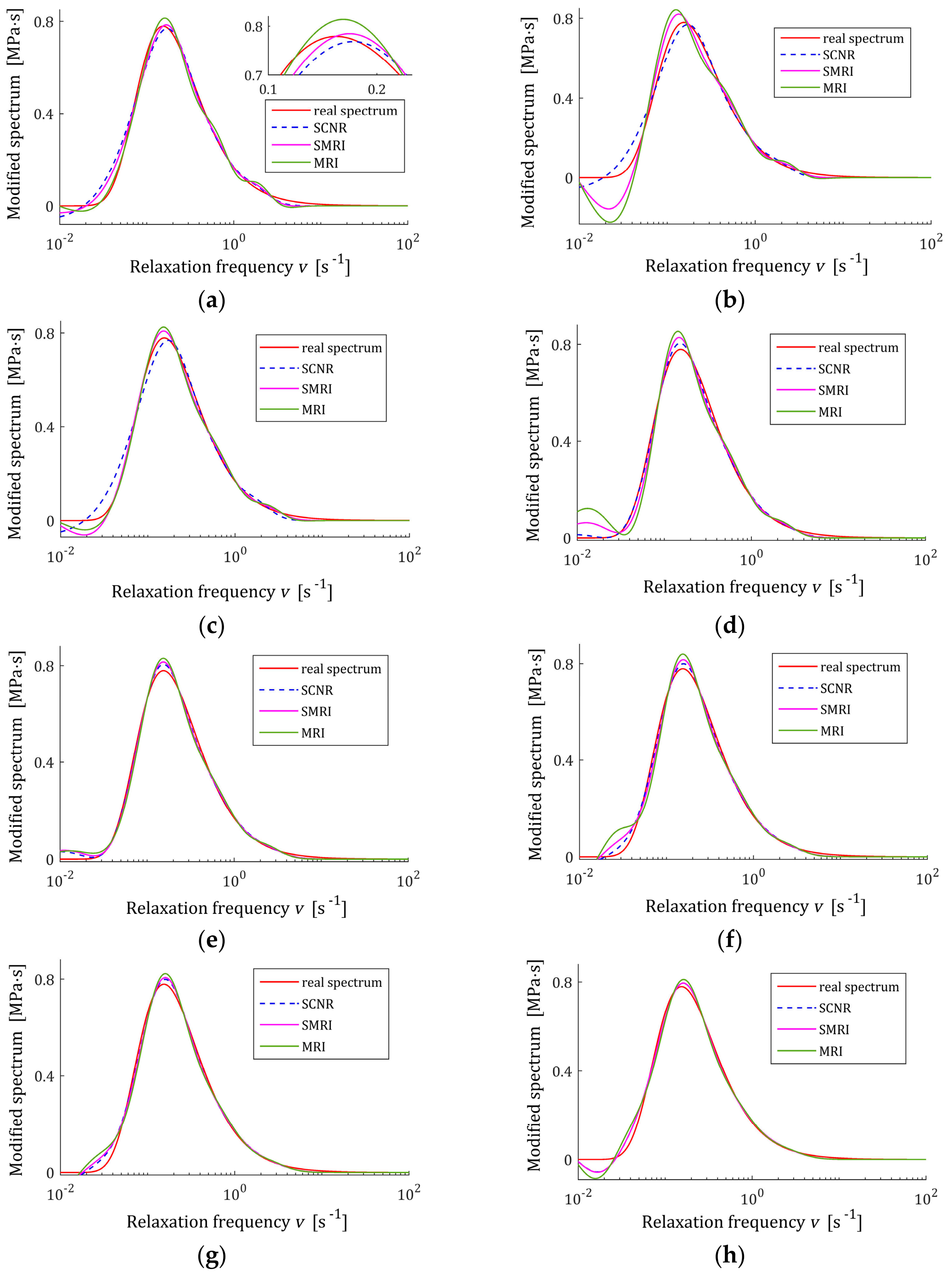

Figure S17. Modified KWW relaxation spectrum $H^M(v)$ (41) (solid red line) and the corresponding models $\bar{H}_K^M(v)$ (28) for K measurements of the relaxation modulus corrupted by additive independent noises uniformly distributed on the interval $[-0.5, 0.5]$ kPa determined for regularization parameters computed using MRI (58), SMRI (59) and SCNR (72) rules: (**a**) $K = 25$; (**b**) $K = 50$; (**c**) $K = 75$; (**d**) $K = 100$; (**e**) $K = 150$; (**f**) $K = 200$; (**g**) $K = 300$; (**h**) $K = 400$.

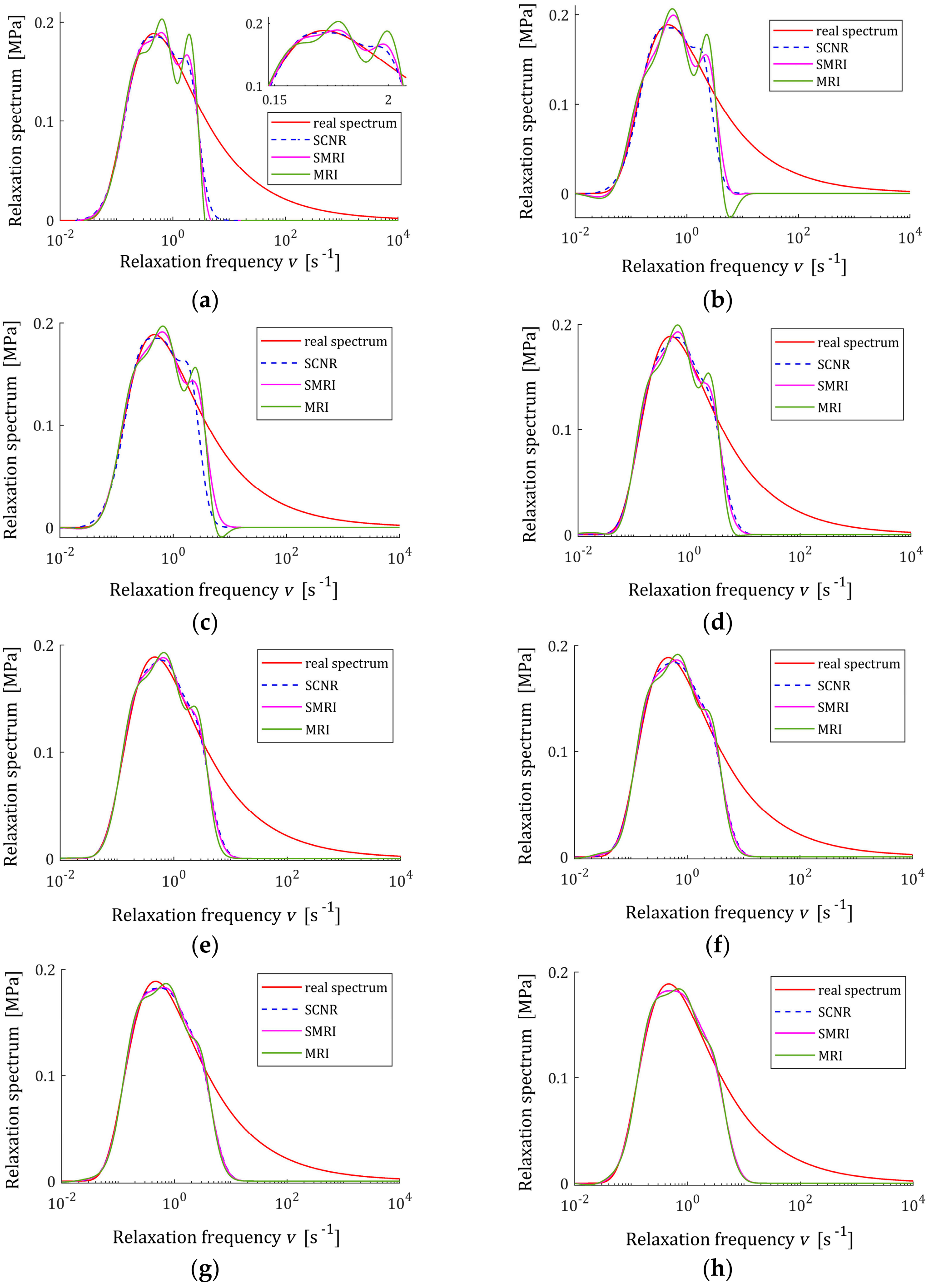

Figure S18. KWW relaxation frequency spectrum $H(v)$ (40) (solid red line) and the corresponding models $\overline{H}_K(v)$ (29) for K measurements of the relaxation modulus corrupted by additive independent noises uniformly distributed on the interval $[-0.5, 0.5]$ kPa determined for regularization parameters computed using MRI (58), SMRI (59) and SCNR (72) rules: (**a**) $K = 25$; (**b**) $K = 50$; (**c**) $K = 75$; (**d**) $K = 100$; (**e**) $K = 150$; (**f**) $K = 200$; (**g**) $K = 300$; (**h**) $K = 400$.

References

Literature references and equations are consistent with the content of the main manuscript.
